# Esr1^+^ hypothalamic-habenula neurons shape aversive states

**DOI:** 10.1038/s41593-023-01367-8

**Published:** 2023-06-22

**Authors:** Daniela Calvigioni, Janos Fuzik, Pierre Le Merre, Marina Slashcheva, Felix Jung, Cantin Ortiz, Antonio Lentini, Veronika Csillag, Marta Graziano, Ifigeneia Nikolakopoulou, Moritz Weglage, Iakovos Lazaridis, Hoseok Kim, Irene Lenzi, Hyunsoo Park, Björn Reinius, Marie Carlén, Konstantinos Meletis

**Affiliations:** 1 Department of Neuroscience, Karolinska Institutet, Stockholm, Sweden; 2 Department of Medical Biochemistry and Biophysics, Karolinska Institutet, Stockholm, Sweden

**Keywords:** Limbic system, Neural circuits

## Abstract

Excitatory projections from the lateral hypothalamic area (LHA) to the lateral habenula (LHb) drive aversive responses. We used patch-sequencing (Patch-seq) guided multimodal classification to define the structural and functional heterogeneity of the LHA–LHb pathway. Our classification identified six glutamatergic neuron types with unique electrophysiological properties, molecular profiles and projection patterns. We found that genetically defined LHA–LHb neurons signal distinct aspects of emotional or naturalistic behaviors, such as estrogen receptor 1-expressing (Esr1^+^) LHA–LHb neurons induce aversion, whereas neuropeptide Y-expressing (Npy^+^) LHA–LHb neurons control rearing behavior. Repeated optogenetic drive of Esr1^+^ LHA–LHb neurons induces a behaviorally persistent aversive state, and large-scale recordings showed a region-specific neural representation of the aversive signals in the prelimbic region of the prefrontal cortex. We further found that exposure to unpredictable mild shocks induced a sex-specific sensitivity to develop a stress state in female mice, which was associated with a specific shift in the intrinsic properties of bursting-type Esr1^+^ LHA–LHb neurons. In summary, we describe the diversity of LHA–LHb neuron types and provide evidence for the role of Esr1^+^ neurons in aversion and sexually dimorphic stress sensitivity.

## Abstract (web summary)

The authors find a surprising diversity in hypothalamic neurons projecting to habenula, and using patch-sequencing (Patch-seq), identify an estrogen receptor-expressing neuron type that signals aversion and is linked to stress in female mice.

## Main

Behaviors based on emotional processing (emotional behaviors) are regulated by internal and external values and state signals, where negative signals, for example, lead to avoidance^1^. Emotional behaviors and affective disorders are thought to depend on subcortical, as well as cortical circuitries, where the lateral habenula (LHb) and the prefrontal cortex (PFC) are key nodes in value and emotion processing^2^. The PFC is a key network controlling cognitive and emotional behavior^3,4^ and the LHb is known to integrate value and emotion signals^5^. Notably, the main excitatory input to LHb originates from the lateral hypothalamic area (LHA), and this LHA–LHb pathway can signal avoidance and the expression of depression-like states^6–9^. Affective disorders, such as depression, are more common in women,^10^ and the sensitivity to negative events shows sex differences^11,12^. The hypothalamus is a highly heterogeneous region, composed of many subregions^13^ containing different neuron types defined by differential gene expression^14^ and projection pattern^15,16^, including sexually dimorphic circuits and behavior^17,18^. In spite of the evident cellular and functional diversity in other hypothalamic regions, the LHA–LHb pathway has so far been studied as a homogeneous glutamatergic population^6–8,19,20^.

To define the organization and function of the glutamatergic (Vglut2^+^) LHA–LHb pathway, we used a multimodal classification of neuron types based on patch-sequencing (Patch-seq) to establish the electrophysiological, neuroanatomical and molecular diversity of Vglut2^+^ LHA–LHb neurons. In contrast to the notion of a homogenous LHA–LHb pathway, we found that the LHA–LHb pathway is composed of several neuron types characterized by discrete intrinsic properties, molecular markers and spatial projection patterns. Cell-type-specific optogenetic manipulations revealed that negative signals and persistent negative states are mediated through estrogen receptor 1-expressing (Esr1^+^) LHA–LHb neurons, whereas neuropeptide Y-expressing (Npy^+^) LHA–LHb neurons control rearing behavior. Neuropixels recordings in the PFC highlighted that induction of negative signals through repeated activation of Esr1^+^ LHA–LHb neurons leads to persistent shifts in the PFC neural activity, revealing a central role for the prelimbic area (PL) in encoding the negative state. Further supporting the role of Esr1^+^ LHA–LHb neurons in persistent negative emotional states, we found that increased stress sensitivity in female mice was dependent on the activity of Esr1^+^ LHA–LHb neurons and associated with a persistent shift in the intrinsic properties of the bursting-type Esr1^+^ LHA–LHb neurons. Altogether, the excitatory LHA–LHb pathway is composed of several discretely organized neuron subtypes with unique functions, where Esr1^+^ LHA–LHb neurons are central in mediating negative signals and stress sensitivity.

## Electrophysiological diversity of LHA–LHb neurons

**Fig. 1:**
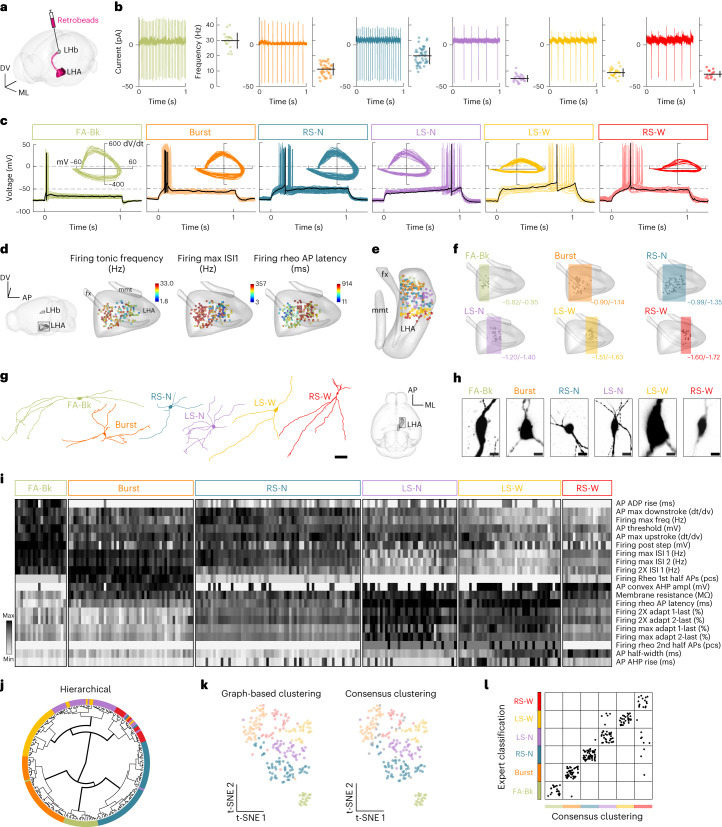
Electrophysiological diversity of LHA–LHb neurons. **a**, Strategy for retrograde labeling of LHA–LHb neurons. **b**, Cell-attached electrophysiological recordings reveal tonic firing of LHA–LHb neurons. Cell-attached traces from representative neurons (left) and the mean firing rate of the individual neurons (mean ± s.d.; right). *n*_neuron_ = 230, from left to right *n*_neuron_ = 20, 49, 65, 37, 40, 19 and *n*_mice_ = 46 WT. **c**, Overlay of the individual whole-cell traces of firing at rheobase (Rheo) of all recorded LHA–LHb neurons. Black represents a trace from a representative neuron. Inset, phase-plane plots of the first AP at rheobase for all individual neurons. **d**, Electrophysiological properties reveal anatomical organization of LHA–LHb neurons. Dots represent recorded LHA–LHb neurons color coded by different electrophysiological parameters. **e**, Three-dimensional position of all recorded LHA–LHb neurons (color coded by cell type). **f**, Three-dimensional visualization of the A-P distribution of electrophysiologically characterized LHA–LHb neuron types. Bregma coordinates show the most anterior and posterior coordinates for each subtype. **g**, Reconstruction of representative dendritic morphologies of LHA–LHb neuron types. **h**, Images of representative soma morphologies of the LHA–LHb neuron types. **i**, Heatmap of 20 electrophysiological parameters selected based on PCA. Classification of LHA–LHb neuron types by expert classification, as in **c**. One column = one LHA–LHb neuron. **j**, Circular dendrogram for hierarchical clustering of LHA–LHb neurons. Color code, expert classification of LHA–LHb neurons. **k**, t-distributed stochastic neighbor embedding (t-SNE) plots of graph-based clustering (left), and consensus clustering (right). Color code, consensus clustering of LHA–LHb neurons. **l**, Agreement between the expert classification and the unsupervised consensus clustering of LHA–LHb neurons. All data acquired in male mice. Scale bar, 50 μm (**g**), 10 μm (**h**). See also Extended Data Figs. 1 and 2 and Supplementary Tables 1–3. *n*_neuron_ = number of neurons; *n*_mice_ *=* number of mice. WT, wild type; A-P, anteroposterior; M-L, medio-lateral; D-V, dorso-ventral; 2XRheo, two times the rheobase current injection; Max, maximal firing frequency; Mem, membrane intrinsic property; pcs, pieces. Source data

**Extended Data Fig. 1:**
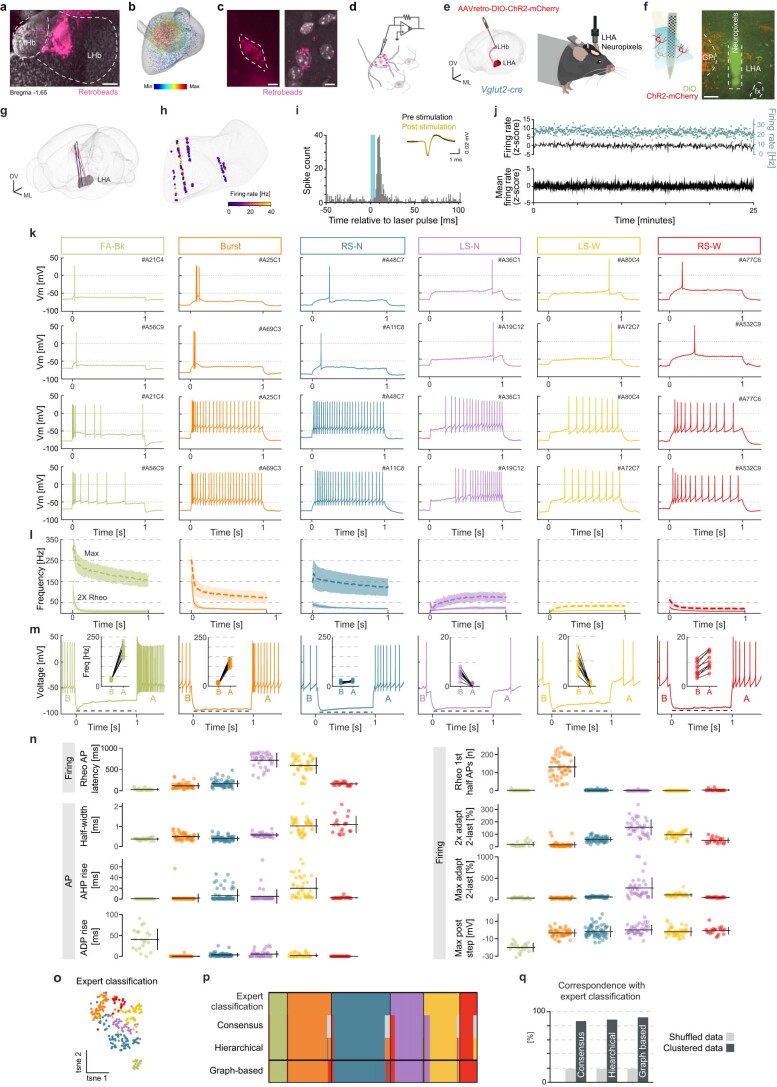
*In vivo* and *ex vivo* electrophysiology of LHA-LHb neurons. **(a)** Representative image of retrobeads injected into the LHb. **(b)** Volume-density distribution of retrobead labeled LHA-LHb neurons (n = 5883, N = 7 wt). **(c)** Representative images of retrobeads (pink) in retrogradely labeled LHA-LHb neurons. Left: epifluorescence (patch-clamp set-up), right: confocal. **(d)** Schematic of patch-clamp recordings of retrobead-labeled LHA-LHb neurons. **(e)** Experimental design for opto-tagging (Neuropixels) of LHA-LHb neurons in awake head-fixed mice. **(f)** Left: schematic illustration of Neuropixels probe dyed with DiO (green), LHA-LHb neurons expressing ChR2-mCherry (red), and light application (light blue). Right: image of DiO labeled probe track (green) and ChR2-mCherry expressing neurons (red). **(g)** 3D rendering of the tracked anatomical position of five Neuropixels probes in the LHA. **(h)** The spontaneous firing frequency (color coded) of recorded LHA-LHb units (n = 165 units; N = 5 Vglut2-cre mice). **(i)** Peri-stimulus histogram for an example light-activated (opto-tagged) LHA-LHb unit relative to light stimulus (blue). Inset: spontaneous (black) and light-evoked (yellow) spike waveforms. **(j)** Top: 25 min recording of an example opto-tagged, tonically active LHA-LHb unit. Turquoise: raw firing rate, black: z-scored firing rate. Bottom: average, z-scored firing rates of opto-tagged, tonically active LHA-LHb units (n = 9, N = 9 Vglut2-cre mice). **(k)** Example firing patterns (current clamp) of the six identified LHA-LHb neuron types. Upper two rows: response at rheobase; lower two rows: response at double rheobase. **(l)** Average of inter-spike interval (ISI) immediate frequencies from whole-cell firing patterns at 2X Rheo and at Max (mean ± SD). Same neurons as in Fig. 1b. **(m)** Example responses to hyperpolarizing current injections (n = 8 for each neuron type). Insert: firing frequency as the average of the 200 ms firing before (B) and after (A) hyperpolarization. Dashed horizontal line: hyperpolarization activated depolarization or “Sag-potential”. Same neurons as in Fig. 1b. **(n)** Hive plots of AP- and firing-related parameters for the six identified LHA-LHb neuron types (mean ± SD, n = 230 neurons, N = 46 mice). **(o)** t-SNE plot of LHA-LHb neuron types classified by electrophysiology. Color code: expert classification. **(p)** Comparison of neuronal identity by expert classification, hierarchical clustering, graph-based clustering, and consensus clustering. Gray: lack of agreement. Consensus clustering: the agreement between hierarchical and graph-based unbiased analyses. **(q)** Correspondence of the three clustering approaches (hierarchical clustering, graph-based clustering, and consensus clustering) and the randomly shuffled data of each clustering with the expert classification. n = number of neurons, N = number of animals. Abbreviations: Globus pallidus internus (GPi), fornix (fx), All data acquired in male mice. Scale bars: 100 μm (**a)**, 10 μm (**c**), 150 μm (**f**). Source data

**Extended Data Fig. 2:**
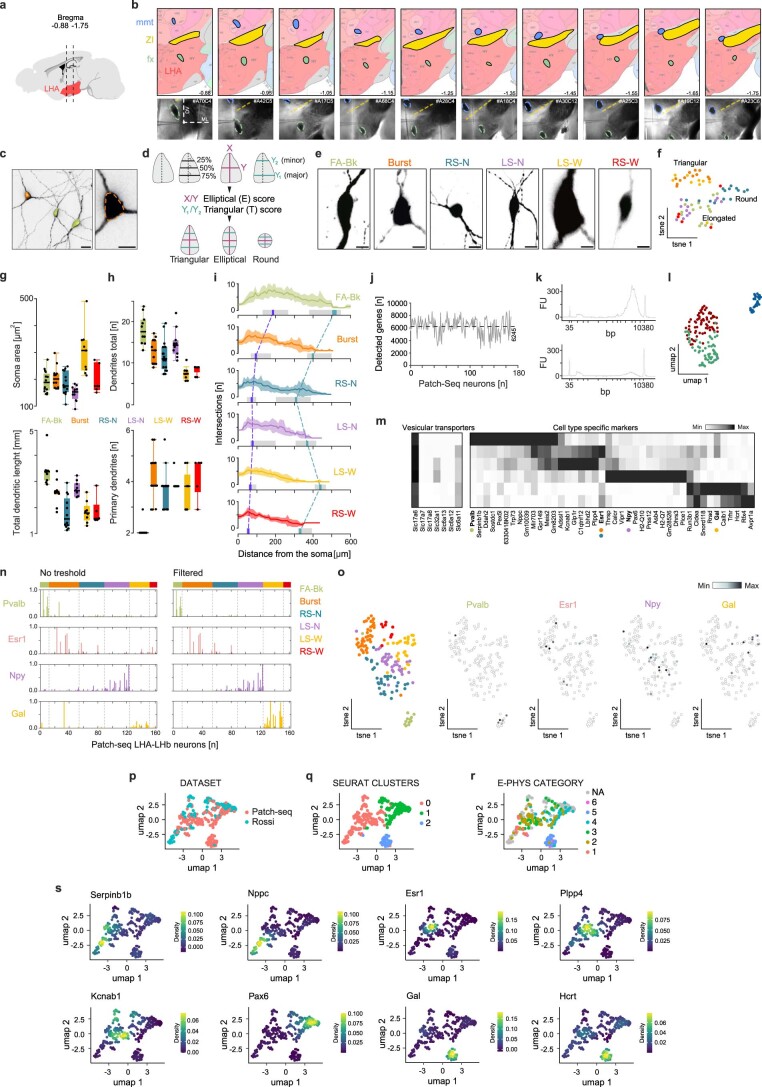
Anatomical, morphological, and genetic characterization of electrophysiologically recorded LHA-LHb neurons. **(a)** Left: AP range of tissue used for whole-cell patch-clamp recordings. **(b**) Right: landmarks in the vicinity of the LHA used for mapping of the AP origin of coronal sections used for whole-cell patch-clamp recordings of LHA-LHb neurons: mammillary tract (blue), fornix (green), zona incerta (yellow). Top: coronal anatomical plates (Allen Brain Atlas CCFv2) at different AP coordinates. Bottom: DIC image of a corresponding *ex vivo* brain slice and a recording electrode. The relative DV and ML position of mmt, fx, and ZI was used to identify the AP position of the brain slice. **(c)** Confocal image of three representative biocytin filled LHA-LHb neurons. Left: Pseudo-coloring of the soma. Right: the soma outlines (dashed line) were detected in the orthogonal projection of confocal z-stack. **(d)** Outline of the workflow for soma morphometry. The elliptical score (X/Y ratio, magenta lines) and the triangular score (Y1/Y2 ratio, turquoise lines) were used for classification of soma as triangular, elongated, or round, respectively. **(e)** Representative soma shapes (biocytin filled) of the six identified LHA-LHb neuron types. **(f)** t-SNE plot of the parameters established by soma morphometry. Color code: expert classification. FA-Bk: n = 16, Burst: n = 12, RS-N: n = 14, LS-N: n = 11, LS-W: n = 9, RS-W: n = 5 LHA-LHb neurons, N = 17 wt mice. **(g)** Quantification of the soma area (top, same neurons as in (**f**)) and total dendritic length (bottom, FA-Bk: n = 8, Burst: n = 10, RS-N: n = 12, LS-N: n = 8, LS-W: n = 8, RS-W: n = 5. N = 19 wt mice). **(h)** Quantification of the total number of dendrites (top) and the number of primary dendrites (bottom). FA-Bk: n = 10, Burst: n = 11, RS-N: n = 16, LS-N: n = 10, LS-W: n = 8, RS-W: n = 5. **(i)** Sholl analysis (number of intersections) of reconstructed neurons highlights the cell-type specific complexity of the dendritic trees of LHA-LHb neuron types, mean ± s.e.m. Violet vertical bar: median of the critical radius, turquoise vertical bar: median of the enclosing radius, gray horizontal bar: 95 percentile. Same neurons as in (**g**), bottom. **(j)** Number of detected genes in the Patch-seq LHA-LHb neurons (n = 163, same neurons as in Fig. 2b-d). Average: 6245 genes/neuron. **(k)** Representative examples of Agilent Bioanalyzer traces of cDNA amplicons of mRNA samples from single Patch-seq harvested LHA-LHb neurons. Top: successful sample (included in data), bottom: failed sample (discarded), ~10% of samples were discarded. **(l)** UMAP plot showing identity of neurons based on gene expression from Patch-seq, identifying three clusters of LHA-LHb neurons, n = 163, same neurons as in Fig. 2b-d, color code as in Fig. 2b, right. **(m)** Heat-map of marker genes in the electrophysiologically defined LHA-LHb neuron types (same as in Fig. 2d) before thresholding. Colored dots: genetic markers employed for subsequent cell-type specific targeting. **(n)** bar plot showing expression of selected marker genes before and after thresholding. **(o)** t-SNE plot for Patch-seq LHA-LHb neurons (n = 163 sequenced neurons, N = 46 wt mice, same neurons as in Figs. 1–2). Expression of single markers genes visualized in separate t-SNE plots. **(p-r)** UMAP plot showing Patch-seq data projected onto the Rossi dataset (LHA neurons projecting to LHb only), color coded by dataset (p), Seurat generated cluster using gene expression only (q) and cell types defined by electrophysiological properties of the Patch-seq dataset (r). **(s)** UMAP plots showing expression of LHA-LHb subtype candidate markers identified by Patch-seq on the merged dataset. All data acquired in male mice. For boxplots (**g**-**h**), data shown as median (center line), box (25th and 75th percentiles), whiskers (nonoutlier minimum and maximum) and outliers (>1.5 interquartile range). Scale bars: 50 μm (**a**, top), 25 μm (**a**, bottom left), 10 μm (**a**, bottom right), 10 μm (**c**), 10 μm (**e**). n = number of neurons, N = number of animals. Abbreviations: mammillary tract (mmt), zona incerta (ZI), fornix (fx), #A70C4 (along all S2B) codes for patched cell ID: A-(animal number) and C-(cell number), and Uniform Manifold Approximation and Projection (UMAP). Source data

To define the possible heterogeneity of LHA–LHb neurons, we visualized single LHA–LHb neurons and used ex vivo cell-attached and whole-cell patch-clamp recordings to probe the intrinsic properties of 230 LHA–LHb neurons in 46 mice (Fig. 1a and Extended Data Fig. 1a–d). First, we measured the spontaneous activity in cell-attached recording mode ex vivo and found that all recorded LHA–LHb neurons were spontaneously discharging action potentials (APs; Fig. 1b). We validated the tonic firing activity of LHA–LHb neurons in vivo using Neuropixels recordings of optotagged LHA–LHb in head-fixed awake mice (Extended Data Fig. 1e–j). Following cell-attached recordings, we proceeded to ex vivo whole-cell recordings and characterized a large number of intrinsic parameters to classify putative neuron subtypes. We found that the recorded LHA–LHb neurons could be classified according to their firing types (for example, bursting, regular spiking and fast spiking). We, therefore, performed an expert-based classification and named the different neuron subtypes according to their defining intrinsic properties as follows: (1) fast adapting with putative Bk-current type (FA-Bk), (2) bursting type (Burst), (3) regular spiking with narrow APs type (RS-N), (4) late-spiking with narrow APs type (LS-N), (5) late-spiking with wide APs type (LS-W), (6) regular spiking with wide AP type (RS-W; Fig. 1c, Extended Data Fig. 1k–q and Supplementary Table 1). The key intrinsic properties were segregated anatomically in the LHA, suggesting topographical organization of the neuron subtypes (Fig. 1d–f). Supporting the anatomical organization of LHA–LHb neuron types, we found a discrete topographical organization of soma localization along the anteroposterior axis, as well as distinct somatic and dendritic morphologies for each subtype (Fig. 1e–h, Extended Data Fig. 2a–i and Supplementary Tables 2 and 3).

To validate the expert-based classification, we performed an independent clustering of the electrophysiological parameters using hierarchical clustering and graph-based clustering with 20 PCA-selected parameters from each neuron (Fig. 1i–k and Extended Data Fig. 1o). We found that the two unsupervised clustering methods resulted in a similar classification of LHA–LHb neurons into six clusters (Fig. 1j,k). To test the agreement between clustering methods, the two unsupervised approaches were first summed into a consensus clustering, which we then compared to the expert-based classification. We found that 214/230 neurons were classified into the same six neuron types independent of the approach used (Fig. 1l and Extended Data Fig. 1p,q). In summary, we uncovered a surprising diversity in the glutamatergic LHA–LHb pathway, establishing six discrete neuron types with characteristic tonic frequencies and intrinsic electrophysiological features.

## Molecular diversity of LHA–LHb neurons

**Fig. 2:**
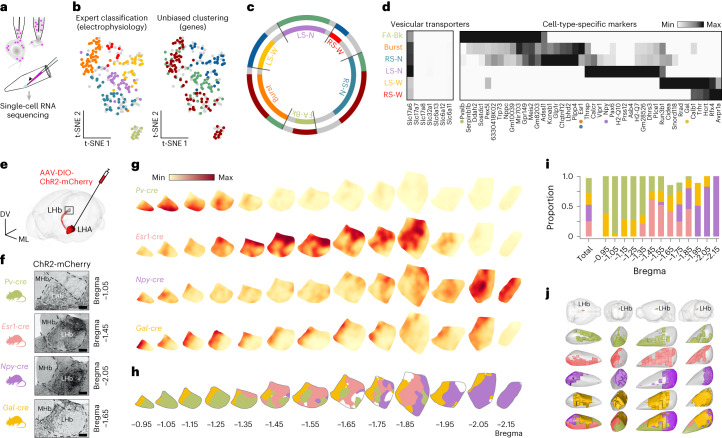
Discrete organization of genetically targeted LHA–LHb pathways. **a**, Schematic of Patch-seq showing somatic harvesting of retrobead-labeled LHA–LHb neurons. **b**, t-SNE plots of all recorded LHA–LHb neurons (*n*_neuron_ = 230, *n*_mice_ = 46 WT, same neurons as in Fig. 1). The cell type identify of neurons collected for Patch-seq are color coded based on electrophysiology (expert classification; left), or gene expression (unbiased clustering; right). *n* = 163 collected neurons; FA-Bk, *n*_neuron_ = 12; Burst, *n*_neuron_ = 42; RS-N, *n*_neuron_ = 35; LS-N, *n*_neuron_ = 35; LS-W, *n*_neuron_ = 28; RS-W, *n*_neuron_ = 11 and *n*_mice_ = 46 WT. Gray neurons, recorded but not collected. **c**, Comparison of electrophysiological (expert classification as in **b**, left) versus gene expression classification (unbiased clustering as in **b**, right) of LHA–LHb neurons (colored as in **b**). **d**, Heatmap of genes with differential expression in the electrophysiologically defined LHA–LHb neuron types (right). Expression of vesicular transporters in the LHA–LHb neuron types (left). Colored dots represent genetic markers employed for subsequent cell-type-specific targeting. **e**, Experimental strategy for anterograde labeling of LHA–LHb axon terminals. **f**, Representative images of virally labeled LHA–LHb axon terminals in the mouse *cre* lines used for targeting specific LHA–LHb pathways (*Pv-cre*, *Esr1-cre*, *Npy-cre* and *Gal-cre* mice, respectively). Brain section with peak terminal density is shown. **g**, Heatmaps of the axon terminal density in the LHb for the four genetically targeted LHA–LHb pathways. **h**, Visualization of the topographical organization of the pathway-specific projection fields in the LHb. Colors as in **g**, white is not assigned to a specific pathway (Methods). **i**, Proportion of the LHb area targeted by the distinct LHA–LHb pathways, plotted along the A-P axis. Left bar, cumulative targeting of LHb by the four LHA–LHb pathways. **j**, Three-dimensional reconstructions (four different orientations) of the LHb projection fields of the four LHA–LHb pathways. *n*_neuron_ = number of neurons, *n*_mice_ = number of mice. All data were acquired in male mice. Scale bar, 100 μm (**f**). See also Extended Data Figs. 3 and 4. MHb, medial habenula.

We then asked whether the electrophysiological properties of the six LHA–LHb neuron types could also be captured by other modalities, for example, their gene expression pattern. We therefore used Patch-seq^21^ to obtain a combined view of intrinsic physiology, morphology, position and gene expression pattern for the whole-cell-recorded neurons (Fig. 2a). We performed Patch-seq on 163 neurons (46 mice), resulting in ~6,200 detected genes per cell on average (Extended Data Fig. 2j,k). We used unbiased clustering of the single-cell RNA profiles to overlay the extracted molecular identity onto the expert-based electrophysiological classification (Fig. 2b). Clustering based on gene expression data alone was not sufficient to identify the putative six LHA–LHb types, and the three molecular clusters consisted of multiple electrophysiological types (Fig. 2c and Extended Data Fig. 2l). We, therefore, used the electrophysiological data to identify cell-type-specific markers for the six neuron types. We found that the LHA–LHb neurons as expected expressed the glutamatergic marker Vglut2 (gene name, *Slc17a6*) and could be separated based on the expression of a palette of molecular markers—FA-Bk neurons were Pv^+^/Nppc^+^, Burst neurons were Esr1^+^/Plpp4^+^, RS-N neurons were Esr1^+^/Glpr1^+^, LS-N neurons were Npy^+^/Pax6^+^, LS-W neurons were Gal^+^/Hcrt^+^ and RS-W neurons were Avpr1a^+^/Hcrt^+^ (Fig. 2d and Extended Data Fig. 2m–o). To support the putative cell type markers, we projected the Patch-seq data onto an available dataset on LHA–LHb-projecting neurons^16^ and found good agreement in cluster-enriched expression patterns (Extended Data Fig. 2p–s). In conclusion, the multimodal classification of Vglut2^+^ LHA–LHb neurons based on electrophysiological, morphological and molecular definitions together established six discrete neuron subtypes.

## Genetic targeting of LHA–LHb neuron types and neuroanatomical organization

**Extended Data Fig. 3:**
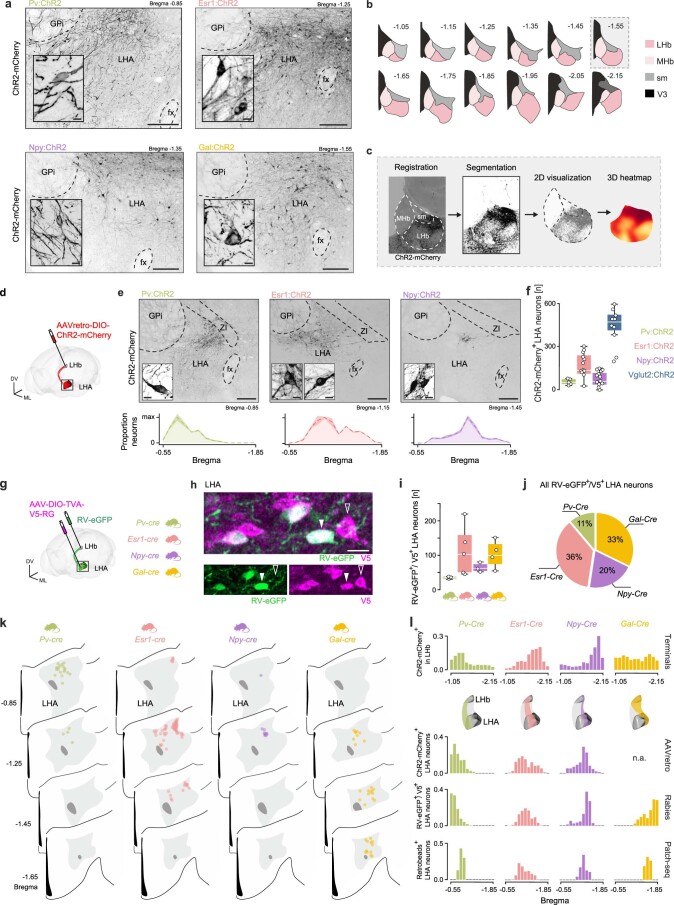
Genetic targeting of LHA-LHb pathways. **(a**) Representative confocal images of cre-dependent anterograde labeling of LHA-LHb neurons in Pv-cre, Esr1-cre, Npy-cre, and Gal-cre mice, respectively. Inserts: representative examples of cell-type specific soma morphologies. **(b)** Anatomical plates (Allen Brain Atlas CCFv2) of the LHb and neighboring structures along the AP range. **(c)** Outline of the workflow for anatomical mapping of axon terminals. **(d)** Experimental design for cell-type specific retrograde labeling of LHA-LHb neurons. **(e)** Top: representative confocal images of retrogradely labeled neurons in the LHA. Insert: representative examples of cell-type specific soma morphologies. Bottom: quantification of the labeled soma along the AP axis (mean ± s.e.m; Pv:ChR2: N = 5, Esr1:ChR2: N = 12, Npy:ChR2: N = 13). **(f)** Quantification of retrogradely labeled LHA-LHb neurons (same mice as in (**e**) + Vglut2:ChR2: N = 10. **(g)** Experimental design for rabies tracing. The helper virus AAV-DIO-TVA-V5-RG was injected in the LHA, and the EnvA-coated rabies virus (RV-eGFP) in the LHb. **(h)** Representative confocal image of LHA-LHb neurons co-expressing RV-eGFP (green) and V5 (magenta; Esr1-cre mouse). Arrow head: RV-eGFP+/V5+ neuron (quantified), empty arrow head: RV-eGFP-/V5+ neuron (not quantified). **(i, j)** Quantification of RV-eGFP+/V5+ LHA-LHb neurons Pv-cre, Gal-cre: N = 4, Esr1-cre: N = 5, Npy-cre: N = 3. (**i**) total number, (**j)** the proportion (average) RV-eGFP+/V5+ LHA neurons in each genotype, of all RV-eGFP+/V5+ labeled LHA neurons detected across the genotypes. Pv-cre, Gal-cre: N = 4; Npy-cre: N = 3; Esr1-cre: N = 5 mice. **(k)** Anatomical plates (Allen Brain Atlas CCFv2) of the LHA along the AP axis with the position of detected RV-eGFP+/V5+ LHA-LHb neurons. One dot = one neuron. Each plate depicts neurons detected in two consecutive brain sections (50 μm) from a representative mouse. **(l)** Topography of the projection field and the soma location of the LHA-LHb pathways along the AP axis. From top to bottom: bar plots with the proportion axon terminals in the LHb (anterograde viral strategy, Pv-cre: N = 3, Esr1-cre: N = 5, Npy-cre: N = 4, Gal-cre: N = 4 mice); soma in the LHA (retrograde viral strategy, Pv-cre: N = 5, Esr1-cre: N = 12, Npy-cre: N = 13); soma in the LHA (rabies tracing, Pv-cre: N = 4, Esr1-cre: N = 5, Npy-cre: N = 3, Gal-cre: N = 4); neurons in the LHA (Patch-seq, FA-Bk: n = 12, Burst + RS-N: n = 77, LS-N: n = 35, LS-W: n = 28). N = number of animals. Abbreviations: Globus pallidus internal (GPi), fornix (fx), medial habenula (MHb) and zona incerta (ZI), medial habenula (MHb), stria medullaris (sm), and third ventricle (V3). For boxplots (**f, i**), data shown as median (center line), box (25th and 75th percentiles), whiskers (nonoutlier minimum and maximum) and outliers (>1.5 interquartile range). Scale bars: 200 μm (**a, e)**, inset 10 μm (**a, e**), 10 μm (**h**). Source data

**Extended Data Fig. 4:**
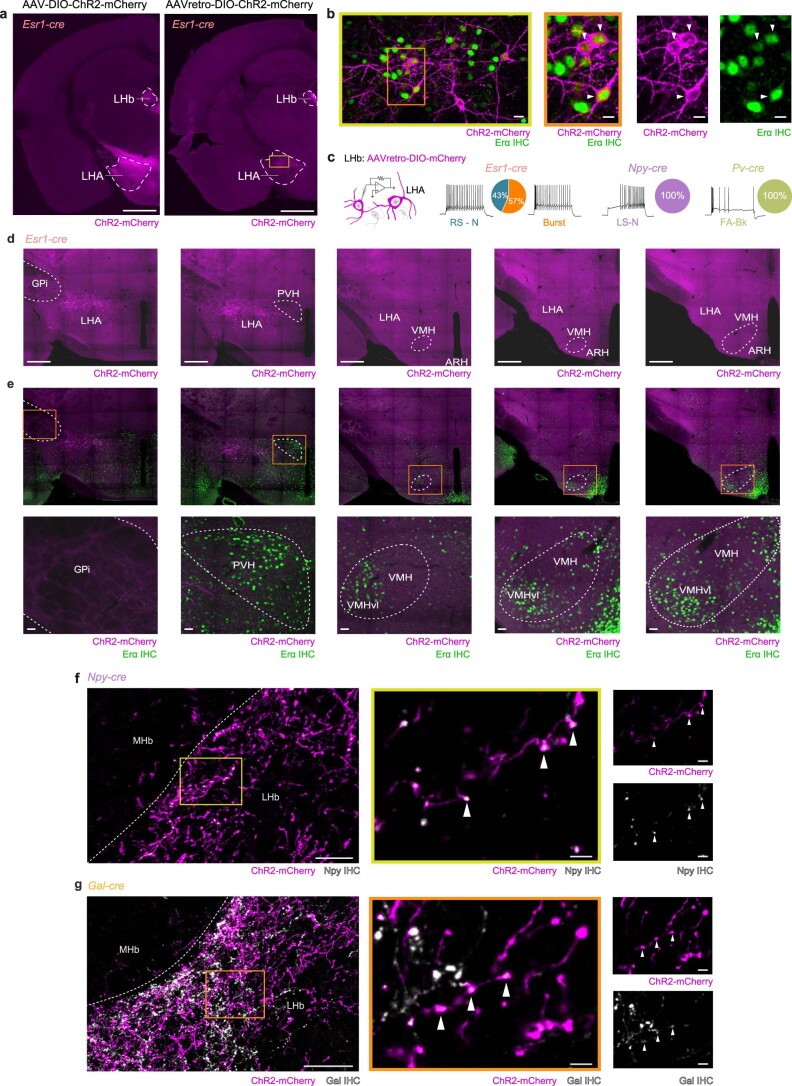
Characterization of distinct LHA-LHb neuron types. **(a**) Anterograde (left) and retrograde (right) viral labeling of the Esr1+ LHA-LHb pathway in the Esr1-cre mice. **(b)** ESR1 protein expression (green) was confirmed by IHC in retrogradely labeled LHA-LHb neurons (magenta) in Esr1-cre mice. Orange box: location of right panel. **(c)** Retrograde viral labeling of Esr1+ LHA-LHb neurons with consecutive whole-cell patch-clamp characterization of labeled neurons exclusively identified RS-N (n = 10) and Burst (n = 13) type neurons, corroborating the detection of Esr1 in the Patch-seq transcriptome of these neuron types. N = 4 Esr1-cre mice. Retrograde viral labeling of the LHA-LHb pathway in Npy-cre and Pv-cre mice, respectively, with consecutive whole-cell patch-clamp characterization of labeled neurons exclusively identified LS-N type LHA-LHb neurons (n = 19) in Npy-cre mice (N = 3), and FA-Bk type LHA-LHb neurons (n = 12) in PV-cre mice (N = 3), confirming the validity of the experimental strategy for cell type specific targeting of LHA-LHb pathways. **(d)** Representative confocal images along the AP axis (left to right) of the hypothalamic area with retrograde viral labeling (magenta) of the LHA-LHb pathway in Esr1-cre mice. **(e)** Top: same images as in (**d**), here with detection of ESRα protein expression (IHC; green). Orange box: location of panel in bottom row. Bottom: close-up of box in top. **(f-g)** Left: representative confocal images of virally labeled (magenta) LHA-LHb axon terminals in the LHb of a Npy-cre mouse (**f**) and a Gal-cre mouse (**g**), confirming expression of the neuropeptide Y (white) in the LHA-LHb axon terminals in Npy-cre mice, and expression of galanin (white) in the LHA-LHb axon terminals in Gal-cre mice. Yellow/orange box: location of right panel. Right: close-up of box in left. All data acquired in male mice. Scale bars: 1 mm (**a**), 10 μm (**b**, **f**, left), 200 μm (**d**), 50 μm (**e**, bottom), 20 μm (**f**, left), 2 μm (**f**, center and right), 20 μm (**g**, left), 2 μm (**g**, center and right). n = number of neurons, N = number of animals. Abbreviations: Globus pallidus internal (GPi), paraventricular hypothalamic nucleus (PVH), ventromedial hypothalamic nucleus (VMH), ventromedial hypothalamic nucleus, ventrolateral part (VMHvl).

Based on the molecular profile of the LHA–LHb neuron types revealed by Patch-seq, we used mice with cell-type-specific Cre recombinase expression to target the putative neuron types. We used *Pv-cre*, *Esr1-cre*, *Npy-cre* and *Gal-cre* mice to target the main LHA–LHb neuron types, and injected Cre-dependent adeno-associated viruses (AAV-DIO) into the LHA. We found that this strategy resulted in the genetic labeling of the distinct neuron types and their projections to the LHb (Fig. 2e,f and Extended Data Fig. 3). To validate the cell-type-specific labeling strategy, we first established the specificity of our genetic targeting in *Esr1-cre* mice by confirming the presence of Esr1 protein in LHA–LHb neurons after Cre-dependent viral labeling (Extended Data Fig. 4a,b). In addition, we used whole-cell recordings to confirm that the retrograde cell-type-specific viral targeting approach labeled in *Esr1-cre* mice specifically the Burst and the RS-N neuron types, in *Npy-cre* mice the LS-N neurons type, as well as in *Pv-cre* mice the FA-BK LHA neuron type (Extended Data Fig. 4c). We also confirmed that retrograde targeting in *Esr1-cre* mice did not label neighboring Esr1^+^ nuclei (for example, VMH), and that axons from Npy^+^ and Gal^+^ LHA–LHb neurons expressed the corresponding peptide (Extended Data Fig. 4d–g).

Taking advantage of the cell-type specific labeling of the different LHA–LHb neuron types, we aimed to further determine the topographical organization of neuron types and map their projection pattern. Using two different retrograde viral labeling approaches, we mapped the number and location of genetically labeled LHA–LHb subtypes. In summary, we confirmed that LHA–LHb subtypes were discretely organized in the anteroposterior axis, and that the Esr1^+^ and Gal^+^ subtypes were comparable in population size, whereas Pv^+^ and Npy^+^ subtypes were a smaller population (Extended Data Fig. 3d–l). Notably, we found a specific organization of axon terminals for each LHA–LHb pathway, where axon terminals from Pv^+^ LHA–LHb neurons showed preferential targeting of anterior LHb domains, whereas the Esr1^+^ LHA–LHb neurons targeted the intermediate LHb domain with inputs to specific LHb subregions (for example, avoiding the oval-LHbLO and the parvocellular-LHbMPc subnucleus of the LHb; Fig. 2f–h and Extended Data Fig. 3b,c). In contrast, the Npy^+^ LHA–LHb neurons targeted the posterior LHb domains, whereas Gal^+^ LHA–LHb neurons targeted a medial LHb domain along the entire anteroposterior axis. Notably, the different LHA–LHb pathways targeted comparable cumulative areas, but that were organized topographically across the LHb (Fig. 2i,j). Together, the cell-type specific targeting of the LHA–LHb neuron subtypes showed that the molecular and physiological profile also reflected a specialized neuroanatomical organization.

## Specialized role of LHA–LHb neuron types in behavior

**Fig. 3:**
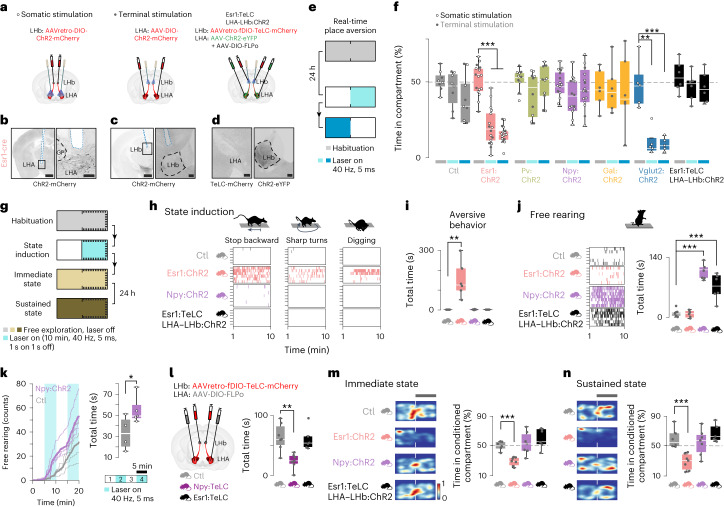
The Esr1^+^ LHA–LHb pathway drives aversion. **a**, Experimental strategy for somatic optogenetic manipulation of LHA–LHb neurons (left), optogenetic manipulation of LHA–LHb axon terminals in the LHb (middle) and optogenetic manipulation of the axon terminals in the LHb of the entire LHA–LHb pathway in combination with TeLC silencing of the Esr1^+^ LHA–LHb neurons (right). **b**–**d**, Representative images of the optic fiber path and/or viral expression for the three experimental strategies. **b**, ChR2–mCherry expression and somatic optogenetic manipulation of Esr1^+^ LHA–LHb neurons. Black box, location of the right panel. **c**, ChR2–mCherry expression and optogenetic stimulation of Esr1^+^ LHA–LHb axon terminals in the LHb. Black box, location of the right panel. **d**, Nuclear TeLC-mCherry expression in Esr1^+^ LHA–LHb neurons in the LHA (left), and ChR2-eYFP expression in LHA–LHb terminals in the LHb (right). **e**, Schematic outline of the real-time place aversion test (rtPA). Habituation of 10 min was 24 h later followed by optogenetic stimulation paired to the right compartment (ON, light blue, 10 min) and an immediate switch of the optogenetic stimulation to the left compartment (ON SWITCH, blue, 10 min). **f**, Behavior in the rtPA. Optogenetic activation of the Esr1^+^ LHA–LHb and the VGlut2^+^ LHA–LHb pathway, respectively, significantly reduced the time spent in the compartment paired with optogenetic stimulation (Esr1:ChR2 habituation versus ON, *P* = 6.20 × 10^**−**9^; Esr1:ChR2 habituation versus ON SWITCH, *P* = 5.72 × 10^**−**14^; Vglut2:ChR2 habituation versus ON, *P* = 0.0017, Vglut2:ChR2 habituation versus ON SWITCH, *P* = 7.07 × 10^**−**4^, two-sided unpaired *t*-test). Horizontal bars: light blue, ON; blue, ON SWITCH; gray, right compartment during habitation. Empty circles represent somatic stimulation and gray dots axon terminal stimulation. Ctl, *n*_mice_ = 8; Esr1:ChR2, *n*_mice_ = 16; Pv:ChR2, *n*_mice_ = 10; Npy:ChR2, *n*_mice_ = 15; Gal:ChR2, *n*_mice_ = 6; Vglut2:ChR2, *n*_mice_ = 5; Esr1:TeLC LHA–LHb:ChR2, *n*_mice_ = 6. **g**, Schematic outline of the strategy for induction of an immediate (beige) and sustained aversive state (brown). State induction (light blue), 10 min optogenetic stimulation of LHA–LHb axon terminals in the LHb. **h**,**i** Scoring of aversive behaviors (stop-backward movement, sharp turns and digging) during state induction, one color coded vertical bar = 1 s in a specific aversive behavior. Ctl, Esr1:ChR2 and Esr1:TeLC LHA–LHb:ChR2 mice—*n*_mice_ = 6, each, and Npy:ChR2—*n*_mice_ = 7, same animals in **i**, **j** and **m**,**n**. **i**, The optogenetic stimulation of Esr1^+^ LHA–LHb axon terminals significantly increased the total time spent in aversive behaviors during state induction (Esr1:ChR2 versus Ctl, *P* = 0.0022, two-sided unpaired *t*-test). Same animals in **j**. **j**, Scoring of free rearing during state induction, one colored coded vertical bar = 1 s free rearing (left). Optogenetic stimulation of Npy^+^ LHA–LHb axon terminals significantly increased the time spent free rearing (Npy:ChR2 versus Ctl, *P* = 1.17 × 10^**−**7^, unpaired *t*-test). The same effect was seen in response to axon terminal stimulation of the compound LHA–LHb pathway in combination with TeLC silencing of the Esr1^+^ LHA–LHb neurons (Esr1:TeLC LHA–LHb:ChR2 versus Ctl, *P* = 9.39 × 10^**−**4^, two-sided unpaired *t*-test; right). Same animals in **h**. **k**, Free rearing in the open field. Somatic activation of Npy^+^ LHA–LHb neurons significantly increased the total time spent free rearing (right; Npy:ChR2 (*n*_mice_ = 5) versus Ctl (*n*_mice_ = 4), *P* = 0.0464, unpaired *t*-test). Cumulative number of free-rearing events; thick line, mean (left). **l**, Experimental strategy for TeLC mediated silencing of Npy^+^ LHA–LHb and Esr1^+^ LHA–LHb neurons, respectively (left). TeLC silencing of Npy^+^ LHA–LHb neurons significantly decreased the total time spent free rearing in the open field (right; Npy:TeLC (*n*_mice_ = 7) versus Ctl (*n*_mice_ = 7), *P* = 0.0016, unpaired *t*-test). Esr1:TeLC, *n*_mice_ = 6 mice. **m**,**n**, Free exploration directly after (**j**) and 24 h after (**k**) the state induction. Optogenetic stimulation of Esr1^+^ LHA–LHb axon terminals significantly increased conditioned place aversion (right; immediate state, Esr1:ChR2 versus Ctl mice, *P* = 9.56 × 10^**−**5^; sustained state, Esr1:ChR2 versus Ctl mice, *P* = 7.58 × 10^**−**4^, two-sided unpaired *t*-test). Representative heatmaps of locomotion (left). **m**, Ctl *n*_mice_ = 6 and Esr1:ChR2, Npy:ChR2 and Esr1:TeLC LHA–LHb:ChR2, *n*_mice_ = 7 (each). **n**, Ctl and Esr1:TeLC LHA–LHb:ChR2, *n*_mice_ = 6 (each); Esr1:ChR2 and Npy:ChR2, *n*_mice_ = 7 (each). *n*_mice_ = number of mice. All data were acquired in male mice. For boxplots **f** and **i**–**n**, data are shown as median (center line), box (25th and 75th percentiles), whiskers (nonoutlier minimum and maximum) and outliers (>1.5 interquartile range). Scale bars, 1 mm; boxes, 100 μm (**b**–**d**). *P < 0.05, **P < 0.01, ***P < 0.001. See also Extended Data Fig. 5 and 6, Supplementary Videos 1 and 2 and Supplementary Table 4. Source data

**Extended Data Fig. 5:**
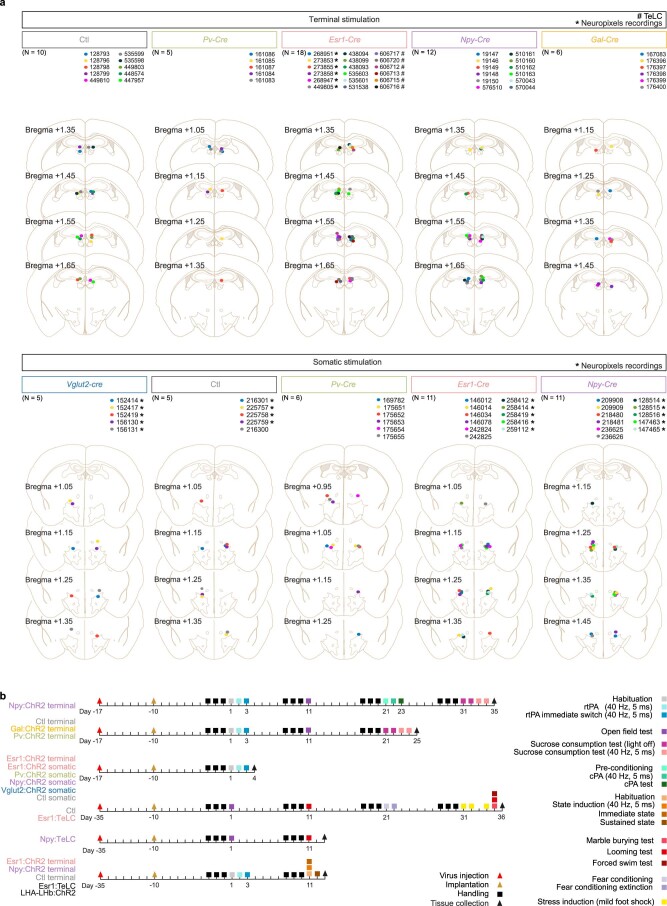
Confirmation of the position of optical fibers, and overview of experimental timelines. **(a)** The location of the tip of the optical fibers across the experiments, visualized across the AP axis of coronal anatomical plates (Allen Brain Atlas CCFv2). Top: position of the tip of optical fibers in experiments with optogenetic stimulation of LHA-LHb axon terminals in the LHb. Bottom: position of the tip of optical fibers in experiments with somatic optogenetic stimulation of LHA-LHb neurons in the LHA. Colored dots: individual mice and their respective bilateral optical fiber location. *) mice subjected to Neuropixels recordings, #) mice subjected to TeLC silencing. **(b)** Schematic overview of the experimental timeline for the different cohorts of mice. N = number of mice.

**Extended Data Fig. 6:**
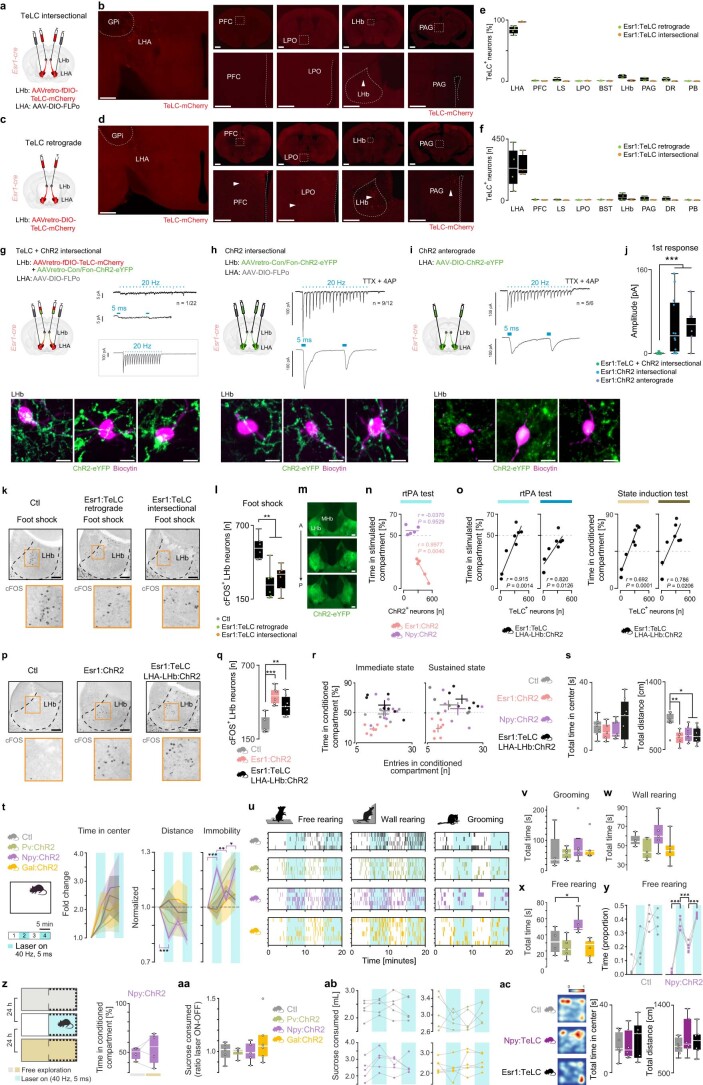
Validation of the TeLC silencing approaches, and optogenetic manipulation or TeLC silencing of LHA-LHb pathways during behavior. **(a)** Experimental design of the intersectional approach for TeLC silencing of LHA-LHb neurons. (**b**) Representative confocal images of TeLC labeled LHb projecting neurons (red) in the LHA and potential off-target brain regions in a Esr1-cre mouse for the TeLC intersectional approach. (**c**) Experimental design of the retrograde approach for TeLC silencing of Esr1+ LHA-LHb neurons. (**d**) Representative confocal images of TeLC labeled neuron (red) LHb projecting neurons (red) in the LHA and potential off-target brain regions in a Esr1-cre mouse for the TeLC retrograde approach. **(e-f)** Quantification of LHb projecting neurons expressing TeLC-mCherry in the LHA and potential off-target regions in Esr1-cre mice. (**e**) The distribution of TeLC-mCherry+ neurons in different brain regions, plotted as the percentage of the total number of TeLC-mCherry+ neurons in individual mice. (**f**) The number of TeLC-mCherry+ neurons in different brain regions. Green dots: mice with TeLC retrograde approach (N = 4 mice), orange dots: mice with TeLC intersectional approach (N = 3 mice). **(g)** Electrophysiological *ex vivo* validation of the silencing of Esr1+ LHA-LHb neurons. Left: experimental design of the intersectional viral approach for combined TeLC silencing and ChR2-expression in Esr1+ LHA-LHb neurons. Right: whole-cell recordings of LHb neurons with light delivery (1 s, 5 ms pulses, 20 Hz) in the LHb validated the absence of light-evoked synaptic responses upon TeLC silencing of Esr1+ LHA-LHb neurons. Top: representative light-evoked response in a LHb neuron (synaptic response in 1/22 LHb neuron). Middle: close-up of trace in top, showing the two first responses to light stimulation. Box: Representative light-evoked response of a TeLC + /ChR2+ neuron in the LHA. Bottom images: representative confocal images of whole-cell recorded biocytin (magenta) filled LHb neurons showing ChR2-eYPF+ (green) axon terminals from Esr1+ LHA-LHb neurons. **(h)** Left: experimental design of the intersectional viral approach for expression of ChR2 in Esr1+ LHA-LHb axon terminals. Right: whole-cell recordings of LHb neurons concurrent with light application (1 s, 5 ms pulses, 20 Hz) in the LHb confirmed reliable synaptic responses in LHb neurons, validating the experimental strategy. Top: representative light-evoked response in a LHb neuron (synaptic response in 9/12 LHb neurons). Bottom: close-up of trace in top, showing the two first responses to light stimulation. Bottom images: representative confocal images of whole-cell recorded biocytin (magenta) filled LHb neurons showing ChR2-eYPF+ (green) axon terminals from Esr1+ LHA-LHb neurons. **(i)** Left: experimental design of the anterograde viral approach for expression of ChR2 in Esr1+ LHA-LHb axon terminals. Right: whole-cell recordings of LHb neurons concurrent with light application (1 s, 5 ms pulses, 20 Hz) in the LHb confirmed reliable synaptic responses in LHb neurons, validating the experimental strategy. Top: representative light-evoked response in a LHb neuron (synaptic response in 5/6 LHb neurons). Bottom: close-up of trace in top, showing the two first responses to light stimulation. Bottom images: representative confocal images of whole-cell recorded biocytin (magenta) filled LHb neurons showing ChR2-eYPF+ (green) axon terminals from Esr1+ LHA-LHb neurons. **(j)** Average amplitude of the first light-evoked synaptic response for all the whole-cell recorded LHb neurons in (**g**-**i)**. TeLC + ChR2 intersectional vs ChR2 intersectional, p = 8.2E-5; TeLC ChR2 intersectional vs ChR2 anterograde, p = 4.1E-7, two-sided unpaired *t*-test. Colored dots: viral approach employed. Green dots: Esr1:TeLC + ChR2 intersectional: n = 22, blue dots: Esr1:ChR2 intersectional: n = 12, gray dots: Esr1:ChR2 anterograde: n = 6. **(k-l)** TeLC expression significantly reduced the expression of cFOS in response to foot shock. Representative confocal images (**k**) and quantification (**l**) of cFOS+ neurons in the LHb in response to foot shock in control mice and mice with TeLC silencing of Esr1+ LHA-LHb neurons, respectively. Two different approaches were used for TeLC mediated silencing. (**l**) Ctl vs Esr1:TeLC retrograde, p = 0.0014; Ctl vs Esr1:TeLC intersectional, p = 0.0016, two-sided unpaired *t*-test, N = 6 for each the three group of mice. **(m)** Representative confocal images of the LHb region along the AP axis, showing ChR2-eYFP+ (green) LHA-LHb axon terminals in a Esr1-cre mouse subjected to the Esr1:TeLC + entire LHA-LHb pathway ChR2 stimulation. **(n)** The relationship (two-sided Pearson correlation coefficient) between the number of ChR2+ neurons and the time spent (%) in the stimulated compartment in the rtPA. (**o**) The relationship (two-sided Pearson correlation coefficient) between the number of TeLC+ neurons and the time spent (%) in the stimulated compartment in the rtPA (left), or the conditioned compartment in the cPA (right). **(p-q)** Representative confocal images (**p**) and quantification (**q**) of cFOS+ neurons in the LHb in response to optogenetic stimulation of Esr1+ LHA-LHb axons terminal in mice with TeLC silencing (Esr1:TeLC LHA-LHb ChR2 mice) or without TeLC silencing (Esr1:ChR2 mice) of Esr1+ LHA-LHb neurons. Ctl vs Esr1:ChR2, p = 4.76E-4; Ctl vs Esr1:TeLC LHA-LHb ChR2, p = 0.0086, two-sided unpaired *t*-test. Ctl: N = 5, Esr1:ChR2: N = 6, Esr1:TeLC LHA-LHb ChR2: N = 7 mice. **(r)** Optogenetic activation of Esr1+ LHA-LHb axon terminals (Esr:ChR2 mice) reduces both the time spent and the number of entries into the conditioned compartment. Ctl, Esr1:ChR2, Esr1:TeLC LHA-LHb ChR2: N = 6 mice, Npy:ChR2: N = 7 mice, same mice as in Fig. 3j-k. **(s)** Open field. Continuous optogenetic activation (10 min, 40 Hz, 5 ms, 1 s on – 1 s off) of LHA-LHb axon terminals significantly decreased the distance traveled in Esr1:ChR2, Npy:ChR2, and Esr1:TeLC LHA-LHb ChR2 mice compared to Ctl mice (right, Ctl vs Esr1:ChR2, p = 9.1E-3; Ctl vs Npy:ChR2, p = 0.0172; Ctl vs Esr1:TeLC LHA-LHb ChR2, p = 0.0114, two-sided unpaired *t*-test), while the time spent in the center was not affected (left). Ctl, Esr1:ChR2, Esr1:TeLC LHA-LHb ChR2: N = 6 mice, Npy:ChR2: N = 7 mice, same mice as in (**r**) and in Fig. 3j-k. **(t)** Open field. Block wise optogenetic activation (20 min total, 4 ×5 min blocks, 40 Hz, 5 ms) of the axon terminals of the Pv+, Npy+, or Gal+ LHA-LHb pathway, respectively, did not affect the time spent in the center of an open field. The time spent in the center in each block, plotted as fold change compared to block 1 (mean ± s.e.m, two-sided t-test pairwise comparison of each block to the previous). Optogenetic activation of the axon terminals of the Npy+ LHA-LHb pathway significantly decreased the distance traveled in the open field (Npy:ChR2 block 1 vs block 2, p < 0.001, two-sided t-test pairwise comparison of each block to the previous). This effect was also apparent as significantly increased immobility (block 2 vs1, p < 0.001; block 3 vs 2, p = 0.0043; block 4 vs 3, p = 0.038, two-sided t-test pairwise comparison of each block to the previous). No significant effects on locomotion were observed in responses to activation of the Pv+ and Gal+ LHA-LHb pathways, respectively (two-sided t-test pairwise comparison of each block to the previous). Ctl: N = 4 mice, Pv:ChR2 and Npy:ChR2: N = 5 mice, Gal:ChR2: N = 6 mice. **(u-x)** Scoring of free rearing, wall rearing, and grooming in the open field in response to block wise optogenetic activation of the axon terminals of the Pv+, Npy+, or Gal+ LHA-LHb pathway, respectively. Ctl: N = 4 mice, Pv:ChR2 and Npy:ChR2: N = 5 mice, Gal:ChR2: N = 6 mice, same animals as in (**t**). 1 colored coded vertical bar = 1 s in a specific behavior. (**x**) Optogenetic activation of the axon terminals of the Npy+ LHA-LHb pathway significantly increased the total time spent free rearing (Npy:ChR2 vs Ctl, p = 0.0493, two-sided unpaired *t*-test. Ctl: N = 4 mice, Npy:ChR2: N = 5 mice, same mice as in Fig. 3k. **(y)** Quantification of proportion time free rearing in blocks with (blue) and without (white) light activation, respectively. Optogenetic activation of the axon terminals of the Npy+ LHA-LHb pathway significantly increased the proportion time spent free rearing (block 2 vs 1, p = 2.55E-7; block 3 vs 2, p = 1.91E-4; block 4 vs 3, p = 8.93E-7, two-sided t-test pairwise comparison of each block to the previous). Same mice as in Fig. 3k. **(z)** Left: schematic outline of the experimental design for conditioned place aversion. Optogenetic activation (10 min) of the axon terminals of the Npy+ LHA-LHb pathway did not induce avoidance of the conditioned compartment (right, two-sided unpaired *t*-test). Gray horizontal bar: before conditioning, gold horizontal bar: after conditioning. N = 4 Npy:ChR2 mice. **(aa-ab)** Sucrose consumption test, with block wise optogenetic activation (as in (**t**)). Optogenetic activation of the axon terminals of the Pv+, Npy+, or Gal+ LHA-LHb pathway, respectively, did not alter sucrose consumption (two-sided unpaired *t*-test). (**aa**) The ratio of the sucrose consumed in blocks with (ON) vs blocks without (OFF) light application. (**ab**) Sucrose consumed in the individual blocks. Ctl, Pv:ChR2, Npy:ChR2, Gal:ChR2: N = 5 mice. **(ac)** TeLC silencing of Npy+ LHA-LHb neurons or Esr1+ LHA-LHb neurons did not alter behavior in the open field (two-sided unpaired *t*-test. TeLC intersectional approach, see panel (**a**)). Left: heatmaps of example locomotion (10 min). Right: quantification of total time in center, and total distance, respectively. Ctl: N = 7, Npy:TeLC, Esr1:TeLC: N = 6 mice. n = number of neurons, N = number of animals. Abbreviations: real-time place aversion (rtPA), conditioned place aversion (cPA). All data acquired in male mice. For boxplots (**e-f**, **j**, **l**, **q**, **s**, **v-x**, **z-aa**, **ac**), data shown as median (center line), box (25th and 75th percentiles), whiskers (nonoutlier minimum and maximum) and outliers (>1.5 interquartile range). Scale bars: 1 mm (**b**, top, **d**, top), 500 μm (**b**, left, **d** left), 200 μm (**b**, bottom, **d** bottom), 100 μm (**k**, **m**, **o**), 10 μm (**g**-**i**), * *p* < 0.05, **p < 0.01, ***p < 0.001. Source data

To address whether the observed heterogeneity in the physiology and organization of LHA–LHb neuron types reflects functional specialization, we applied different strategies to first probe the contribution of each pathway to aversion. Based on candidate markers from the Patch-seq data, we used *Pv-cre*, *Esr1-cre*, *Npy-cre* and *Gal-cre* mice to target and manipulate four pathways independently, and compared these to manipulation of the entire LHA–LHb projection using *Vglut2-cre* mice. We used two different viral strategies to achieve cell-type specific bilateral optogenetic activation of cell bodies versus terminals, which are as follows: (1) retrograde labeling using AAVretro-DIO-ChR2–mCherry injection into the LHb with somatic stimulation in LHA, and (2) AAV-DIO-ChR2–mCherry injection into to the LHA in combination with axon terminal stimulation (Fig. 3a–c and Extended Data Fig. 5). To specifically probe the distinct role of Esr1^+^ LHA–LHb neurons, we further established a strategy for cell-type specific silencing of Esr1^+^ neurons using expression of the tetanus light chain (TeLC), which we also combined with optogenetic activation of the entire LHA–LHb pathway (Fig. 3a,d and Extended Data Fig. 6a–s).

To first evaluate aversion signaling, we used optogenetic activation in a two-compartment real-time place aversion task, and by confirming previous findings^6^, we found that somatic and terminal optogenetic activation of the entire glutamatergic (that is, Vglut2^+^) LHA–LHb population mice induced significant avoidance of the opto-stimulated compartment. Notably, somatic or axon terminal optogenetic stimulation of Esr1^+^ LHA–LHb neurons induced significant avoidance of the opto-stimulated compartment, whereas activation of Pv^+^, or Gal^+^ or Npy^+^ neuron subtypes failed to induce any avoidance response (Fig. 3e–f and Extended Data Fig. 6m–o,r,z, Video 1 and Supplementary Table 4). To determine whether activation of Esr1^+^ LHA–LHb neurons is necessary for the aversive response, we performed the real-time place aversion task using optogenetic activation of all LHA–LHb neurons in combination with TeLC silencing of Esr1^+^ LHA–LHb neurons. We found that mice with silenced Esr1^+^ LHA–LHb neurons showed no aversion during optogenetic activation of the remaining LHA–LHb pathway (Fig. 3f), demonstrating their necessary role in mediating the aversive signals in the LHA–LHb pathway. Interestingly, although Pv^+^, Gal^+^ or Npy^+^ neuron subtypes failed to induce real-time place aversion, we found that specifically the activation of Npy^+^ LHA–LHb neurons induced significant free-rearing behavior in the open field (Extended Data Fig. 6t–y and Video 2). In addition, we explored the possible role of Pv^+^, Gal^+^ or Npy^+^ in appetitive behaviors and performed optogenetic activation during sucrose consumption. We did not observe any significant effects on sucrose consumption after activation of the Pv^+^, Npy^+^ or Gal^+^ LHA–LHb neurons arguing against a role in reward or food consumption (Extended Data Fig. 6aa,ab).

Because we observed distinct and significant behavioral modulation by optogenetic activation of the Esr1^+^ and Npy^+^ LHA–LHb neurons, we proceeded to investigate their role in inducing persistent behavioral states. We established a state induction protocol based on optogenetic activation of each LHA–LHb pathway for 10 min followed by testing of the immediate and persistent behavioral response (Fig. 3g). We found that continuous activation of the Esr1^+^ LHA–LHb neurons induced specific behaviors associated with aversion (for example, stop and backward movement, and digging behavior) during the stimulation period (Fig. 3h,i). In contrast, we did not observe any aversive behaviors even during continuous activation of the Npy^+^ LHA–LHb neurons (Fig. 3h,i). Notably, continuous, as well as block-wise, activation of Npy^+^ LHA–LHb neurons induced free-rearing behavior during the entire stimulation period (Fig. 3j,k). The increased rearing was not associated with an increased anxiety-like state or induction of long-term negative signals, because optogenetic activation of Npy^+^ LHA–LHb pathway did not affect time spent in the center of the open field nor did it result in avoidance in the conditioned place aversion assay (Extended Data Fig. 6r,s,z). In summary, we found that activation of Npy^+^ LHA–LHb neurons can induce rearing, and that cell-type specific silencing of their activity substantially reduced free rearing under naturalistic conditions (Fig. 3l and Extended Data Fig. 6ac).

To directly address whether the continuous activation of Esr1^+^ or Npy^+^ LHA–LHb neurons could induce a persistent negative signal, we tested the immediate and sustained (24 h later) behavioral response after the optogenetic state induction protocol (10 min). We found significant conditioned place aversion immediately after the activation of the Esr1^+^ LHA–LHb neurons and not after the activation of Npy^+^ LHA–LHb neurons (Fig. 3m). Supporting a central role for Esr1^+^ LHA–LHb neurons in generating a sustained aversive state, we found that the conditioned place aversion persisted for 24 h (Fig. 3n). Overall, these results show the central role of Esr1^+^ LHA–LHb neurons in mediating immediate aversive signals and promoting the persistent aversive state.

## Pathway-specific aversive signals in the PFC

**Fig. 4:**
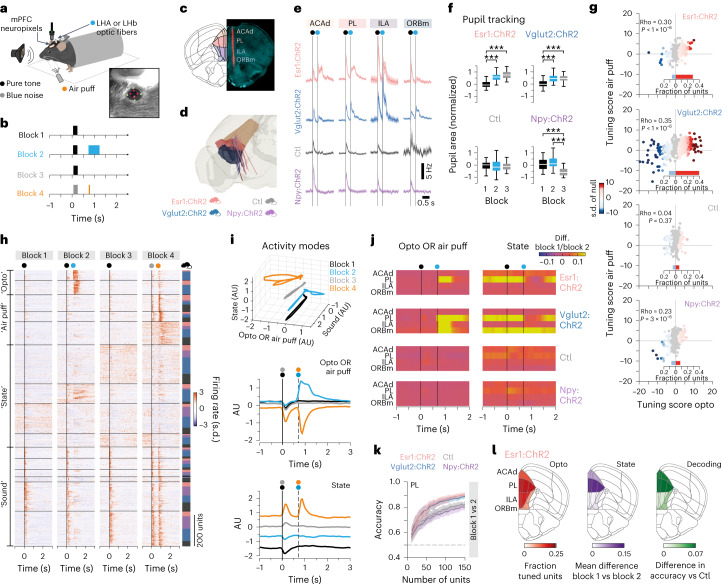
The Esr1^+^ LHA–LHb pathway regulates neural population dynamics in the PFC. **a**, Schematic illustration of the experimental setup. **b**, Behavioral protocol, colors as in **a**. Block 1—presentation of a pure tone (200 ms) at the trial start. Block 2—presentation of a pure tone (200 ms) at the trial start followed by optogenetic stimulation (500 ms) of LHA–LHb neurons 500 ms after the end of the tone. Block 3—identical to block 1. Block 4—presentation of blue noise (200 ms) at the trial start followed by mild air puffs (50 ms) to the eye 500 ms after the end of sound presentation. **c**, Representative example of CM-DiI labeled (red) probe track in DAPI stained (turquoise) brain section. AP = 1.90 mm, Allen reference atlas CCFv3. **d**, Three-dimensional rendering of the tracked anatomical position of 25 Neuropixels probes in the PFC. Brain regions are color coded as in **c**. Bottom, the animal cohorts Ctl, Npy:ChR2 and Vglut2:ChR2 mice, *n*_mice_ = 5 (each); Esr1:ChR2, *n*_mice_ = 10 for all panels. **e**, PSTH (bin size 10 ms) of the firing rate modulation in block 2 for all units (*n*_unit_ = 4,152). Vertical lines, onset of auditory stimulus (black dot), and optogenetic stimulation (blue dot). Mean ± s.e.m. **f**, Difference between the mean pupil area in single trials (blocks 1–3), and the mean pupil area in block 1 (block 1, 50 trials; block 2, 100 trials and block 3, 50 trials). Ctl, Npy:ChR2 and Vglut2:ChR2 mice, *n*_mice_ = 5 (each); Esr1:ChR2 mice, *n*_mice_ = 10. Mean ± s.d. (two-sided Mann–Whitney *U* test with Bonferroni correction (*α* corrected = 0.017), *P* values (block 1 versus block 2, block 1 versus block 3 and block 2 versus block 3, respectively): Esr1:ChR2—*P* = 3.09 × 10^**−**14^, *P* = 1.45 × 10^**−**12^, *P* = 0.01; Vglut2:ChR2—*P* = 1.55 × 10^**−**11^, *P* = 1.15 × 10^**−**8^, *P* = 0.68; Ctl:ChR2—*P* = 0.12, *P* = 0.23, *P* = 0.59; Npy:ChR2—*P* = 0.035, *P* = 1.91 × 10^**−**8^, *P* = 9.61 × 10^**−**15^). **g**, Color-coded tuning score for all units (dots; *n*_unit_ = 1,945) in response to optogenetic stimulation versus air puffs. Gray units, nonsignificant tuning. Bar graphs, fraction of units with significantly negative (light blue) and positive (red) tuning. **h**, Activity-based hierarchical clustering of all units (*n*_unit_ = 1,770; mouse *cre* lines color coded as in **d**; vertical lines—onset of auditory stimulus (black or gray), optogenetic stimulation (blue) and air puffs (orange)). **i**, mPFC neuronal population activity in the four blocks projected in 3D onto three activity modes (‘state’, ‘sound’ and ‘opto OR air puff’; *n*_mice_ = 25, all genotypes; top). mPFC population activity projected onto the ‘opto OR air puff’ mode (middle) and the ‘state’ mode (bottom). Vertical lines—onset of auditory stimulus (black or gray), optogenetic stimulation (blue) and air puffs (orange). **j**, Mapping of the ‘opto OR air puff’ mode and the ‘state’ mode in discrete mPFC subregions. The difference between the projection of block 1 versus block 2 shown as heatmap for each mPFC subregion. Vertical lines, onset of auditory stimulus (black) and optogenetic stimulation (blue). **k**, Decoding (average prediction accuracy) of block identity (block 1 versus block 2) (mean ± s.d., 50 repeated cross-validations; dashed line, 50% chance performance). **l**, Fraction of significantly tuned units across the mPFC subregions in response to optogenetic stimulation in Esr1:ChR2 mice (*n*_mice_ = 10; left). Mean difference of the projection of the ‘state mode’ between block 1 versus block 2 across the mPFC subregions (middle). The difference in decoding (average prediction accuracy) of block identity (block 1 versus block 2) in Esr1:ChR2 versus Ctl mice (right). AP = 1.90 mm, Allen reference atlas CCFv3. *n*_unit_ = number of units, *n*_mice_ = number of mice. Schematic in **a** adapted from Scidraw (https://scidraw.io/). All data were acquired in male mice. For boxplots (**f**), data are shown as median (center line), box (25th and 75th percentiles), whiskers (nonoutlier minimum and maximum) and outliers (>1.5 interquartile range). ***P < 0.001. See also Extended Data Figs. 7–9, Supplementary Videos 3 and 4 and Supplementary Tables 5–9. PSTH, peri-stimulus time histograms. Source data

We next asked whether we could identify shifts in PFC neural dynamics associated with the persistent aversive state induced by the Esr1^+^ LHA–LHb pathway. We focused our study on recording the PFC neural dynamics, based on the key role of the PFC in controlling emotional state through the integration of cortical and subcortical information^22,23^. Our aim was to identify the PFC network effects linked to the aversive state, rather than direct synaptic modulation upon LHA–LHb optogenetic modulation since the PFC is not known to receive monosynaptic inputs from LHb^24^. We used high-density Neuropixels recordings in head-fixed *Esr1-cre* and *Vglut2-cre* mice to map PFC dynamics and behavioral responses to internally generated (that is, pathway-specific optogenetic activation) and externally derived (that is, air puffs) aversive signals (Fig. 4a,b). To validate the aversive component of the PFC neural dynamics, we performed the same experiments in *Npy-cre* and control mice. In block 1 (50 trials), we recorded responses to a pure tone (200 ms, 10 kHz), and in block 2 (100 trials), the response to the same tone followed by optogenetic stimulation (500 ms, 40 Hz pulse train) of the respective LHA–LHb pathway. In block 3 (50 trials), we recorded responses to the tone without optogenetic activation (same as in block 1), while in block 4 (50 trials), we introduced a new auditory stimulus (200 ms blue noise) followed by a mild air puff to the eye (Fig. 4b and Extended data Video 3).

**Extended Data Fig. 7:**
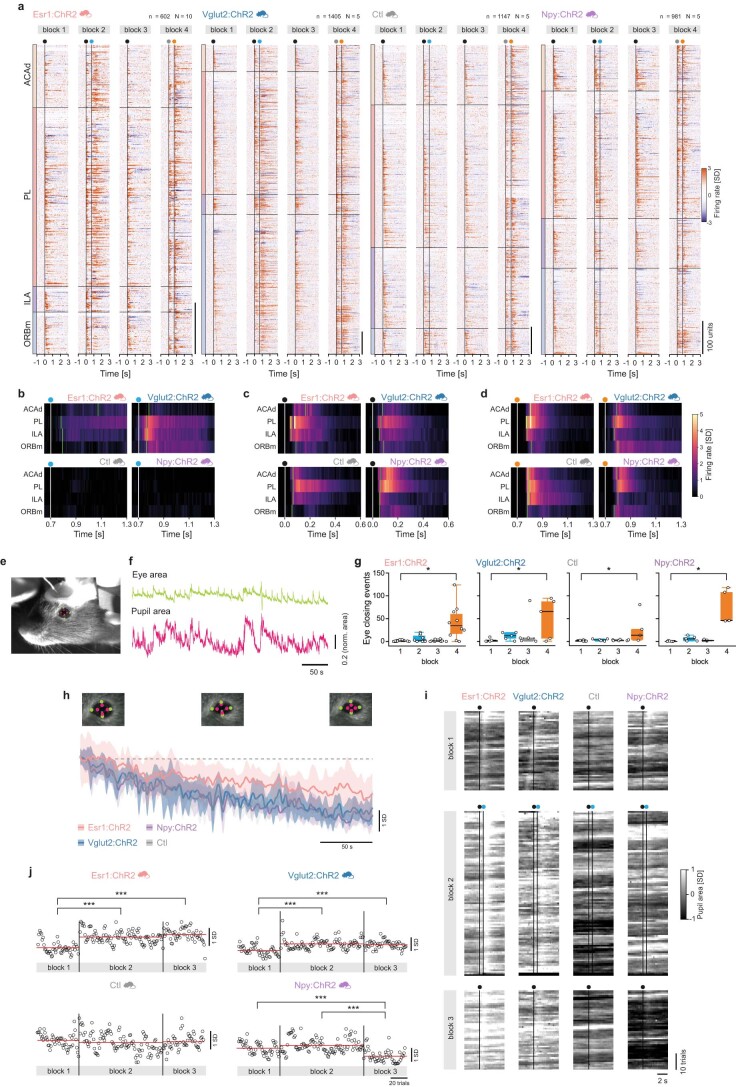
Prefrontal neuronal activity and behavioral response following optogenetic activation of the different LHA-LHb pathways. **(a)** Trial-averaged firing rate (z-scored) for all individual units recorded across the mPFC subregions; Ctl: n = 1147, N = 5; Vglut2:ChR2: n = 1413, N = 5; Npy:ChR2: n = 981, N = 5, Esr1:ChR2: n = 611, N = 10). Colored dots: block specific events; black: sound 1, blue: optogenetic stimulation, gray: sound 2, orange: air puffs. The units are sorted based on their location within a specific mPFC subregion (outlined by horizontal black lines), and their respective peak response latency in block 1. **(b)** Average population firing across the mPFC subregions in (part of) block 2. The detected latency of the onset of the optogenetic responses is indicated with green vertical lines. White vertical line; onset of optogenetic stimulation. Bin size: 10 ms. **(c)** As (b), but detected latency of the onset of the sound response in block 1. White vertical line; sound onset. **(d)** As (b), but detected latency of the onset of the air puff response in block 4. White vertical line; air puff onset. **(e)** Example pupil tracking using DeepLabCut, light green dots: eye markers, pink dots: pupil markers. **(f)** Extracted traces of normalized eye area (top) and normalized pupil area (bottom), colors as in (e). (**g**) Quantification of the number of eye closing events (two-sided Wilcoxon signed ranked test with Bonferoni correction (alpha corrected= 0.0083). Ctl, Npy:ChR2, and Vglut2-:ChR2 mice: N = 5, respectively; Esr1:ChR2: N = 10 mice. p-values: Esr1:Chr2: p = 0.0059; Vglut2:ChR2: p = 0.0078; Ctl: p = 0.002; Npy:ChR2: p = 0.002). (**h**) Eye area over the course of block 4 for each genotype (Ctl, Vglut2:ChR2, Npy:ChR2: N = 5, Esr1:ChR2: N = 10 mice). **(i)** Heatmaps of the mean pupil area (z-scored to block 1) across the individual trials in block 1-3, Ctl, Vglut2:ChR2, Npy:ChR2: N = 5, Esr1:ChR2: N = 10 mice. Colored dots: block specific events; black: sound 1, blue: optogenetic stimulation. **(j)** Mean trial-averaged pupil area (z-scored to block 1) for all individual trials from block 1-3, same trials and mice as in (g). One circle = one trial, block1: n = 50, 2: n = 100, 3: n = 50 trials. Black vertical lines: block limits. Red horizontal lines: mean pupil area in the block. Two-sided Mann-Whitney U test with Bonferroni correction (alpha corrected=0.017), p-values (block1vs2; block1vs3; block2vs3 respectively): Esr1:ChR2: p = 3.09E-14; p = 1.45E-12; p = 0.01; Vglut2:ChR2: p = 1.55E-11; p = 1.15E-8; p = 0.68; Ctl:ChR2: p = 0.12; p = 0.23; p = 0.59; Npy:ChR2: p = 0.035; p = 1.91E-8; p = 9.61E -15). n = number of units, N = number of animals. Abbreviations: medial prefrontal cortex (mPFC), anterior cingulate area, dorsal part (ACAd), prelimbic area (PL), infralimbic area (ILA) and orbitofrontal area, medial part (ORBm), peri-stimulus time histogram (PSTH), standard deviation (SD). For boxplots (**g**), data shown as median (center line), box (25th and 75th percentiles), whiskers (nonoutlier minimum and maximum) and outliers (>1.5 interquartile range). All data acquired in male mice. * p < 0.05, *** p < 0.001. Source data

We focused our analysis on recorded single units in four mPFC subregions—the anterior cingulate area, dorsal part (ACAd), the PL, the infralimbic area (ILA), and the orbitofrontal area, medial part (ORBm; Fig. 4c,d), resulting in 4,152 well-isolated units from 25 mice (Extended Data Fig. 7a). We found strong modulation of mPFC units in response to optogenetic activation only in *Vglut2-cre* and *Esr1-cre* and not in *Npy-cre* mice, while air puffs and auditory stimuli modulated mPFC activity in all four mouse groups (Fig. 4e and Extended Data Fig. 7a). The latency of the mPFC responses to auditory stimulus and air puff was similar across the different mice (Extended Data Fig. 7b–d). Notably, similar to the aversive state induced in freely behaving mice, we found that head-fixed mice with activation of the Esr1^+^ or Vglut2^+^ LHA–LHb pathways developed a persistent behavioral state in block 2 and block 3 measured by a significant increase in pupil size (Fig. 4f, Extended Data Fig. 7e–j, Video 4 and Supplementary Table 5).

**Extended Data Fig. 8:**
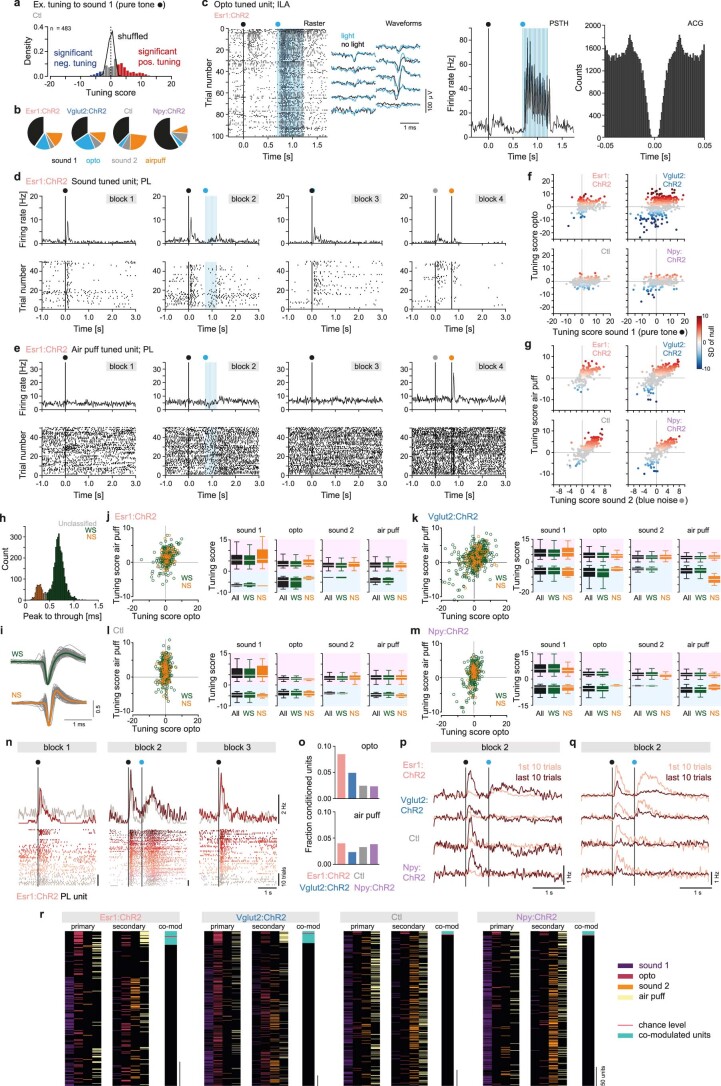
Tuning of mPFC units to sound, LHA-LHb pathway stimulation, and air puffs. (**a**) Tuning scores for all units in Ctl mice (N = 5) fitted with the GLM (n = 482). The tuning to sound 1 (pure tone) is shown. Black curve: tuning scores expected by chance. (**b**) Pie charts of the proportions of primary tuning for all units fitted with the GLM. Ctl: n = 482; Vglut2:ChR2: n = 738; Npy:ChR2: n = 406, Esr1:ChR2: n = 319 units. (**c**) Example ILA unit in an Esr1:ChR2 mouse, significantly tuned to the optogenetic stimulation. From left to right: raster plot aligned to the onset of the sound; mean waveform during (blue) or outside of (black) light application on 10 neighboring recording channels; peri-stimulus-time-histogram (PSTH) aligned to the onset of the sound; auto-correlogram (ACG; bin size: 1 ms). Colored dots: block specific events; black: sound 1, blue: optogenetic stimulation, blue vertical lines: light pulses (5 ms). (**d**) Example PL unit in an Esr1:ChR2 mouse, significantly tuned to sound 1 (pure tone; block 1-3). Top: PSTHs, bottom: raster plots of 50 trials. Colored dots: block specific events; black: sound 1, blue: optogenetic stimulation, gray: sound 2, orange: air puffs. (**e**) As **(d**) but for an example PL unit in an Esr1:ChR2 mouse significantly tuned to air puffs (block 4). (**f**) Scatterplots of the tuning of all units fitted with the GLM (n = 1945**)** in response to optogenetic stimulation (y axis) vs sound 1 (pure tone; y axis). One dot = one unit. Units significantly tuned to optogenetic stimulation are color coded (standard deviation from the null distribution). Gray units: non-significant tuning. (**g**) As **(f**) but for sound 2 (blue noise) vs air puffs. Units significantly tuned to air puffs are color coded (standard deviation from the null distribution). Gray dots: units without significant tuning. **(h**) Distribution of the peak to through duration for all recorded mPFC units (n = 4152, N = 25). Units with narrow waveform (peak to through < 0.38 ms) were classified as narrow spiking units (NS, orange) and units with wide waveform (peak to through duration > 0.44 ms) as wide spiking (WS, green) units. (**i**) Mean waveform of the WS (green trace) and NS (orange trace) units, respectively. Gray traces: waveforms of 100 randomly picked units/cell type. All waveforms are peak normalized. (**j-m**) Left: scatterplots of the tuning of all WS (green) and NS (orange) units fitted with the GLM (Table 7 and 8) in response to optogenetic stimulation (x axis) vs air puffs (y axis) for Esr1:ChR2 (**j**), Vglu2:ChR2 (**k**), Ctl (**l**), and Npy:ChR2 mice (**m**), respectively. One circle = one unit. Right: The mean tuning scores of the significantly tuned units, showing the mean negative and mean positive tuning scores, respectively. Right, detail of the tuning scores (postive and negative tuning scores, All units: black, WS: green, NS: orange) for sound1, optogenetic stimulation, sound2 and air puff for each genotype. (**n**) Response profile of example PL unit in an Esr1:ChR2 mouse, across block 1-3. Top: PSTH, bottom: raster, both with color-coding of the trial progression within the blocks (lighter to darker, 10 trials/color). Colored dots: block specific events; black: sound 1, blue: optogenetic stimulation; vertical black lines: event onset. Bin size: 10 ms. (**o**) The fraction of conditioned units. Conditioned units displayed significantly (Wilcoxon signed rank-test) increased or decreased response to the sound and optogenetic manipulation in block 2 (top) or to the sound and air puffs in block 4 (bottom) over the trials within the respective block. (**p**) Mean response profile of the conditioned units that over the trials displayed significantly increased response to the sound in block 2. Mean of the first (light color) and last (dark color) 10 trials in block 2 are shown. Colored dots: block specific events; black: sound 1, blue: optogenetic stimulation, vertical black lines: event onset. Bin size: 10 ms. (**q**) As (**p**) but for the units that over the trials displayed significantly decreased response to the sound in block 2. (**r**) The primary and secondary tunings (to sound 1, optogenetic stimulation, sound 2, or air puff respectively), and the optogenetic/air puff co-modulation for all units fitted with the GLM (n = 1945). The units are grouped based on genotypes. Red bar: number of co-modulated units expected by chance. n = number of units, Ctl, VGlut2:ChR2, Npy:ChR2: N = 5, Esr1:ChR2: N = 10 mice in all panels with group data. All data acquired in male mice. For boxplots (**j, k, l, m**), data shown as median (center line), box (25th and 75th percentiles), whiskers (nonoutlier minimum and maximum) and outliers (>1.5 interquartile range). Source data

To quantify the tuning of single mPFC units, we used a generalized linear model (GLM) to fit the unit firing rate (binned) with the events in the four blocks (two auditory stimuli, optogenetic activation and air puff) as regressors^25^. Tuning scores were computed for all recorded units fitted with the GLM (*n*_unit_ = 1,945) and substantially tuned units were identified (Extended Data Fig. 8a–e). This revealed a significant mean tuning score to the optogenetic activation of the Esr1^+^ and Vglut2^+^ LHA–LHb pathways (Fig. 4g, Extended Data Fig. 8f,g and Supplementary Table 6), and spike waveform classification showed significant tuning of both narrow-spiking putative inhibitory interneurons (*n*_unit_ = 564) and wide-spiking putative pyramidal neuron units (*n*_unit_ = 3,418; Extended Data Fig. 8h–m and Supplementary Tables 7 and 8). We found that opto-modulated mPFC units in block 2 trials showed a number of conditioned units^26^ in *Vglut2-cre* and *Esr1-cre* mice (Extended Data Fig. 8n–q). To identify neuronal responses shared between the external (that is, air puff) and internal (that is, optogenetic activation) aversive stimuli, we computed primary and secondary tunings for each GLM-fitted unit and found that *Vglut2-cre* and *Esr1-cre* mice showed substantially comodulated aversion units (Extended Data Fig. 8r).

**Extended Data Fig. 9:**
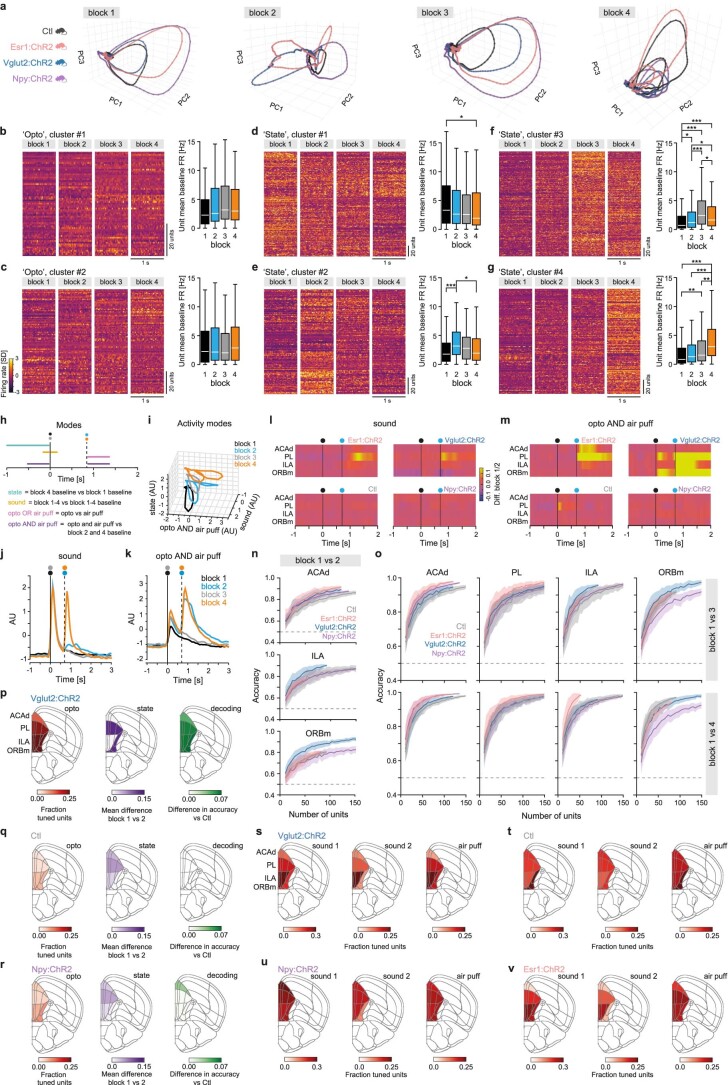
Prefrontal population dynamics in response to air puffs or optogenetic stimulation of distinct LHA-LHb pathways. (**a**) Trajectories of the neuronal population activity (trial averaged) in block 1-4; the first three principal components (PC) are shown. (**b-g**) Left: Trial-averaged baseline (the 1 s preceding sound onset, n = 50 trials) firing rate (z-scored) in block 1-4 for all individual units in specific clusters (bin size 10 ms, see also Fig. 4h). The units are sorted as in Fig. 4h. Right: mean baseline firing rate in block 1-4, respectively. SD (error bar) and significance level (tow-sided Wilcoxon signed-ranked test with Bonferroni correction). (**b**), ‘Opto’ cluster #1 (n = 65 units), (**c**) ‘Opto’ cluster #2 (n = 93 units), (**d**) ‘State’ cluster #1 (n = 195 units), (**e**) ‘State’ cluster #2 (n = 128 units), (b) ‘State’ cluster #3 (n = 253 units), (**g**) ‘State’ cluster #4 (n = 150 units). **h**. Schematic outline of the task events used to calculate the activity modes. **i**. mPFC neuronal population activity in the four blocks projected in 3D onto the three activity modes (state, sound, and aversive AND signal; N = 25 mice). **j**. The mPFC neuronal population activity in the four blocks (1-4, color coded, bin size: 10 ms) projected in 1D onto the Sound mode. Colored dots: block specific events; black: sound 1, blue: optogenetic stimulation, gray: sound 2, orange: air puffs. Vertical lines: event onset. See also Fig. 4h. (**k**) as **(j**) but with projection on the ‘opto AND air puff’ mode. (**l**) Detailed investigation of the sound mode; involvement of mPFC subregions and LHA-LHb pathways. Difference of neuronal population activity between block 1 and 2, projected onto the sound mode; bin size: 50 ms. Colored dots: block specific events; black: sound 1, blue: optogenetic stimulation; Vertical lines: event onset. (**m**) as (**l**) but with projection on the ‘aversive AND signal’ mode. (**n**) Decoding (average prediction accuracy) of block identity (block 1 vs 2) using baseline activity in the ACAd, ILA, and ORBm. Accuracy is plotted as a function of the number of units included in the logistic regression. Mean ± SD: 50 repeated cross-validations. Dashed line: 50% chance performance. (**o**) Top: decoding (average prediction accuracy) of block identity (block 1 vs 3) using baseline activity in the ACAd, PL, ILA, and ORBm. Accuracy is plotted as a function of the number of units included in the logistic regression. Mean ± SD: 50 repeated cross-validations. Dashed line: 50% chance performance. Bottom: as top, but block 1 vs 4. (**p**) Fraction of significantly tuned units across the mPFC subregions in response to optogenetic stimulation in Vlut2:ChR2 mice (left). Mean difference of the projection of the ‘state mode’ between block 1 vs block 2 across the mPFC subregions (middle). Difference in decoding (average prediction accuracy) of block identity (block 1 vs 2) in Vlut2:ChR2 vs Ctl mice (right). AP = 1.90 mm, Allen reference atlas CCFv3. (**q**-**r**) As (**p**), but for Ctl (**q**), and Npy:ChR2 mice (**r**). (**s**) Fraction of units across the mPFC subregions in Vglut2-cre mice with significant tuning to sound 1(left), sound 2 (middle), and air puffs (right). (**t-v**) As **(s**), but for Ctl (**t**), Npy:ChR2 (**u**), and Esr1:ChR2 (**v**) mice. n = number of units, N = number of animals. All data acquired in male mice. For boxplots (**b-g**), data shown as median (center line), box (25th and 75th percentiles), whiskers (nonoutlier minimum and maximum) and outliers (>1.5 interquartile range).* *p* < 0.05, **p < 0.01, ***p < 0.001. Source data

We next analyzed how aversive signals (internal optogenetic versus external air puff) shifted the mPFC population dynamics. We used principal component analysis (PCA) to visualize how neural activity evolved over the four blocks. We found similar activity trajectories during blocks with auditory stimuli (blocks 1 and 3) and in the block with air puffs across all four mouse groups (block 4; Extended Data Fig. 9a). In contrast, optogenetic drive of the Esr1^+^ and Vglut2^+^ LHA–LHb pathways resulted in dramatically divergent trajectories compared to *Npy-cre* and control mice (that is, in block 2), indicating prominent modulation of neural activities across many units and mPFC subregions. To better understand the contribution of single units to the divergent population dynamics, we performed hierarchical clustering of the activity profile of all GLM-fitted mPFC units (*n*_unit_ = 1,770). This unbiased classification identified two distinct clusters containing opto-modulated units (‘opto’ clusters), showing time-locked or sustained opto-modulation, and these units belonged to *Vglut2-cre* and *Esr1-cre* mice (block 2; Fig. 4h). Interestingly, we observed five clusters (‘state’ clusters) with units showing a significant increase in baseline firing rate in one block or ramping baseline activity across blocks (Extended Data Fig. 9b–g and Supplementary Table 9).

Modulation of the spontaneous baseline firing could be a reflection of block-specific population activities associated with distinct internal states^27^. We, therefore, next adapted a neural activity mode decomposition analysis to compare the main activity modes (‘sound’, ‘opto OR air puff’, ‘opto AND air puff’ and ‘state’) revealed by the clustering, across genotypes and mPFC subregions. For this analysis, the high-dimensional neuronal activity space (dimensions = the number of recorded neurons) was reduced to a low-dimensional space (3D or 1D) where the axes were designed to maximally separate the two task epochs used to define the activity modes. The population activity of specific blocks, genotypes and/or mPFC subregions was projected onto the activity modes, which showed an accurate representation of block-specific events (sound, optogenetic and air puff-related activity) by their respective modes (Fig. 4i and Extended Data Fig. 9h–m).

We found specific differences in the projected neural activity between block 1 and block 2 onto the activity modes for the ‘opto OR air puff’, for the ‘opto AND air puff’ and for the ‘state’. For example, we found that the modulation along the activity mode capturing the optogenetic effect (‘opto OR air puff’) was specific to the activation of Esr1^+^ or Vglut2^+^ LHA–LHb neurons in block 2. Interestingly, the ‘state’ mode (baseline activity in block 4 versus block 1) captured distinct population activity modes in each of the four blocks, with complete separation of the block projections across time (Fig. 4i). When we analyzed the region distribution of these activity modes (‘opto OR air puff’; ‘state’), we found that they were detected primarily in the PL in *Esr1-cre* mice, whereas they were widely distributed in the mPFC in *Vglut2-cre* mice (Fig. 4j and Extended Data Fig. 9j,k). This supports the notion that the ‘state’ activity mode reflects block-specific population activities associated with distinct behavioral and internal aversive states in mPFC.

To further address the region-specific encoding of the behavioral aversive state in mPFC, we used logistic regression to decode block identity from the baseline activity (activity during 2 s preceding auditory stimulus). We found that baseline activity in PL allowed for increased accuracy in decoding block 1 versus block 2 identity in *Esr1-cre* and *Vglut2-cre* mice (Fig. 4k). No differences in accuracy between the genotypes were found when repeating the decoding of block identity (block 1 versus 2, 1 versus 3 and 1 versus 4) using the baseline activity of the other mPFC subregions (Extended Data Fig. 9n,o). It is likely that the observed increase in decoding accuracy (that is, block 1 versus block 2) based on the baseline activity of PL units reflects the induction of a distinct neural activity state that reflects some aspects of the aversive behavioral state induced by activation of the Esr1^+^ LHA–LHb pathway. The opto-modulation, negative state signature and region decoding together reinforce that activation of Esr1^+^ LHA–LHb neurons has profound and specific effects on the unit and population activities in PL (Fig. 4l and Extended Data Fig. 9p–v), revealing a region-specific signature of the aversive state in mPFC.

## Sexually dimorphic stress state is encoded in Esr1^+^ LHA–LHb subtype neurons

**Fig. 5:**
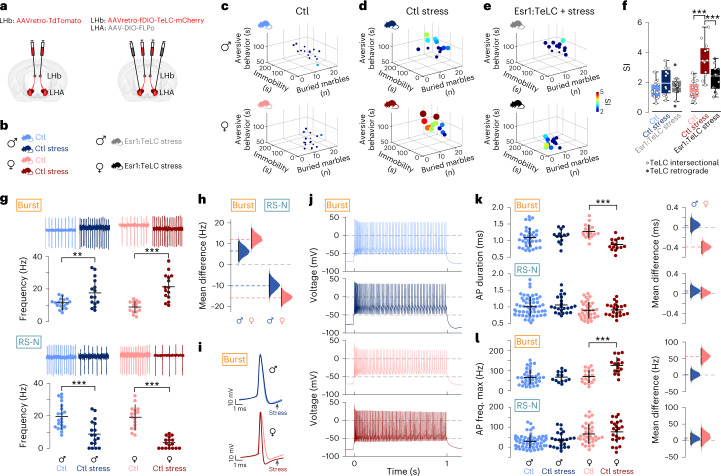
Sexually dimorphic stress sensitivity in Esr1^+^ LHA–LHb neurons. **a**, Experimental design for retrograde viral labeling of LHA–LHb neurons (left), and TeLC silencing of Esr1^+^ LHA–LHb neurons using intersectional strategy (right). See Extended Data Fig. 6c for alternative retrograde TeLC strategy. **b**, Animal cohorts used in behavioral phenotyping. Ctl mice (both sexes) were subjected to viral injection (**a**, left). Ctl stress mice (both sexes) were subjected to the same viral injection (**a**, left) and thereafter subjected to the stress paradigm. Mice (both sexes) with TeLC silencing of Esr1^+^ LHA–LHb neurons (Esr1:TeLC stress) were subjected to the stress paradigm. The plots in **e** and **f** show combined data of TeLC silencing experiments with the intersectional (**a**, right) and with the retrograde strategy (Extended Data Fig. 6c). **c**–**e**, Behavioral phenotyping 24 h after ending of the stress paradigm. Three-dimensional scatter plots summarizing total time aversive behavior in the looming stimuli test (*y* axis), immobility in the FST (*x* axis) and marble burying (*z* axis). Data points, colored and sized by the SI. Ctl (**c**), *n*_mice_ = 15 male, 16 female mice; Ctl stress (**d**), *n*_mice_ = 12 male, 12 female mice and Esr1:TeLC stress (**e**), *n*_mice_ = 12 male, 13 female mice. **f**, Quantification of the SI. The stress paradigm significantly increased the SI in female mice (Ctl versus Ctl stress female mice, *P* = 2.39 × 10^−8^, one-way ANOVA with Tukey’s Multiple comparisons test). Female mice with TeLC silencing of Esr1^+^ LHA–LHb neurons displayed significantly reduced SI (Ctl stress females versus Esr1:TeLC stress females, *P* = 0.0016, one-way ANOVA with Tukey’s multiple comparisons test). Same mice as in **c**–**e**. **g**–**l**, Electrophysiological characterization of intrinsic properties of Esr1^+^ LHA–LHb neurons in stressed and control female and male mice (retrogradely labeled LHA–LHb neurons as in Extended Data Fig. 10l). **g**, Representative cell-attached traces of significantly increased tonic firing of Esr1^+^ Burst type neurons and decreased tonic firing of Esr1^+^ RS-N type neurons in stressed female and male mice (top). Quantification of the mean firing rate, Burst type neurons—Ctl stress female (*n*_neuron_ = 15) versus Ctl female (*n*_neuron_ = 14), *P* = 0.0; Ctl stress male (*n*_neuron_ = 15) versus Ctl male (*n*_neuron_ = 18), *P* = 0.0044; RS-N type neurons—Ctl stress female (*n*_neuron_ = 17) versus Ctl female (*n*_neuron_ = 16), *P* = 0.0; Ctl stress male (*n*_neuron_ = 17) versus Ctl male (*n*_neuron_ = 20), *P* = 0.0002; two-sided DABEST test, mean ± s.d.; bottom. **h**, Quantification of the stress-induced change in the tonic firing frequency of Esr1^+^ Burst type (left) and Esr1^+^ RS-N type (right) neurons in female and male mice. The difference in firing between Ctl and Ctl stress females (pink) and Ctl and Ctl stress males (blue) is plotted. **i**, Representative rheobase APs of Burst type Esr1^+^ neurons. Quantified in **k**, colors as in **g**. **j**, Representative firing patterns of Burst type Esr1^+^ neurons at maximal depolarization. Quantified in **l**, colors as in **g**. **k**, The AP duration of Esr1^+^ Burst type neurons is significantly decreased in stressed females. Quantification of the AP duration, Burst type neurons—Ctl stress female (*n*_neuron_ = 15) versus Ctl female (*n*_neuron_ = 18), *P* = 0.0; two-sided DABEST test, mean ± s.d. RS-N type neurons—Ctl stress male, *n*_neuron_ = 22; Ctl male, *n*_neuron_ = 56; Ctl stress female, *n*_neuron_ = 22 and Ctl female, *n*_neuron_ = 36. **l**, The maximal firing frequency of Esr1^+^ Burst type neurons is significantly increased in stressed females. Quantification of the maximal firing frequency—Ctl stress versus Ctl female mice, *P* = 0.0, two-sided DABEST test, mean ± s.d., same neurons as **i** (**k**). *n* = number of neurons, *N* = number of mice. Data acquired in male versus female mice as stated in respective panel. For boxplots (**f**), data shown as median (center line), box (25th and 75th percentiles), whiskers (nonoutlier minimum and maximum) and outliers (>1.5 interquartile range). Modified Gardner–Altman plots (**h**,**k**,**l**), distribution of bootstrap-sampled mean differences (area—pink, Ctl stress female versus Ctl female; blue, Ctl stress male versus Ctl male), mean of bootstrap sampling distribution of (circle) and the 95% CI (vertical black line). **P < 0.01, ***P < 0.001. See also Extended Data Fig. 10, Supplementary Video 5 and Supplementary Table 10. Source data

**Extended Data Fig. 10:**
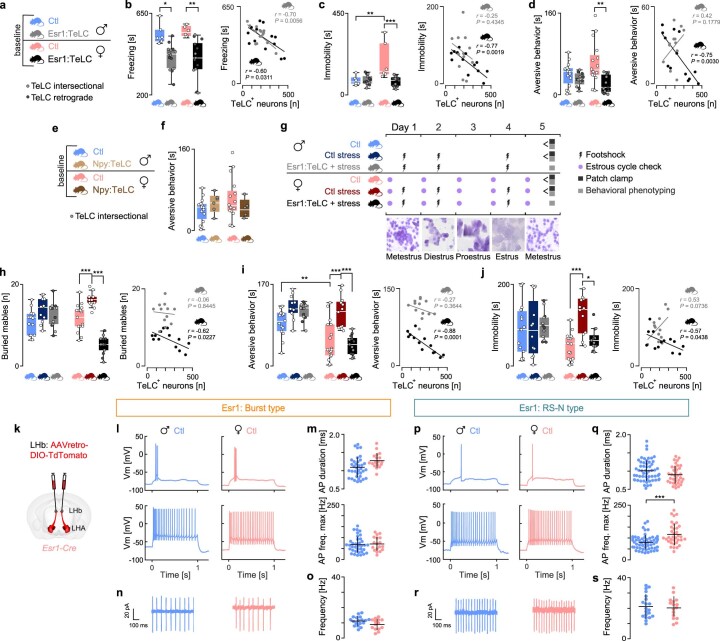
Aversive behaviors, behavioral stress response, and *ex vivo* electrophysiological characterization of baseline properties of Esr1+ LHA-LHb neurons in male and female mice. (**a**) Animal cohorts used in behavioral phenotyping (top), and the viral strategy for TeLC silencing of Esr1+ LHA-LHb neurons (bottom). (**b**) Fear conditioning. Left: TeLC silencing of Esr1+ LHA-LHb neurons significantly reduced the total time freezing during fear conditioning extinction both in male and female mice (male Esr1:TeLC vs Ctl male, p = 0.0163; Esr1:TeLC female vs female Ctl, p = 0.0028, one-way ANOVA with Tukey’s Multiple comparisons test. Ctl: N = 6 male mice, 6 female mice, Esr1:TeLC: N = 14 male mice, 13 female mice). Right: the relationship (Pearson correlation coefficient) between the total time spent freezing during fear conditioning extinction and the number of TeLC+ neurons for male (gray) and female (black) mice, respectively. (**c**) Acoustic startle test. Left: TeLC silencing of Esr1+ LHA-LHb neurons significantly reduced the total time spent immobile induced by the presentation of a novel tone in female mice (one-way ANOVA with Tukey’s Multiple comparisons test, Ctl male vs Ctl female, p = 0.0029; Esr1:TeLC female vs Ctl female, p = 2.3E-4). Right: relationship (Pearson correlation coefficient) between the immobility during the acoustic startle test and the number of TeLC+ neurons for male (gray) and female (black) mice, respectively. Same mice as in (**b**). (**d**) Looming stimuli test. Left: TeLC silencing of Esr1+ LHA-LHb neurons in female mice significantly reduced the time spent in aversive behaviors (Ctl female vs Esr1:TeLC female, p = 0.0013, one-way ANOVA with Tukey’s Multiple comparisons test. Ctl: N = 15 male mice, 16 female mice, Esr1:TeLC: N = 14 male mice, 13 female mice). Right: relationship (right, Pearson correlation coefficient) between the time spent in aversive behaviors and the number of TeLC+ neurons for male (gray) and female (black) mice, respectively. (**e**) Animal cohorts used for baseline phenotyping in the looming stimuli test (top), and the viral strategy for TeLC silencing of Npy+ LHA-LHb neurons (bottom). (**f**) TeLC silencing of Npy+ LHA-LHb neurons did not influence the time spent in aversive behaviors in the looming stimuli test. Npy:TeLC: N = 6 male mice, 5 female mice, Ctl: N = 15 males mice, 16 female mice (same Ctl mice as in (**e**)). (**g**) Experimental design and animal cohorts used for behavioral phenotyping and *ex vivo* electrophysiological recordings. **(h-j)** Behavioral phenotyping 24 h after ending of the stress paradigm (see also Fig. 5c). Ctl: N = 15 male, 16 female, Ctl stress: N = 12 male mice, 12 female mice; Esr1:TeLC stress: N = 12 male mice, 13 female mice; all mice same as in Fig. 5c-f. One-way ANOVA with Tukey’s Multiple comparisons for all statistics. Right: the relationship (Pearson correlation coefficient) between the number of TeLC+ neurons and behavioral variables for male (gray) and female (black) mice, respectively. (**h**) Marble burying test: Ctl female vs Ctl stress female: p = 8.94E-4; Ctl stress female vs Esr1:TeLC stress female: p = 1.83E-13. (**i**) Looming stimuli test: Ctl male vs Ctl female: p = 0.0062; Ctl stress female vs Ctl female: p = 6.41E-6; Ctl stress female vs Esr1:TeLC stress female: p = 1.22E-6. (**j**) Forced swim test: Ctl stress female vs Ctl female: p = 7.86E-4; Ctl stress female vs Esr1:TeLC stress female: p = 0.0254. (**k**) Experimental design for retrograde labeling Esr1+ LHA-LHb neurons used for *ex vivo* electrophysiological recordings. (**l**) Representative baseline whole-cell firing traces at rheobase (top), whole-cell firing patterns at maximal depolarization (bottom) of Burst type Esr1+ LHA-LHb neurons in male (blue; n = 37 neurons) and female (pink; n = 18 neurons). (**m**) No significance difference in AP duration and maximal firing frequency of Burst type neurons were found between male (n = 37) and female (n = 18) mice (DABEST test). (**n**) Representative cell-attached baseline tonic firing traces of Burst type Esr1+ LHA-LHb neurons in male (blue) and female (pink) mice. (**o**) No significance difference in tonic firing frequency of Burst type neurons were found between male (n = 18) and female (n = 14) mice (DABEST test). (**p**) Representative baseline whole-cell firing traces at rheobase (top), whole-cell firing patterns at maximal depolarization (bottom) of RS-N type Esr1+ LHA-LHb neurons in male (blue) and female (pink) mice. (**q**) No significance difference in AP duration of RS-N type neurons were found between male and female mice, while neurons in female mice displayed a significant higher maximal firing frequency than male mice (female vs male mice, p = 0.0004, DABEST test). Female mice: n = 57, male mice: n = 34. (**r**) Representative cell-attached baseline tonic firing traces of RS-N type Esr1+ LHA-LHb neurons in male (blue) and female (pink) mice. (**s**) No significance difference in tonic firing frequency of RS-N type neurons were found between male (n = 20) and female (n = 16) mice (DABEST test). n = number of neurons, N = number of animals. The data acquired in male and female mice as indicated in panel. For boxplots (**b-f**, **h-j**), data shown as median (center line), box (25th and 75th percentiles), whiskers (nonoutlier minimum and maximum) and outliers (>1.5 interquartile range). *p < 0.05, **p < 0.01, ***p < 0.001. Source data

The sexual dimorphism in Esr1^+^ circuits and sex-selective gene expression^17,18^ in combination with our finding on the aversive state induction prompted us to investigate the role of Esr1^+^ LHA–LHb neurons in mediating the sensitivity to aversive stimuli and to develop a sustained stress state. We found that Esr1^+^ LHA–LHb neurons were necessary for fear conditioning in male and female mice; however, in the case of milder aversive stimuli (new auditory stimulus and looming stimulus), we found that silencing Esr1^+^ LHA–LHb neurons primarily reduced aversive responses in female mice (Extended Data Fig. 10a–f). To follow up the sensitivity to mild aversive stimuli in female mice, we established a stress paradigm consisting of repeated exposure to unpredictable mild shocks to induce a persistent shift in the stress response, followed by a battery of behavioral tests performed to quantify sex-specific behavioral differences (Fig. 5a,b and Extended Data Fig. 10g). We synchronized the start of the shock protocol and the behavioral phenotyping in female mice based on cytological criteria (metestrus phase) to minimize possible effects of the cycling of sex hormones. We found that female mice developed a persistent stress state in response to the unpredictable stressors, including substantially increased marble burying, aversive behaviors in the looming test and immobility in the forced swim test 24 h after the last unpredictable shock exposure (Fig. 5c,d, Extended Data Fig. 10h–j and Video 5). We found that cell-type-specific silencing (TeLC) of Esr1^+^ LHA–LHb neurons reduced the negative impact of the unpredictable stressors on behavior in female mice (Fig. 5e). To generate a metric of the stress state, we combined data from the three behavioral tests to establish a stress index (SI). This quantification showed that only female mice developed a significant and persistent stress state and that this state required the activity of Esr1^+^ LHA–LHb neurons as shown by the cell-type-specific silencing (Fig. 5f).

We then asked whether the Esr1^+^ Burst and the RS-N neuron types showed cell-type as well as sex-specific shifts in their intrinsic properties in response to the stress state. We followed the same stress induction protocol, exposing a separate cohort of male and synchronized female mice to the unpredictable mild shock protocol. To capture the sustained differences in intrinsic properties, we performed ex vivo cell-attached and whole-cell recordings from genetically labeled Esr1^+^ LHA–LHb neurons 24 h after the last unpredictable shock as in the behavioral phenotyping (Extended Data Fig. 10g). We first confirmed that the electrophysiological parameters allowed us to classify Esr1^+^ LHA–LHb neuron types also in female mice under baseline conditions (Extended Data Fig. 10k–s and Supplementary Table 10). We then recorded in the cell-attached mode to define the putative adaptation of the spontaneous firing frequencies in the stress state. Unexpectedly, we found that Burst and RS-N types were inversely affected by the stress state as Burst-type neurons increased and RS-N-type neurons decreased their firing in both sexes (Fig. 5g,h). The increased firing of the Burst neuron type supported their role as the primary candidate in mediating aversive stimuli. We also noticed that the increased firing of Burst-type neurons was more prominent in female compared to male mice. We therefore next quantified the intrinsic properties from whole-cell recordings to determine whether the observed excitability adaptation could reflect the observed behavioral stress sensitivity in female mice. We found a significant decrease in the AP duration and a significant increase in the maximum firing frequency specifically in Burst-type Esr1^+^ neurons from stressed female mice (Fig. 5i–l). Interestingly, we only found significant differences in the Burst-type Esr1^+^ neurons in female mice, and no statistically significant changes in Burst-type or RS-N-type Esr1^+^ neurons in male mice. These results suggest that Esr1^+^ Burst type neurons, through shorter AP duration and increased maximum firing, became persistently more excitable upon induction of the sustained stress state in female mice. In summary, our findings show that the activity of Esr1^+^ LHA–LHb neurons is necessary for the sex-specific sensitivity to develop the stress state, and this state is associated with a specific shift in the intrinsic properties of Esr1^+^ LHA–LHb Burst type neurons in female mice.

## Discussion

We have uncovered an extensive diversity in the LHA–LHb pathway, establishing six neuron types with discrete physiology, molecular profile and projection pattern. Using candidate molecular markers from Patch-seq data, we were able to visualize the cell-type-specific organization and determine the role of LHA–LHb neuron subtypes in the control of immediate as well as sustained emotional behaviors. Our classification revealed a specialized organization of the glutamatergic LHA–LHb pathway and showed the discrete functional role for Esr1^+^ versus Npy^+^ LHA–LHb neuron subtypes. We found that the aversive signals induced by activation of the entire glutamatergic (Vglut2^+^) LHA–LHb pathway are mediated by the Esr1^+^ subpopulation, which are both necessary and sufficient to signal immediate negative signals as well as induce persistent states of aversion or stress. In contrast, we found that the Npy^+^ LHA–LHb pathway does not impact avoidance or negative signals and instead controls rearing, as optogenetic activation of the Npy^+^ LHA–LHb pathway induced free rearing, and the cell-type-specific silencing resulted in decreased rearing in the open field. Rearing is observed in naturalistic behaviors linked to exploration and has been proposed to signal novelty and risk assessment, for example, associated with escape or defensive responses, and it is interesting to note that stimulation of escape or fear-related centers (for example, periaqueductal gray (PAG)) can increase rearing behavior^28,29^. We conclude that Npy^+^ LHA–LHb neurons mediate signals linked to exploration, although the relationship to uncertainty or escape-related states remains to be established.

The distinct behavioral effects we observed (that is, aversion in Esr1^+^ versus rearing in Npy^+^) could result from the topographically organized targeting of different LHb domains leading to the recruitment of divergent subcortical or cortical circuits. In combination with our findings on the role of LHA–LHb neuron subtypes in persistent aversive behavioral states, this prompted us to probe how internally generated negative signals although the LHA–LHb pathway indirectly affects mPFC neural dynamics. The PFC is proposed to be a major hub for representing and integrating emotional signals, and we, therefore, chose to perform Neuropixels recordings in PFC in combination with optogenetic activation of the genetically distinct LHA–LHb pathways. We were able to generate a persistent negative state by optogenetic stimulation of the entire Vglut2^+^ LHA–LHb pathway in head-fixed mice, which was reflected in a strong modulation of the neural signal across several PFC subregions. In comparison, optogenetic activation of the Esr1^+^ LHA–LHb pathway induced a behavioral response of immediate as well as persistent aversion that primarily modulated the neural dynamics in the PL region. Our results support a model where negative emotional states are represented by specific ensembles in mPFC^30,31^, which are under the influence of specific LHA–LHb subpopulations.

These observations prompted us to further investigate a possible link between the Esr1^+^ LHA–LHb pathway and the behavioral manifestation of persistent negative states and stress. It is known that exposure to unpredictable negative events can precipitate a chronic stress response and shift the circuit dynamics into a maladaptive state^32^. To test the role of Esr1^+^ LHA–LHb neuron subtypes in stress-related behavior, we established a behavioral paradigm to specifically capture sex-specific differences in the sensitivity to develop a persistent stress-related state at the behavioral as well as the cellular level. We found that a paradigm based on repeated exposure to unpredictable mild foot shocks revealed a substantially increased sensitivity in female mice to develop behavioral correlates of stress. Notably, the behavioral stress state was dependent on the activity of Esr1^+^ LHA–LHb neurons. While we have identified that Esr1-expression marks two electrophysiologically distinct LHA–LHb neuron types (that is, the Burst and the RS-N type), we found that the stress state was associated with altered intrinsic properties specifically in the Burst-type Esr1^+^ neurons.

We found that Burst-type Esr1^+^ LHA–LHb neurons show persistent stress-induced shifts, suggesting that these have a role in mediating stress-related signals to downstream circuits. In a broader context, it is possible that Esr1^+^ neurons in several subcortical circuits can share a similar function in the regulation of behavioral states, specifically through serving as nodes of sexually dimorphic gene expression and neuron function^17,18^. Further studies using intersectional and viral strategies based on the candidate markers we have presented, which allow for differential targeting of Burst versus RS-N type Esr1^+^ LHA–LHb neurons, will be required to establish causal links between the cell-type-specific state and the behavioral manifestation of stress.

It is interesting to note that negative emotional states, such as the chronic expression of stress or mood disorders, can emerge from similar patterns of persistent or Burst-like activity in several nodes in the LHA–LHb PFC circuitry. For example, persistent activity in hypothalamic neurons is found in fear and threat situations^33^ and persistent activity in the PL region could underlie some of the sex differences in fear^34^. Similarly, increased bursting activity in the LHb is a marker of depression-like behavior in animal models^9,35^. How negative signals in LHA–LHb produce acute versus persistent network effects in the mPFC that control the emotional state remains to be determined, and the role of region-specific signals needs to be further investigated, particularly because the functional identity of mPFC subregions is still debated^36^.

Taken together, we propose that Ers1^+^ LHA–LHb neurons are central in signaling negative value, are necessary and sufficient to induce a persistent negative state and mediate the sex-dependent sensitivity to develop behavioral and cellular maladaptive responses to unpredictable stressors. Our multimodal classification of the LHA–LHb pathway establishes a road map for understanding how diverse neuron types contribute to the complex dimensions of the emotional state and to sex differences in circuit function linked to stress.

## Methods

### Animals

All procedures and experiments on animals were performed according to the guidelines of the Stockholm Municipal Committee for animal experiments and the Karolinska Institutet in Sweden (approval numbers N166/15, 155440-2020 and 7362-2019). Adult mice aged 2–5 months were used: *Esr1-cre* (B6N.129S6(Cg)-*Esr1*^*tm1.1(cre)And*^/J; Jackson Laboratory Stock, 017911), *Npy-cre* (B6.Cg-*Npy*^*tm1(cre)Zman*^/J; Jackson Laboratory Stock, 027851), *Pv-cre* (B6;129P2-*Pvalb*^*tm1(cre)Arbr*^/J; Jackson Laboratory Stock, 008069), *Gal-cre* (*Tg(Gal-cre)KI87Gsat*), *Vglut2-cre* (STOCK *Slc17a6*^*tm2(cre)Lowl*^/J; Jackson Laboratory Stock, 016963) and wild-type mice (C57BL/6J; Charles River). Animals were group housed, up to five per cage, in a temperature (23 °C) and humidity (55%) controlled environment in standard cages on a 12:12 h light/dark cycle with ad libitum access to food and water, unless placed on a food restriction schedule. All food-restricted mice were restricted to 85–90% of their initial body weight by administering one feeding of 2.0–2.5 g of standard grain-based chow per day. All strains used were backcrossed with the C57BL/6J strain. Male mice were used for electrophysiological characterization of LHA–LHb neurons, Patch-seq, optogenetic manipulation and Neuropixels in vivo recordings (Figs. 1–4). Once the cell types were electrophysiologically and molecularly established, as well as in vivo optogenetic manipulation and electrophysiological recordings were performed, we extended our analysis to mice of both sexes. Male and female mice, when possible aged matched and littermates, were used for baseline behavioral testing, and behavioral and electrophysiological characterization upon stress induction (Fig. 5 and Extended Data Fig. 10). The exact number of animals (*N*) for each experiment is reported in the corresponding figure legend.

### Estrous cycle staging identification

Four stages of the estrous cycle were determined by collection and analysis of predominant cell typology in vaginal smears as reported^37^. Samples were collected daily throughout the duration of the experiment, consistently at the same time of the day (9–10 AM). In brief, the sample was collected with ddH_2_O-filled tip at the opening of the vaginal canal. The smear was placed on a glass slide and left at room temperature until completely dried. Cytological staining was performed by placing the dry slide in a Coplin jar containing the crystal violet stain for 1 min, followed by repeated washes in ddH_2_O. The smear was examined under light microscopy to determine the cell types present. Once mice exhibited at least two regular four days estrous cycles, shock protocol was started on the day of metestrus, identified as small darkly stained leukocytes predominate. Electrophysiological recordings and behavioral phenotyping were performed on the metestrus day of the following cycle.

### Tracers and viral constructs

We used red retrobeads (Lumafluor) to visualize LHA–LHb neurons for the characterization of electrophysiological diversity to minimize potential cell-type labeling bias due to viral tropism. Soma harvesting for Patch-seq of LHA–LHb neurons was performed after labeling with retrobeads to prevent potential effects of viral transduction on gene expression profile. Purified and concentrated AAV were purchased from Addgene. Cre-inducible ChR2–mCherry was used (AAV-EF1α-DIO-hChR2(H134R)-mCherry; http://www.addgene.org/20297/) for both anterograde (Addgene viral prep 20297-AAV5) and for retrograde (Addgene viral prep 20297-AAVrg) Cre-dependent optogenetic manipulation of LHA–LHb neurons. Control groups were injected with pAAV-hSyn-mCherry (a gift from Karl Deisseroth; Addgene viral prep 114472-AAV5; Addgene viral prep 114472-AAVrg; http://www.addgene.org/114472/). For TeLC silencing, we used either a retrograde approach via Cre-inducible TeLC (hSyn1-dlox-TeTxLC_2A_NLS_dTomato(rev)-dlox-WPRE-hGHp(A); VVF UZH ID v620) or an intersectional approach combining a retrograde FLPo-dependent TeLC in LHb (ssAAV-retro/2-hSyn-fDIO-TeLC-P2A-dTomato, custom made at https://www.wzbio.com/) with a Cre-dependent FLPo-expressing AAV5 in LHA (AAV5-pEF1a-DIO-FLPo-WPRE-hGHpA; Addgene viral pep 87306-AAV5; https://www.addgene.org/87306/). To combine Esr1:TeLC silencing with optogenetic stimulation of the entire LHA–LHb pathway (Fig. 3), we injected Cre-independent ChR2 in LHA (AAV5-pAAV-hSyn-hChR2(H134R)-EYFP; Addgene viral pep 26793-AAV5; https://www.addgene.org/26973/) together with the intersectional TeLC approach—we injected retrograde FLPo-dependent TeLC in LHb (ssAAV-retro/2-hSyn-fDIO-TeLC-P2A-dTomato, custom made at https://www.wzbio.com/) with a Cre-dependent FLPo-expressing AAV5 in LHA (AAV5-pEF1a-DIO-FLPo-WPRE-hGHpA).

For electrophysiological validation of the TeLC silencing intersectional approach, we co-injected retrograde FLPo-dependent TeLC (ssAAV-retro/2-hSyn-fDIO-TeLC-P2A-dTomato, custom made at https://www.wzbio.com/), retrograde creON/flpON ChR in LHb (pAAV-hSyn Con/Fon hChR2(H134R)-EYFP; Addgene viral pep 55645-AAVrg; https://www.addgene.org/55645/) and a Cre-dependent FLPo-expressing AAV5 in LHA (AAV5-pEF1a-DIO-FLPo-WPRE-hGHpA; Addgene viral pep 87306-AAV5; https://www.addgene.org/87306/). As positive control experiments electrophysiological validation of the TeLC silencing intersectional approach, we injected retrograde creON/flpON ChR in LHb (pAAV-hSyn Con/Fon hChR2(H134R)-EYFP; Addgene viral pep 55645-AAVrg; https://www.addgene.org/55645/) and a Cre-dependent FLPo-expressing AAV5 in LHA (AAV5-pEF1a-DIO-FLPo-WPRE-hGHpA; Addgene viral pep 87306-AAV5; https://www.addgene.org/87306/). We injected in LHA Cre-inducible ChR (pAAV-Ef1a-DIO-hChR2-EYFP; Addgene viral pep 35509-AAV5; https://www.addgene.org/35509/) as an additional positive control. For electrophysiological characterization of LHA–LHb neurons in *Esr1-cre*, *Npy-cre* and *Pv-cre* mice at baseline, as well as for electrophysiological characterization of Esr1^+^ LHA–LHb neurons after stress, we injected retrograde Cre-dependent tdTomato (pAAV-FLEX-tdTomato; Addgene viral prep 28306-AAVrg; http://www.addgene.org/28306/) in the LHb. The helper virus TVA-V5-RG (AAV5-EF1a-DIO-TVA-V5-t2A-Rabies G) and Rabies-EGFP virus were cloned and produced in the Meletis laboratory.

### Stereotaxic injections

All stereotaxic injections were performed on 8–10 weeks old mice. For injections of red retrobeads (Lumafluor) in the LHb, wild-type mice were anesthetized with 5% isoflurane and mounted in a stereotaxic apparatus (Harvard Apparatus). Retrobeads of 50 nl were injected at 100 nl min^−1^ unilaterally using a glass micropipette with a Quintessential Stereotaxic Injector (Stoelting). The LHb was targeted using the following coordinates: 1.65 mm caudal and 0.35 mm lateral to bregma, and 2.55 mm deep from the dura. Mice were killed 4–7 d postinjection for slice electrophysiology or Patch-seq. Viral retrograde labeling of cell-type-specific projection neurons was achieved by injecting 150 nl of AAVretro-EF1α-DIO-hChR2(H134R)-mCherry, AAVretro-FLEX-tdTomato (for anatomical mapping, optogenetic manipulation and in vivo Neuropixels recording), AAVretro-FLEX-tdTomato (for electrophysiological recording after stress induction protocol), AAVretro-hSyn-fDIO-TeLC-P2A-dTomato or AAVretro-hSyn1-dlox-TeTxLC-2A-NLS-dTomato (for TeLC silencing) bilaterally at 50 nl min^−1^. The LHb was targeted using the same coordinates reported above for retrobead injections. For anterograde labeling of LHA neurons, 200 nl of AAV5-EF1α-DIO-hChR2(H134R)-mCherry, AAV5-FLEX-tdTomato or AAV5-DIO-FLPo was injected at 50 nl min^−1^ in LHA unilaterally (for mapping input topography of the LHb) or bilateral (for behavioral experiments upon optogenetic manipulation or TeLC silencing). The LHA was targeted at the peak coordinates of each cell type using the following coordinates: *Pv-cre*, 1 mm; *Esr1-cre*, 1.25 mm; *Npy-cre*, 1.45 mm; *Gal-cre*, 1.55 mm; *Vglut2-cre* and C57BL/6J, 1.4 mm caudal from bregma. LHA injections in all mice were performed 1 mm lateral from bregma, and 4.65 mm deep from the dura. For cell-type projection-specific retrograde tracing using Rabies virus, 100 μl of TVA-V5-RG (AAV5-EF1a-DIO-TVA-V5-t2A-Rabies G) was injected in the LHA and 2 weeks after, 100 μl of Rabies-EGFP was injected into the LHb. For all stereotaxic injections, the glass pipette was left in place for 15 min after the injection and before removal from the brain. Optogenetic experiments were performed 15 d postinjection. TeLC experiments were performed 6 weeks postinjection. For anatomy, mice were killed 15–20 d postinjection. For Rabies retrograde tracing experiments, mice were killed 10 d after the Rabies virus injection.

### Implant surgery

Adult mice were anesthetized with isoflurane (3% for induction then 1–2%). Buprenorphine (0.1 mg kg^−1^ s.c.), carprofen (5 mg kg^−1^ s.c.) and lidocaine (4 mg/kg s.c.) were administered. The body temperature was maintained at 37 °C by a heating pad. An ocular ointment (Viscotears, Alcon) was applied over the eyes. The head of the mouse was fixed in a stereotaxic apparatus (Kopf). Lidocaine 2% was injected locally before skin incision. The skin overlying the cortex was removed, the skull was cleaned with chlorhexidine and the bone was gently cleaned. A thin layer of glue was applied to the exposed skull. A lightweight metal head-post was fixed with light-curing dental adhesive (OptiBond FL, Kerr) and cement (Tetric EvoFlow, Invoclar Vivadent). For optogenetics, two small craniotomies (~300 to 500 μm in diameter) are performed to allow the insertion of the two fibers that are slowly lowered vertically into the brain at the depth for photoactivation (LHb, +1.55 mm AP; 0.95 mm ml from bregma and 2.2 mm depth with 10° angle from the dura, LHA, +1.1 mm ml; 4.25 mm depth from the dura; the AP coordinate was adjusted according to peak coordinates in each of the following genotype: *Pv-cre*, 0.9 mm; *Esr1-cre*, 1.1 mm; *Npy-cre*, 1.2 mm; *Vglut2-cre* and C57BL/6J, 1.1 mm) and cemented to the skull. For extracellular recordings, a chamber was made by building a wall with dental cement along the coronal suture and the front of the skull. Brain regions were targeted using stereotaxic coordinates. After the surgery, the animal is returned to its home cage and carprofen (5 mg kg^−1^ s.c.) was provided for postoperative pain relief 24 h following surgery.

#### Preparation of acute brain slices

All experiments were performed in 250 μm thick coronal slices prepared on a VT1200S vibratome (Leica) in ice-cold artificial cerebrospinal fluid containing (in mM) 90 NMDG, 2.5 KCl, 1.25 Na_2_HPO_4_, 0.5 CaCl_2_, 8 MgSO_4_, 26 NaHCO_3_, 20 d-glucose, 10 4-(2-hydroxyethyl)-1-piperazineethanesulfonic acid (HEPES), 3 Na-pyruvate and 5 Na-ascorbate (pH 7.4). Brain slices were then incubated at 22–24 °C for 60 min in a recording solution containing 124 mM NaCl, 2.5 mM KCl, 1.25 mM Na_2_HPO_4_, 2 mM CaCl_2_, 2 mM MgSO_4_, 26 mM NaHCO_3_ and 10 mM d-glucose (pH 7.4). All constituents were from Sigma-Aldrich. Both solutions were aerated with carbogen (5% CO_2_/95% O_2_). Temperature in the recording chamber was set to 33 °C. Brain slices were superfused with the recording solution at a rate of 4–6 ml min^−1^.

#### Patch-clamp electrophysiology

Neurons were visualized by differential interference contrast (DIC) microscopy on an Olympus BX51WI microscope. Whole-cell recordings were carried out using borosilicate glass electrodes (Hilgenberg) of 5–8 MΩ pulled on a P-1000 instrument (Sutter). Electrodes were filled with an intracellular solution containing 130 mM K-gluconate, 3 mM KCl, 4 mM ATP-Na_2_, 0.35 mM GTP-Na_2_, 8 mM phosphocreatine-Na_2_, 10 mM HEPES and 0.5 mM ethyleneglycol-bis(2-aminoethylether)-*N*,*N*,*N*′,*N*′-tetraacetate, (pH 7.2 set with KOH), with Cl-reversal potential approximately −95 mV. Patch-clamp recordings were carried out on a Multiclamp 700B amplifier (Axon) controlled by DigiData 1550B digitizer and acquired with pClamp (Clampex, Clampfit, v10.6). Current clamp recordings were not corrected for −9.99 ± 0.38 mV liquid junction potential between the intracellular and recording solutions, as measured against a 3 M KCl-electrode. Membrane input resistance (‘Mem resistance’ in MΩ) was calculated using linear regression established between electrotonic voltage responses to current injections (±20 pA from −70 mV holding) of 500 ms. Membrane time constant (*t*, ms) was averaged from 20 successive electrotonic voltage responses to hyperpolarizing (−40 pA) current steps and fitted with a single exponential. ‘AP threshold’ (mV) was defined as the voltage point where the upstroke’s slope trajectory first reached 10 mV ms^−1^. AP characteristics were measured on the rheobasic AP elicited by a 1-s-long current step. ‘AP rise time’ (ms) was the time from the AP threshold to the AP’s peak. ‘Maximum AP up- and AP ‘down stroke’ was determined as the maximum and minimum of the geometrical differential of the AP (mV ms^−1^), respectively. ‘AP half-width’ (ms) was measured at half the maximal amplitude of the AP. Afterpotentials were in certain neuronal types afterdepolarizations (ADP in mV) measured from the most negative fast repolarization of the AP to the most maximal voltage of the ADP. The time to reach this maximal ADP amplitude defined as the ‘ADP rise time’ (ms). Afterpotentials could also have been afterhyperpolarizations (AHP in mV), which could either have a simple ‘convex’ shape without ADP (AP convex AHP amplitude (mV)) or more complex ‘concave’ AHP which followed an ADP. AHP amplitude was in mV and rise times in ms. Firing at rheobase either occurred in the first ‘Firing Rheo first half APs’ or the second ‘Firing Rheo second half APs’ half of the 1-s-long current injection, we measured the number of APs in these time windows. ‘Firing 2×’ labeled parameters derived from the two times rheobasic current injection responses, and ‘Firing MAX’ labeled parameters derived from the voltage response with the highest firing frequency of a neuron. Interspike intervals (ISIs) were measured as frequencies in Hz at the first or second ISI on Firing 2× and Firing MAX traces. Adaptation ratio was calculated as the ratio of the last two ISI relative to the first ‘Adapt 1-last’ and second ‘Adapt 2-last’ ISIs. Firing frequency (Hz) was determined at double-threshold current injections producing spike trains. ‘Firing poststep’ measured the mean of membrane voltage changes in the 150 ms after the 1-s-long current injection step. All parameters were measured by procedures custom-written in Matlab (MathWorks).

### LHA–LHb postsynaptic response

We recorded LHb neurons in voltage-clamp mode found within axon terminals from labeled LHA–LHb neurons. The membrane potential was held at −60 mV during optogenetic stimulation protocols. Light stimulation was delivered through the microscope lens with blue (473 nm) LED light (SPECTRA X, Lumencor). Light intensity measured on the slice was 2.5 mW. The illumination lasted for 1 s (5 ms pulse, 20 Hz). The monosynaptic nature of the evoked postsynaptic currents was confirmed with the combined application of TTX (1 µM; Tocris) and 4-AP (100 µM; Sigma-Aldrich) in the artificial cerebrospinal fluid (aCSF).

#### Patch-seq—cell harvesting for single-cell RNA sequencing

At the end of each patch-clamp routine, we retracted the recording pipette and approached the recorded soma with another pipette (1.8–2.5 MΩ) containing 90 mM KCl and 20 mM MgCl_2_. The soma of each recorded neuron was aspirated into the micropipette within a few seconds by applying mild negative pressure (−50 mPa). This procedure allowed us to decrease time of harvesting, thus minimizing RNA loss. When we broke contact, the recording pipette was pulled out from the recording chamber and then carefully rotated over an expelling 0.2 ml tube, where its content (~0.5 μl) was ejected onto a 4 μl drop of lysis buffer consisting of 0.15% Triton X-100, 1 U μl^−1^ TakaRa RNase inhibitor, 2.5 mM dNTP, 17.5 mM DTT and Smart-seq2 oligo-dT primers (5′-AAGCAGTGGTATCAACGCAGAGTACT30VN-3′) (2.5 μM) preplaced in the end of the 0.2 ml tight-lock tube (TubeOne). The resultant sample (~4.5 μl) was briefly centrifuged (10 s), placed to dry ice and stored at −80 °C, until subjected to in-tube reverse transcription (RT).

### Patch-seq single-cell RNA-sequencing library preparation and sequencing

Before RT, samples were thawed and subjected to 72 °C for 3 min to facilitate complete cell lysis, then cooled to 4 °C. Immediately following the lysis step, 5.5 μl RT mix was added (containing 0.5 μl Superscript II (200 U μl^−1^), 2 μl Superscript II buffer (5×), 0.5 μl DTT (100 mM), 2 μl betaine (5 mM), 0.1 μl MgCl_2_ (100 mM), 0.25 μl TakaRa RNase inhibitor (40 U μl^−1^), 0.1 Smart-seq2 TSO (5′-AAGCAGTGGTATCAACGCAGAGTACATrGrG+G-3′) and 0.05 μl water (per reaction), resulting in ~10 μl reaction volume, and RT was performed at 42 °C for 90 min, followed by 10 cycles of 42 °C for 2 min and 50 °C for 2 min, and finally 72 °C for 10 min. Following RT, 15 μl of PCR mix was added (containing 12.5 μl KAPA HiFi HotStart Ready Mix, (2×) 0.2 μl ISPCR primers (5′-AAGCAGTGGTATCAACGCAGAGT-3′) (100 mM), and 2.3 μl water, per reaction) and cDNA amplification was performed at 98 °C for 3 min, followed by 22 cycles of 98 °C for 20 s, 67 °C for 15 s and 72 °C for 6 min, and a final incubation at 72 °C for 5 min. Subsequently, cDNA libraries were purified using AMPure-XP beads (0.8:1 ratio; Beckman Coulter), quantified by Qubit (Life Technologies) and inspected on an Agilent bioanalyzer 2100 (HS dsDNA kit). Tagmentation and subsequent indexing were done using a custom Tn5 as described earlier^38^. Paired-end (50 bp) sequencing was performed on an Illumina Hiseq2500 instrument, obtaining ~1.5 million reads per cell.

### Extracellular electrophysiological head-fixed recordings

For acute mPFC recordings, two small craniotomies (300–500 μm in diameter) were opened a few hours (>3 h) before the experiment to access the premarked targeted cortical region (+1.95 mm AP; 0.3 mm and −0.3 mm ml). The mice were anesthetized with isoflurane (3% for induction then 1–2%). Buprenorphine (0.1 mg kg^−1^ s.c.), carprofen (5 mg kg^−1^ s.c.) and lidocaine (4 mg kg^−1^ s.c.) were administered. The open craniotomy was covered with Silicone sealant (Kwik-Cast, WPI) and the mouse was returned to its home cage for recovery. Extracellular spikes in the mPFC were recorded using Neuropixels probes (Phase 3B Option 1, IMEC) with 383 recording sites along a single shank covering 3,800 μm in depth. The probe was lowered gradually (speed, ~20 μ s^−1^) in the left hemisphere with a micromanipulator (uMp-4, Sensapex) until the tip reached a depth of ~3,800 to 4,200 μm under the surface of the pia. The probe was coated with CM-DiI (1,1′-dioctadecyl-3,3,3′3′-tetramethylindocarbocyanine perchlorate, Thermo Fisher Scientific), a fixable lipophilic dye for post hoc recovery of the recording location. The coating was achieved by holding a drop of CM-DiI at the end of a micropipette and repeatedly painting the probe shank with the drop, letting it dry, after which the probe appeared pink. The electrode reference was then connected to a silver wire positioned over the pia in a second craniotomy with a second micromanipulator. The probe was allowed to sit in the brain for 20–30 min before the recordings started.

For optotagging experiments, a previously DiD coated (DiD, Vybrant DiD Cell-Labeling Solution, Thermo Fisher Scientific) optic fiber was gradually inserted at a 25° angle caudally of the Neuropixels probe targeting the LHA, coated with DiO (DiO, Vybrant DiO Cell-Labeling Solution, Thermo Fisher Scientific). At the end of the recording, a 473 nm DPSS laser (∼1–2 mW; Cobolt) was used to optogenetically identify LHb-projecting LHA units (>100 pulses at 1–10 ms length were applied for different laser powers).

The signals were filtered between 0.3 Hz and 10 kHz and amplified. The data were digitized with a sampling frequency of 30 kHz with gain 500. The digitized signal was transferred to our data acquisition system (a PXIe acquisition module (PXI-Express chassis, PXIe-1071; MXI-Express interface, PCIe-8381 and PXIe-8381), National Instruments), written to disk using SpikeGLX (B. Karsh, Janelia) and stored on local server for future analysis.

### Three-dimensional whole brain mapping of ex vivo recorded neurons

We defined the D-V and M-L positions as *x* and *y* coordinates, respectively, by comparing our patch pipette position to the Allen Reference coronal atlas using pictures taken with a 4× objective in DIC optics after the recordings. The precision of the estimations was ~0.25 mm. A-P position for *z* coordinates was estimated using the closest landmarks, namely the fornix and the mammillary tract (Extended Data Fig. 2). The *XYZ* coordinates were then plotted into the Allen reference atlas with a 0.5% jitter to decrease overlap from lateral or dorsal view. The mapping was done by custom-written protocols in Matlab.

### Neuron filling, reconstruction and morphometry analysis

Brain slices containing biocytin-filled neurons were postfixed in 4% paraformaldehyde in phosphate buffer (PB) (0.1 M, pH 7.8) at 4 °C overnight. Slices were repeatedly washed in PB and cleared using ‘CUBIC reagent 1’ (25 wt% urea, 25 wt% *N,N,N*′*,N*′-tetrakis(2-hydroxypropyl) ethylenediamine and 15 wt% polyethylene glycol mono-*p*-isooctylphenyl ether/Triton X-100) for 2 d. After repeated washes in PB, biocytin localization was visualized using Alexa Fluor 647-conjugated streptavidin (1:1,000; Jackson ImmunoResearch, 016-600-084). Slices were then rewashed in PB and submerged in ‘CUBIC reagent 2’ (50 wt% sucrose, 25 wt% urea, 10 wt% 2,20,20′-nitrilotriethanol and 0.1% vol/vol% Triton X-100) for further clearing. Slices were mounted on Superfrost glass (Thermo Fisher Scientific) using CUBIC2 solution and covered with 1.5 mm cover glasses. Post hoc dendritic morphology reconstruction and measurements (simple neurite tracing plugin), sholl analysis (Sholl analysis plugin) and somatic measurements were performed in Fiji after z-stack images acquired by a confocal microscope (Zeiss 880). Somatic morphometry was performed on orthogonal projection of z-stack images. Two major perpendicular axes of the cells (*X*, *Y*, 90-degree angle) were used to generate the elliptical (*E*) score (*X*/*Y*, if <1.8 round or triangular soma shape, if >1.8 triangular or elongated soma shape). Twenty-five percent and 75% perpendicular axes to *X* (*Y*1 = major and *Y*2 = minor) were used to generate the triangular (*T*) score (*Y*major/*Y*minor, if <1.2 round or elongated soma shape, if >1.2 triangular soma shape). t-SNE distribution of the LHA–LHb neuronal types based on somatic morphometry was performed using seven somatic parameters (area, *X*, *Y*, *Y*major, Yminor, *E* score and *T* score; as in Extended Data Fig. 2d).

### Immunohistochemistry

Brain sections were cut on a vibratome at 50 μm thickness (Leica VT1000, Leica Microsystems GmbH). Sections were rinsed in PB, blocked for 2 h in 10% normal donkey serum with 0.5% Triton X-100 and then incubated in primary antibody overnight at 4 °C. Primary antibodies were as follows: rabbit anti-Esr1a (1:1,000; Santa Cruz Biotechnology, sc-542), rabbit anti-Galanin (1:1,000; Gift from E. Theodorsson, AB_2314521); rabbit anti-Npy (1:1,000; Peninsula Laboratories International, T-4070); guinea pig anti-Orexin A (1:1,000 Synaptic System, 389 004); chicken anti-V5 (V5, 1:500 dilution; Abcam, ab9113); rabbit anti-cFos (cFos, 1:500 dilution; Santa Cruz Biotechnology, sc-52); Cy5 anti-Streptavidin (1:1,000; Jackson ImmunoResearch, 016-170-084). After extensive washing with PB, immunoreactivities were revealed using Cy5-conjugated secondary antibodies (1:500; Jackson ImmunoResearch, 711-175-152 or 706-175-148) and DAPI (1:50,000) for 2 h at room temperature. All antibodies were diluted in carrier solution consisting of PB with 1% BSA, 1% normal goat serum and 0.5% Triton X-100. Sections were then extensively rinsed in PB, mounted on slides and coverslipped.

### Neuropixels probe track reconstruction

Brain sections were cut on a vibratome at 400 μm thickness (Leica VT1000, Leica Microsystems GmbH). Slices were repeatedly washed in PB and cleared using ‘CUBIC reagent 1’ (25 wt% urea, 25 wt% *N,N,N*′*,N*′-tetrakis(2-hydroxypropyl) ethylenediamine and 15 wt% polyethylene glycol mono-*p*-isooctylphenyl ether/Triton X-100) for 2 d. After repeated washes in PB, slices were incubated with DAPI (1:50,000) for 1 d at room temperature. Slices were then rewashed in PB and submerged in ‘CUBIC reagent 2’ (50 wt% sucrose, 25 wt% urea, 10 wt% 2,20,20′-nitrilotriethanol and 0.1% vol/vol% Triton X-100) for further clearing. Slices were mounted on customized 400 μm thick slides using CUBIC2 solution and covered with 1.5 mm cover glasses. The blue and red channels were imaged at ×4 using a Zeiss 880 confocal microscope. For each brain section, 6–7 *z*-stacks spaced by 50 μm were obtained and down sampled to a 10-μm resolution. The *z*-stacks containing the probe red fluorescent signal were further registered in the Allen CCFv3 and the probe position was estimated using the available ‘SHARP-Track’ pipeline (https://github.com/cortex-lab/allenCCF). As the electrodes are spaced 20 μm apart (center to center) on the shank of the probe, the location along the electrode was transformed into the CCFv3 space based on the orientation and position of the probe track. Unit locations were assigned based on the location of the electrode where that unit had the highest waveform amplitude.

### Image acquisition and analysis

All confocal images were taken using a Zeiss 880 confocal microscope and exported via ZEN black (2.1 SP3 v14.0). CUBIC cleared sections after slice electrophysiology and biocytin staining were acquired as *z*-stacks using a Plan-Apochromat 20×/0.8 M27 objective (imaging settings—frame size, 1,024 × 1,024; pinhole, 1 AU; bit depth, 16 bit; speed, 6 and averaging, 2). For viral expression overview, Plan-Apochromat 10×/0.45 M27 objective was used (imaging settings—frame size, 1,024 × 1,024; pinhole, 1 AU; bit depth, 8 bit; speed, 7 and averaging, 2). For viral expression and immunohistochemistry close up, Plan-Apochromat 20×/0.8 M27 objective was used (imaging settings—frame size, 1,024 × 1,024; pinhole, 1 AU; bit depth, 16 bit; speed, 6 and averaging, 2). The projection of axon terminals in LHb was imaged using a Plan-Apochromat 63×/1.40 Oil DIC M27 objective (imaging settings—frame size, 1,024 × 1,024; pinhole, 1 AU; bit depth, 16 bit; speed, 6 and averaging, 4).

### Input-specific 3D habenula map

Acquisition settings of confocal microscope images were kept identical for all sections within the experiment. The LHb was first delineated by manual drawing of contours on each coronal section. Sections from the four different genotypes were aligned at each unique coordinate along the anteroposterior axis by translation and rescaling before defining a common outline. For each section, pixels within the LHb were labeled in a binary manner as either ‘terminal’ or ‘not terminal’ by thresholding fluorescence level. The LHb was then binned into 4.1 µm^2^ squares and the terminal density of these spatial bins was computed using a two-dimensional Gaussian kernel, resulting in a heatmap of terminal density across spatial bins for each section and each genotype. To account for differences in baseline fluorescence between genotypes, the density was normalized. For visualization purposes, the expression was linearly rescaled between 0 and the maximum density across all sections of a genotype. For further analysis, we used another normalization to better spread the values within the entire dynamic range. Spatial bins were sorted by increasing terminal density across all sections of a genotype and partitioned into 100 sets of equal cardinalities—each set corresponding to one percentile of the data. Then, the LHb was subdivided by assigning each spatial bin to the genotype with the highest percentile density when this value was above the 50th percentile; otherwise, the region was left unassigned. For 3D visualization, we used the 3D meshes from the Common Coordinate Framework v3 (CCFv3) 2017 of the Allen Brain Atlas to define the LHb delimitations. We performed the above-mentioned analysis to assign each voxel from a 3D grid to one of the four genotypes, without the 50th percentile threshold for smoother visualization. We then extracted a 3D volume definition for each genotype using the marching cubes algorithm implementation from the *Rvcg* R package. Visualizations were rendered using the OpenGL implementation from the *rgl* R package.

### In vivo optogenetics

In optogenetic experiments, mice were bilaterally implanted with optical fibers aimed above the LHb terminals (+1.55 mm AP; 0.95 mm ml from bregma and 2.2 mm depth with 10° angle from the dura) or the cell bodies in the LHA (+1.1 mm ml; 4.25 mm from the dura; the AP coordinate was adjusted according to peak coordinates in each of the following genotype: *Pv-cre*, 0.9 mm; *Esr1-cre*, 1.1 mm; *Npy-cre*, 1.2 mm; *Vglut2-cre* and C57BL/6J, 1.1 mm). Optical fibers were homemade using an optic fiber of 200 µm diameter (FG200UEA, Thorlabs) and matching ferrules (CFLC230; Thorlabs). Mice were coupled via a ferrule patch cord (D204-2128, Doric Lenses) to a ferrule on the head of the mouse using a split sleeve (ADAL1-5, Thorlabs). The optical fiber was connected to a laser (MLL-III-447-200mW laser) via a fiber-optic rotary joint (FRJ_1×1_FC-FC, Doric Lenses) to avoid twisting of the cable caused by the animal’s movement. After a testing session, animals were uncoupled from the fiber-optic cable and returned to their home cage. The frequency and duration of photostimulation were controlled using custom-written Arduino script (Arduino IDE) controlled via Bonsai (v2.6.3) or PsychoPy (v2022.1.1). Laser power was controlled by dialing an analog knob on the power supply of the laser sources. Light power was set to 8 mW measured at the tip of the ferrule in the patch cord before each experiment, using an optical power and energy meter and a photodiode power sensor (Thorlabs). Animals showing no detectable viral expression in the target region or ectopic fiber placement were excluded from the analysis. Fiber placement of each mouse included in the analysis is reported in Extended Data Fig. 4.

### Behavioral tests

Mice were handled for three consecutive days prior to behavioral experiments. Mice were acclimated to the behavioral room for 1 h before testing. Animal behavior was recorded with a CCD camera with infrared filter interfaced with Biobserve software (Biobserve GmbH). Red and infrared light were the only light sources present during all behavioral experiments. When multiple behavioral tests were performed within the same subject, 1 week was left between two experiments. For behavioral phenotyping after shock exposure, mice underwent three consecutive tests within the same day, then combined in the SI. Details on behavioral tests^39,40^ can be found in the Supplementary Methods section in the Supplementary Information File.

### Quantification and statistical analysis

#### Statistics and reproducibility

No statistical methods were used to predetermine sample sizes, but sample sizes are similar to the published literature^6,7,21,24,27^. Data distribution was assumed to be normal, but this was not formally tested. All *t*-tests were two tailed. Statistical hypothesis testing was conducted at a significance level of 0.05. All mice were randomly assigned to different groups, and data collection was randomized whenever possible. Data collection and analysis were not performed blind to the conditions of the experiments, unless specified. Data collection and analysis were performed blind to the genotype of the mouse in the Neuropixels experiment. Most behavioral experiments were controlled by automated computer systems/scripts (for example, ARDUINO) and the relative data were collected and analyzed in an unbiased way. Mice were excluded that showed incorrect viral injection (reporter protein), or of the optic fiber(s) outside the area of interest were excluded. All fiber placement of behavioral and Neuropixels experiments was assessed and reported in Extended Data Fig. 5. Only experiments with bilateral successful viral/fiber-optic targeting were kept. For Patch-seq analysis, genes with fewer than 50 counts from the sum of all samples were excluded. In single-cell RNA sequencing, cells with fewer than 1,500 unique molecular identifiers (UMIs), more than 50,000 UMIs and fewer than 500 genes were detected. For the spike data, only units with firing rate of >0.1 Hz and ISI violation of less than 1% were selected. The exact number of animals (*N*) or neurons per unit (*n*) for each experiment is reported in the corresponding figure legend. All representative images shown in the manuscript were replicated in at least three independent observations (mice).

#### Clustering of cell types by intrinsic electrophysiology

For unbiased clustering of neurons based on their electrophysiological properties, we applied successive transformations to normalize the dataset. First, we shifted each parameter positive and started at zero through a translation by the minimum feature value. Then values for each parameter were divided by the sum of the corresponding parameter across the entire dataset and scaled by a factor 10,000. We then applied the log(1 + *x*) transformation and scaled the values for each feature by applying *z* score. We used PCA for dimensionality reduction and selected the first four principal components (PCs). Two clustering algorithms were then applied to unbiasedly identify six clusters, that is the same number of cell types as defined by the expert classification. First, we applied hierarchical clustering with Ward’s method. Then we performed graph-based clustering using the Seurat package implementation (*k* parameter was set to 20 and resolution to 1.5). As each clustering method generates a slightly different partition of the dataset, we combined them by consensus clustering—only cells assigned to the same cluster by both algorithms were kept, and others were discarded (*n* = 19). We rendered two-dimensional visualizations by applying the t-SNE algorithm to the four PCs with a perplexity of 30. The accuracy of clustering methods was defined as the percentage of cells assigned to the same cluster with unbiased clustering and expert-based annotation. The accuracy that can be expected by chance was estimated by randomly shuffling the unbiased cluster assignment 10,000 times and averaging the corresponding accuracy values.

#### Patch-seq analysis

Raw data were adapter and quality trimmed with fastp v0.20.0, using default settings, then pseudo-aligned to GRCm38.p6 protein-coding transcripts from GENCODE vM22 using Salmon v0.14.1 (–libType IU–validateMappings). Shell commands were run in parallel using GNU parallel. Transcript-level estimates were collapsed to gene-level using tximport v1.12.3, then converted to a SingleCellExperiment object using scater v1.12. Outliers were detected batch-wise based on three median absolute deviations from median based on (log10) total read counts, (log10) genes detected and percentage mitochondrial counts (lower tail, lower tail and upper tail, respectively) using scater. Next, gene-specific variances were decomposed into biological and technical components as implemented in scran v1.12.1 using experimental batches as a blocking factor. We defined highly variable genes (HVGs) as the top 500 variable genes, ordered by biological variance component and FDR (FDR < 0.05). Dimensionality reduction using UMAP or t-SNE was performed on the first 50 PCs for HVGs as implemented in scater. Graph-based Louvain clustering was performed on the first 50 PCs for HVGs based on Spearman’s rank correlation cell–cell distances as implemented in scran. For discrete groups, we extracted molecular markers of the electrophysiological cell types in a pairwise combination comparison of the six distinct neuronal types by calculating a log2-fold gene expression difference as the ratio of the transcript per million (TPM)/gene across the six types. For visualization of the markers, we filtered the gene expression to exclude single cells belonging to a cluster where the marker expression was below 20% of the cells. To determine whether the gene expressions across two cell types were statistically significant, we modeled TPMs according to a negative binomial distribution and specified the type of linkage between the variance and the mean as a locally regressed nonparametric smooth function of the mean. This comparison delivered the *P* values, which had been adjusted to account for the multiple testing problem using the Benjamini–Hochberg adjustment with a threshold of 0.05 for the adjusted *P* values (*Q* values), equivalent to consider a 5% false positive as acceptable, and identify the genes that are significantly expressed by considering all the genes with adjusted *P* values below this threshold. Data from Patch-seq were integrated with the Rossi data set (that is, LHA neurons projecting to LHb) using the reciprocal PCA method implemented in Seurat. Briefly, each dataset was log-normalized, and joint integration features were selected from the top 1,000 variable features in each dataset that were scaled and subjected to PCA. Next, data were projected and integration anchors were identified based on reciprocal PCA (a k.weight of 20 was used). After integration, data were rescaled and subjected to PCA, and UMAP dimensionality reduction was performed on the first 30 PCs. Finally, an SNN graph was constructed from 30 PCs and clusters were identified using the Louvain algorithm at a Seurat resolution of 0.5. Weighted 2D kernel densities were calculated using the R/Nebulosa package.

#### Statistics on sex difference of intrinsic electrophysiology in stress

Same intrinsic parameters were extracted for the analysis as for the classification and clustering of LHA–LHb neuronal types. Parameters were compared across cell types and sexes using Data Analysis with Bootstrap-coupled ESTimation (DABEST), a permutation-based graphical estimation that steps away from dichotomic null-hypothesis significance testing^41^. In short, the analysis bootstraps the pairwise comparison of means of randomly shuffled groups, producing a Gardner–Altman plot, which visualizes effect size as the distribution of mean differences that also defines a 95% confidence interval (CI). *P* value reflects the proportion of group comparisons that resulted in a mean difference outside the CI.

#### In vivo optotagged LHA–LHb units

To identify optotagged LHA–LHb units (light responses in >10% of trials in 10 ms bin after laser pulse), the stimulus-associated spike latency test (*α* < 1%) was used. To compare light-evoked with spontaneous spike-waveforms, Pearson’s correlation coefficient (*r* > 0.9) was used.

#### Neuroanatomical analysis

For ChR2–mCherry neuron quantification, following brain sectioning, endogenous signal of mCherry expressing neurons was used by semi-automatic counting using Imaris software, after image exporting via Zen black light (v14.0.0). For axon terminal quantification in the input-specific 3D habenula map, confocal images were acquired maintaining consistent settings within the genotypes. Orthogonal projections were then processed in ImageJ (Fiji) software, where a consistent threshold was applied to transform the fluorescence signal into binary data, therefore rescaled, registered and mapped into a common reference atlas, as detailed. Data are represented as mean ± s.e.m. or as box plots. Single data points were overlaid to the box plots to identify individual neurons. For dendritic and somatic morphometry, to test pairwise differences between groups, we used one-way ANOVA and reported Tukey’s multiple comparisons *P* values in Supplementary Table 3.

#### Optogenetic and behavioral experiments

In all experiments, the significance levels of the data were determined using custom-written Matlab script. Two-tailed unpaired or paired Student’s *t*-test was performed when comparing two independent or dependent groups, respectively. For the comparison between more than two groups, we used one-way ANOVA. To further test pairwise differences between groups, we used Tukey’s Multiple comparisons test. Data are represented as mean ± s.d. or s.e.m. as reported in the figures, or as boxplots. Single data points were overlaid to the box plots to identify individual animals. In the optogenetic stimulation experiments, when both groups were performed, open circle single data points represent somatic stimulation of retrogradely labeled neurons in LHA, while solid gray circles represent terminal stimulation in LHb of anterogradely labeled LHA neurons.

#### Pupillometry

We tracked the pupil of the mice using DeepLabCut (DLC; ref. ^42^) and analyzed the data using custom-written Julia script (v 1.6). We manually labeled the pupil (four points) and the eye (four points) in 2000 frames, uniformly sampled from all mice and blocks. After running DLC on every video, we transformed the video coordinates (in pixels) to a pupil and an eye area (ellipse fitting, pixels^2^). Each pupil area value was *z* scored to block 1 per mouse. For the heatmap visualization, normalized pupil area values were averaged per genotype. For the bar plots and the scatter plots, normalized pupil area values were averaged per genotype and per trial. A significant change in pupil size between blocks per genotype was tested on the trial grand average values as observations using a Mann–Whitney *U* test with Bonferroni correction for multiple comparisons.

#### Spike sorting

The high-pass filtered AP extracellular potential data were preprocessed using common-average referencing: the channel’s median was subtracted to remove baseline offset fluctuations, then the median across channels was also subtracted from each channel to remove artifacts. The data were then automatically spike-sorted with Kilosort 2.0 (https://github.com/MouseLand/Kilosort/releases/tag/v2.0) and then manually curated using the phy2 GUI (https://github.com/cortex-lab/phy). During manual curation, clusters of waveforms showing near-zero amplitudes or nonphysiological waveforms were classified as ‘noise’. Clusters with inconsistent waveform shapes and/or refractory period violations were classified as ‘multi-unit’. The remaining units were classified as ‘good’. These potential good units were finally investigated against spatially neighboring clusters. Units showing similar waveforms, clear common refractory periods and putative drift patterns were subjected to a merge attempt. If the resulting cluster was still showing consistent waveforms and a clear refractory period in their autocorrelogram, the merge was kept. Splits of the clusters were also performed on a few occasions where the principal features of the waveforms showed distinct clusters and two or more groups of waveforms could be identified. Only the ‘good’ units were kept for the following analysis.

#### In vivo electrophysiological data analysis

The spike times were corrected for the temporal drifting along the recording (~10 ms h^−1^) relative to a clock signal registered independently by the PXIe acquisition module and the PCI-6221 card login the behavioral signals. First, the temporal drift between the two devices was measured for each recording. Second, a linear regression was applied to correct the timestamps. Then, only units from four PFC subregions (ACAd, PL, ILA and ORBm) with firing rate >0.1 Hz and ISI violation of less than 1% were selected. Spikes falling into LFP saturation periods were removed with a margin of 1 s before and after saturation. Spike trains were aligned to the trial start and binned at 10 ms resolution for analysis. For the heatmap visualization, the firing rates were averaged per unit across trials and per block, smoothed with a 50 ms Gaussian kernel and *z* scored to the baseline per block. To consistently average the same number of trials per block (50 trials), one trial over two was removed from the block 2 trial pool. The units were then sorted either by PFC region (ACAd, PL, ILA and ORBm), by the genotype of the line they originated, by their peak response time in the 500 ms window following the 10 kHz auditory pure tone stimulus in block 1 or by a combination of the preceding options. The same sorting order was kept across all blocks.

#### Generalized linear model

We used a GLM to describe how each single unit firing activity was related to our four task events (sound 1, optogenetic stimulation, sound 2 and air puff). Each single unit rate was binned at 100 ms and modeled as a linear combination of the four regressors also binned at 100 ms (sounds 1 and 2; 200 ms after sound onset, optogenetic stimulation; 500 ms after stimulation onset, air puff; 500 ms after air puff onset). We used the Matlab function glmfit.m with a Poisson link function. Four β coefficients, for each regressor, were obtained per fitted unit.

#### Tuning scores

For each recording session, we created surrogate data by circularly shifting the firing rates at a random time point during the recording session. This procedure decreases the risk of false positive errors as only the connection between the regressors and the neural activity is broken. To calculate the tuning score, we calculated the β coefficients per unit with the GLM fit previously described and compared the obtained values to the distribution of β coefficients obtained from 1,000 iterations of the same GLM fit on circular-shifted surrogate data. The tuning score of one unit per regressor was calculated as the difference of the true β coefficient from the mean of the null distribution (1,000 β coefficients obtained by circular shift) divided by the standard deviation of the null distribution. We obtained and ranked four tuning scores per unit referred to as its tuning profile. Positive and negative tuning scores can be obtained. We refer to the absolute maximum of these four tuning scores as the primary tuning of the unit (and so for the secondary tuning being the second absolute ranked value by descending order).

Additionally, we classified units as positively substantially tuned to a regressor if the β coefficient of this unit is larger than the 99.9 percentile of the distribution of the β coefficients of circularly shifted shuffled iterations. Reversely, negatively significantly tuned units are obtained when the β coefficient is less than the 0.01 percentile of the shuffled distribution. Comodulated units (that is, modulated by optogenetic activation and air puff) were defined as units strictly having their primary and secondary tuning score substantially tuned (positively or negatively) to the optogenetic and the air puff predictors (in any order). Chance levels were calculated from the marginal probabilities observed for the whole population (*n*_mice_ = 25, *n*_unit_ = 1,945) for each primary or secondary tuning.

#### PC analysis

Spikes of every unit were binned with a 10 ms nonoverlapping window in the interval of (−0.1 s, 3 s) relative to the trial onset. Spiking activity was averaged across trials within each block (50 trials in blocks 1, 3 and 4; 100 trials in block 2). Spike counts were *z* scored and convolved with a Gaussian kernel (sigma = 2). Units were pooled together across mice within each genotype (*n*_mice_ = 10 mice for *Esr1-cre*, *n*_mice_ = 5 mice for other genotypes). To balance the number of units, a random subset of 550 units was drawn for each genotype. We performed the dimensionality reduction using PCA to plot the trajectories of neural activity within blocks for each genotype. For a single block we built a *N* × *T* matrix of neuronal activity, where *N* is the number of units pooled together from four different genotypes (4 × 550), *T* is the number of time bins during the trial. We used PCA (python, scikit-learn *0.23.2*) to project the activity of all genotypes to the common activity space with three PCs, and then projected the activity of each genotype on its corresponding PC loadings to visualize them independently. The procedure was repeated for each of the four blocks. Visual inspection confirmed that randomly selected subsets of 550 units resulted in stable trajectories, therefore Extended Data Fig. 9a shows representative plots.

#### Hierarchical clustering

Trial-averaged firing rates (10 ms bin) of the GLM-fitted units (*n*_unit_ = 1,945) were smoothed with a Gaussian kernel (sigma 0.8) and *z* scored across four blocks. A quality control step was performed—units with less than 200 active (firing rate < 0.5 Hz) bins across all blocks (1,600 bins in total) were excluded from the analysis (*n*_unit_ = 1,788 units remaining). PCA was then applied to the activity matrix and the top 20 PC loadings were extracted for each unit. Hierarchical clustering using the PC loadings as features was then applied (Ward’s method). The obtained dendrogram was cut at the depth of ten children. The resulting 15 activity clusters were given subjective names reflecting their principal primary tuning (‘opto’ for primary tuning to the optogenetic stimulation, and similar for ‘air puff’ and ‘sound’). When a significant modulation (Wilcoxon signed rank test with Bonferroni correction) of the mean 1 s baseline activity preceding the auditory stimulus onset was observed across some blocks, the name ‘state’ was used. ‘opto’ and ‘air puff’ clusters are also referred to as ‘aversive signal’ clusters.

#### Conditioned units

To determine if a unit was conditioned by the aversive stimuli, a set of four Wilcoxon signed-rank tests was run to detect changes in the activity on the 10 kHz pure tone in block 2, the blue noise in block 4 and the optogenetic stimulation and the air puff—each statistical test was performed by comparing the mean firing rate defined as baseline (500 ms before the auditory stimulus, pure tone or blue noise) and the mean firing rate following the selected event (500 ms time window) for each unit across equivalent number of trials (50). Conditioned units displayed substantially (Wilcoxon signed rank test, Bonferroni corrected α of 0.001/4) increased or decreased response to the sound and optogenetic manipulation in block 2 (top) or to the sound and air puff in block 4 (bottom) over the trials within the respective block.

#### Activity modes

From all the task-modulated units, we made an activity matrix where each row is the trial averaged firing rate of each unit (50 ms bin) across four blocks. We next subtracted the per-unit firing rate across all blocks and smoothed each trial averaged firing rate with a Gaussian window (sigma 0.8). Then, we computed four activity modes (directions in the activity space that optimally separate different task events and epochs), which are as follows: the ‘sound’ mode, the ‘opto OR air puff’ mode, the ‘opto AND air puff’ mode and the ‘state’ mode. We used the data from all genotypes to compute the modes and visualize the projections onto them. The ‘sound’ mode was computed as the weights given by a support-vector machine (SVM) network trained to discriminate if a sound occurred or not (in a 200 ms time window including some baseline preceding the sound onset or the activity following the sound onset). This ‘sound’ mode results in a **n** **×** 1 vector containing the SVM weights for each unit where **n** is the number of task-modulated units. The ‘opto OR air puff’ mode, the ‘opto AND air puff’ mode and the ‘state’ mode were constructed similarly. For the ‘opto OR air puff’ and the ‘opto AND air puff’ modes, we used the 500 ms time window following either the optogenetic stimulation onset or the air puff onset to train the SVM to discriminate between the two types of aversive stimuli. For the ‘state’ mode, we trained the SVM on the 1 s baseline activity preceding the sound onset in block 1 versus block 4. The analysis yielded three **n** × 1 vectors of SVM coefficients (**n** is the number of units). We applied QR decomposition to these vectors to form *W*, the **n** × 3 orthogonal matrix (using either the ‘OR’ mode or the ‘AND’ mode).

The trial averaged data per block (*X* = *n* × 4 × *t* matrix where *t* are the time bins) was then projected onto these axes as the dot product *W*^*T*^*X*. For the data per genotype, *W* and *X* were restricted only to the units belonging to one genotype.

#### Decoding (logistic regression)

For decoding, spikes during the baseline period of (−2.1 s, −0.1 s) before the trial onset were binned with a nonoverlapping window of 100 ms. The activity was concatenated across blocks (*N* × 4*T*, where *N* is the number of units in one genotype and *T* is the number of time points in one block of trials) and *z* scored within each unit. Units with no spikes during baseline were removed. We decoded the binary block identity in block 1 versus block 2, or in block 1 versus block 4, independently within each genotype and mPFC subregion to find out how accurately a neuronal population could decode an aversive versus nonaversive state. We randomly selected a different number of units (from 5 to 150) to train the logistic regression (python, scikit-learn 0.23.2) on 50% of time bins and to test it on the remaining 50%, and for each subset, we repeated this procedure 50 times (repeated twofold cross-validation). Therefore, each data point depicts mean and standard deviation of 50 repeats. Dashed line shows chance performance (50%). In some areas, less than 150 neurons were recorded and in such cases, accuracy trace ends at the number of recorded units.

### Reporting summary

Further information on research design is available in the Nature Portfolio Reporting Summary linked to this article.

## Online content

Any methods, additional references, Nature Portfolio reporting summaries, source data, extended data, supplementary information, acknowledgements, peer review information; details of author contributions and competing interests; and statements of data and code availability are available at 10.1038/s41593-023-01367-8.

## Supplementary information

Supplementary information Supplementary Methods and Supplementary Tables 1–10.

Reporting Summary

Supplementary Video 1 Real-time place aversion (rtPA) in *Esr1-cre* mice. Real-time place aversion induced by optogenetic stimulation of the Esr1^+^ LHA–LHb pathway.

Supplementary Video 2 Open field behavior of *Npy-cre* mice. Rearing events in the open field induced by optogenetic stimulation of the Npy^+^ LHA–LHb pathway.

Supplementary Video 3 Neuropixels recordings of the aversive state. Head-fixed internal versus external aversive state conditioning during Neuropixels recording in mPFC.

Supplementary Video 4 Pupil tracking. Tracking pupil with DeepLabCut. Same *Esr1-cre* mouse as in Video 3.

Supplementary Video 5 Looming stimulus presentation in the 8-shaped arena. Behavioral response to looming stimuli in a female mouse 24 h after stress induction.

## Data Availability

We provided source data for Main and Extended Data Figures. The Patch-seq dataset generated in this study is deposited to BioStudies under the accession number S-BSST1069. The Neuropixels dataset generated in this study is deposited into DANDI Archive under the accession number DANDI:000473/0.230417.1502 and is available at the following URL https://dandiarchive.org/dandiset/000473/0.230417.1502. Source data are provided with this paper.

## Source data

Source Data Fig. 1 Statistical source data.
Source Data Fig. 3 Statistical source data.
Source Data Fig. 4 Statistical source data.
Source Data Fig. 5 Statistical source data.
Source Data Extended Data Fig. 1 Statistical source data.
Source Data Extended Data Fig. 2 Statistical source data.
Source Data Extended Data Fig. 3 Statistical source data.
Source Data Extended Data Fig. 6 Statistical source data.
Source Data Extended Data Fig. 7 Statistical source data.
Source Data Extended Data Fig. 8 Statistical source data.
Source Data Extended Data Fig. 9 Statistical source data.
Source Data Extended Data Fig. 10 Statistical source data.

## Code availability

The code generated during this study to analyze the Neuropixels dataset is available at https://github.com/PierreLeMerre/Esr1_NPX_code.
