# Supplementary material for: Esr1+ hypothalamic-habenula neurons shape aversive states
Source: Nat Neurosci. 2023 Jun 22;26(7):1245–55. doi: 10.1038/s41593-023-01367-8 (PMC10322719; doi:10.1038/s41593-023-01367-8)
Supplement: Supplementary file 1 — Supplementary Methods and Supplementary Tables 1–10. [file 41593_2023_1367_MOESM1_ESM.pdf]

nature neuroscience

Article

https://doi.org/10.1038/s41593-023-01367-8

Esr1$^+$ hypothalamic-habenula neurons shape aversive states

In the format provided by the authors and unedited

SUPPLEMENTARY INFORMATION

Supplementary Methods

Real time place aversion test

Mice were placed in a custom-made two compartment behavioral arena separated by a wall with an opening in the middle (50 × 25 × 25 cm black plexiglass) for 10 minutes. The behavioral arena was placed on a transparent plexiglass. The animal behavior was recorded with a camera placed below the arena. The mouse performance was evaluated under three different conditions during two consecutive days. The first day optical fibers were connected to the animal but there was no light stimulation, the second day one of the compartments was paired with light stimulation (40 Hz, 5 ms pulse, 473 nm laser, see Video 1). Right after the test, the stimulation paired side was switched. Mice were tracked using DeepLabCut (42). We manually labeled the base of the tail base, left and right hindlimbs and forelimbs in 1000 frames, sampled from all sessions. After running DLC on every video, we transformed the video coordinates (in pixels) to world coordinates (in cm) using a perspective transform matching the four corners of the box. We based our movement analysis on the tail base point tracked by DLC.

Conditioned place aversion test

Mice were placed in a custom-made two compartment behavioral arena (50 × 25 × 25 cm black plexiglass, paired compartment walls had white on black stripes) and the behavior was recorded as in the real time place aversion. The first day optical fibers were connected to the animal but there was no light stimulation, the second day the animal was forced to spend time in the compartments paired with light stimulation (40 Hz, 5 ms pulse, 1 second on, one second off, 473 nm laser). The third day the optical fibers were connected to the animal but there was no light stimulation, and the animal was free to explore both compartments for 10 minutes.

State induction test by optogenetic stimulation

We adapted a conditioned place aversion (CPA) assay to test the presence and persistence of a behavioral state induced by LHA-LHb pathway stimulation. Mice were placed in a custom-made two compartment behavioral arena (50 × 25 × 25 cm black plexiglass, paired compartment walls had white on black stripes) and the behavior was recorded as in the real time place aversion. The first day optical fibers were connected to the animal but there was no light stimulation. Immediately after the habituation session, the animal was forced to spend time in the compartments paired with light stimulation (40 Hz, 5 ms pulse, 1 second on, one second off, 473 nm laser) for 10 minutes (state induction, Figure 3). Immediately after the state induction session, the animal was free to explore both compartments for 10 minutes (immediate state). The second day (24 h post induction) the animal was placed back to the same arena free to explore both compartments for 10 minutes (sustained state). For the habituation session, the immediate state test and sustained state test the optical fibers were connected to the animal but there was no light stimulation. We manually labeled the base of the tail, left and right hindlimbs and forelimbs in 1000 frames, sampled from all sessions. We based our movement analysis on the tail base point tracked by DLC. After running DLC on every video, we transformed the video coordinates (in pixels) to world coordinates (in cm) using a perspective transform matching the four corners of the box. During the 10 minutes state induction, discrete events of stop-backwards, sharp turns, digging and free rearing (number of events of standing on the hind limbs, far from the arena wall) where manually scored for each second during the stimulated and unstimulated epochs.

Open field test

Mice were placed in a custom-made open field (49×49 cm black plexiglass) for 10 minutes (TeLC silencing experiment, Extended Data Fig. 6ab) or 20 minutes (optogenetic stimulation experiment, Extended Data Fig. 6r). The behavioral arena was placed on a transparent plexiglass. The animal behavior was recorded with a camera placed below the arena. For the optogenetic experiment, the mouse performance was evaluated under alternating laser off/on epochs, starting with 5 minutes off (stimulated epoch: 40 Hz, 5 ms pulse, 1 second on 500 ms off for 5 minutes, 473 nm laser). We manually labeled the base of the tail, left and right hindlimbs and forelimbs in 1000 frames, sampled from all sessions. After running DLC on every video, we transformed the video coordinates (in pixels) to world coordinates (in cm) using a perspective transform matching the four corners of the box. The speed (cm/s) and stationary time were analyzed on the base of the tail tracked by DLC. For the optogenetic experiment, discrete events of wall rearing (number of events of standing on the hind limbs, with forepaws on the arena wall), free rearing (number of events of standing on the hind limbs, far from the arena wall) and discrete grooming events (number of events of mice in sitting position with licking of the fur,

grooming with the forepaws, or scratching with any limb) where manually scored for each second during the stimulated and unstimulated epochs (See Extended Data Video 2).

Free-Access Caloric Consumption Assay
Mice were food-restricted to 85 to 90% of their initial body weight by administering one daily feeding of ~2.5 to 3.0 g of standard grain-based chow (immediately following behavioral experiment, if performed). Water was provided ad libitum. All feeding-related behavioral experiments were conducted at the same time in the middle of the animals' dark cycle (at approximately 14:00). Food restricted mice were placed in a custom made 15 × 15 × 20 cm operant chamber with free access to a bottle containing a 15% sucrose reward for 40 min. Each lick (lick response) was detected, and reward was delivered (rewarded lick, 3 μL reward) with a 1 second timeout. Mice were habituated to the operant chamber and connected to the fibers with no light stimulation. Once stable licking was achieved with light-off sessions (three consecutive days of daily average lick responses within ± 10%), sucrose consumption was monitored for two consecutive days of light-off and two consecutive days of light-on stimulation (40 Hz, 5 ms pulse, 1 second on, one second off, 473 nm laser).

Quantification of cFOS in LHb
In order to evaluate the recruitment of LHb neurons we performed a cFOS IHC and quantified the cFOS+ neurons with the lateral habenula area, after confocal imaging of the LHb throughout the anterior-posterior axes. For optogenetic experiments (Extended Data Fig. 6o-p), mice were placed in a custom-made open field (49×49 cm black plexiglass) where they received 10 minutes of simulation protocol as in state induction test (40 Hz, 5 ms pulse, 1 second on, one second off, 473 nm laser). Mice were sacrificed 30 minutes after the start of the stimulation. For optogenetic experiments, the control group was implanted with optic fibers, placed in the same open field for 10 minutes and optical fibers were connected to the animal but there was no light stimulation. For TeLC experiments, mice were placed in a sound isolated fear-conditioning chamber where they received 5 inescapable, uncontrollable electric foot shocks, at 0.3 mA over 10 minutes with random shock duration ranging from 1 to 3 seconds and unpredictable inter-shock intervals (ITIs), from 1 to 15 seconds. Mice were sacrificed 30 minutes after the start of the foot shock protocol.

Stress induction protocol
In order to induce stress-like state in mice for electrophysiological recordings and behavioral tests, mice were exposed to a stress induction 'training protocol' for 3 days. Stress induction protocol was modified to 'mild foot shock' from what previously described in order to maximize the chance of detecting sexually dimorphic effect and avoiding ceiling effect. Mice were placed in a sound isolated fear-conditioning chamber where they received 360 inescapable, uncontrollable electric foot shocks at 0.3 mA over 1hour with random shock duration ranging from 1 to 3 seconds and unpredictable inter-shock intervals (ITIs), from 1 to 15 seconds. When possible, experiments were performed on pairs of littermates previously housed in the same cage. Control animals were placed in the shocking chamber for 1 h, without being shocked. 24 h after the last shocking protocol mice were assigned to slice electrophysiology or behavioral phenotyping. Animal identity was blinded to the researcher who performed the electrophysiological recordings and the scoring of the behavioral tests.

Stress Index
All behavioral experiments were consistently performed between 9 and 12 AM. The researcher who performed the test was blinded to the mice cohort. In order to build a reproducible index of stress level in mice we build a stress index, combining three behavioral tests. Tests were performed with an interval of 30 minutes, in an increasing level of aversiveness: all animals were first tested in the marble burying test (MBT), then in the looming test (LST) and finally in the forced swim test (FST). The stress index was built combining one parameter for each test using the Euclidean distance of z-scored normalized values. Parameters used: buried marbles (n, in the MBT), time spent in behaviors classified as aversive (s, in the LST), time spent immobile (s, in the FST).

Marble burying test (MBT)
The marble burying test was performed as previously described (39). Briefly, 20 clean glass marbles of diameter 1.5 cm and homogeneous color were disposed of in a 5x4 matrix on a 5 cm deep sawdust without food and water. One by one, animals were placed in the cage for 30 minutes. At the end of the due time, mice were returned to their cage, and the number of buried marbles was scored by two blinded experimenters. Marbles were considered buried if covered by sawdust by at least two-third. Before starting the testing of a new mouse, marbles were cleaned with 70% ethanol and placed in a newly prepared cage.

Looming stimulus test (LST)

30 minutes after the MBT, stressed and control mice performed a modified version of the looming test (40) in a custom designed 8-shaped arena. The looming arena, classically composed of one only squared arena, was here modified to an eight-shaped field, obtained by merging two round arenas, 30 cm diameter; 23.5 height, with black matt walls to prevent reflection of the stimulus. An opening between the two circular chambers allowed free exploration. No shelter was provided, as this arena design allowed for escape to the opposite compartment as a defensive strategy, where the animal behavior was monitored. A monitor was placed on the ceiling of the arena, providing dim lighting from the gray screen of the monitor. As for the rest of the behavioral test, infrared illumination, invisible to the mouse, was provided for video recording. The arena was placed on a transparent plexiglass. The animal behavior was recorded with a camera placed below the arena. Thanks to these modifications, the looming stimulus was reliably repeated three times. The looming stimuli was triggered by the experimenter once the animal was in the center of one of the arenas, as previously described (40). In brief, the stimulus was repeated 15 times with increasing diameter (from 2 to 20 degrees of visual angle) for the first 250 seconds, and then stable at 20 degrees for the remaining 250 ms. The next stimulus was presented when the animal was in the center of any of the two arenas, with a minimum of one-minute interval. Behaviors were scored from video recordings by two blind experimenters. A post-stimuli epoch of 60 seconds after each stimulus was analyzed. Each second was assigned to aversive (escape to opposite compartment, freezing, tail rattling, immobility, periphery) and non-aversive (normal walking, grooming, sniffing) behavior and reported as cumulative time spent in aversive behavior over the three trials.

Forced swim test (FST)

30 minutes after performing the looming test, stressed and control mice were individually placed in a transparent acrylic cylinder (height: 60 cm, diameter: 14 cm) containing 2 L of clear water at 25 ± 1° C for 6 min. The cylinder was placed on a transparent plexiglass. The animal behavior was recorded with a camera placed below the arena. Water was changed between subjects. Delay to immobility (delay in seconds to the first immobility) and time spent immobile (seconds spent floating passively in the water) were manually scored by a researcher blind to the animals' cohort.

Fear conditioning

Mice were placed into a sound isolated TSE Multi Conditioning System where they received five tones followed by mild foot shocks (5 second tone, 0.3 mA foot shock during the last 2 s of the tone) with randomized inter-shock intervals. The following day, conditioning to the tone was tested in the same TSE Multi Conditioning System, placing the mouse in a new context (arena shape and smell) where they received 10 tones (5 seconds tone) with randomized inter-tone intervals.

Acoustic startle test

Mice were placed into a sound isolated TSE Multi Conditioning System to habituate for 5 minutes. Afterward the habituation period they were exposed to 10 tones (5 seconds tone, 70 dB, 10 kHz) with randomized inter-tone intervals.

Head-fixed aversive conditioning protocol

A total of 25 male mice (10 Esr1-cre, 5 Npy-cre, 5 Vglut2-cre and 5 C57BL/6J) were exposed to the head-fixed aversive protocol. The researcher who performed the recordings was blinded to the mice cohort. Mice were first habituated to be head-restrained over a period of three to five days to reduce stress levels. An ambient white noise (70 dB) was played continuously to provide a homogeneous auditory background. Behavioral control and behavioral data collection were carried out with custom-written computer routines using a National Instruments board interfaced through LabView or Matlab (Mathworks). The aversive conditioning protocol was divided into eight blocks. Each block being separated by at least one minute from the others. In the first block, a 10kHz pure tone (sound pressure: 80 dB, duration 200 ms), was presented 50 times with a random 3 s to 9 s inter-trial interval (ITI) coming from a uniform distribution. For the second block, the pure tone was followed by a 500 ms pulse light train with a frequency of 40 Hz. Each light pulse has a duration of 5ms with 1ms sinusoidal ramp in and 1ms sinusoidal ramp out. The light power at the tip of the fibers was 6 mW and was systematically measured and adjusted before every experiment. The delay between the onset of the pure tone and the optogenetic stimulation was 500 ms and 100 trials were presented to the mice with a 6 s to 12 s random ITI. The third block was identical to the first block. For the fourth block,

a new sound (blue noise, sound pressure: 80 dB, duration 200 ms) was followed by a 50 ms mild air puff delivered into the face of the mice. The delay between the onset of the blue noise and the air puff was also 500 ms. Block five to eight consisted of 50 trials each of the optogenetic stimulus alone (same as in block 2) with four ranging increasing light powers (0.3 mW, 2 mW, 6 mW and 10 mW). The behavior of the mice was monitored with a Blackfly camera (Teledyne FLIR, USA) during each block with a sampling frequency of 50 Hz. A patch cable connected to a laser (Cobolt MLD 473 nm) controlled by custom behavioral procedures (Matlab) and interfaced through a PCI-6221 card (NI) was used for light delivery.

Supplementary Tables

Supplementary Table 1. Related to Fig.1

Intrinsic parameters of characterized LHA-LHb cell types.

	FA-Bk		Burst		RS-N		LS-N		LS-W		RS-W	
n	20		49		65		37		40		19	
Mean/std	Mean	Std	Mean	Std	Mean	Std	Mean	Std	Mean	Std	Mean	Std
Rm (MΩ)	166,18	110,57	732,75	320,36	630,30	332,85	906,63	262,15	756,54	355,74	721,03	273,89
Spike latency (ms)	30,0	24,0	114,9	68,9	164,8	80,6	717,1	171,3	591,2	194,9	161,2	38,0
Rheo first half freq	1,65	2,91	135,50	69,04	2,25	2,61	0,29	1,61	0,00	0,00	3,30	5,02
Rheo second half freq	0,00	0,00	0,00	0,00	0,00	0,00	1,93	2,49	2,79	3,23	0,00	0,00
AP thr (mV)	-47,45	7,35	-39,09	3,96	-39,40	3,85	-38,54	3,62	-32,41	6,00	-31,36	5,29
Max upstroke (dt/dV)	510,6	116,6	386,6	103,6	447,2	91,7	408,0	89,2	272,5	109,8	222,4	81,9
AP half-width (ms)	0,38	0,06	0,50	0,16	0,40	0,11	0,57	0,10	1,03	0,35	1,10	0,43
Max downstroke (dt/dV)	-248,8	56,8	-194,4	69,3	-247,9	73,1	-164,1	33,6	-92,3	32,9	-77,5	24,4
Conv AHP ampl	0,63	2,82	15,94	5,13	4,61	8,63	9,85	10,16	15,38	11,76	22,11	5,95
ADP rise (mV)	40,63	25,59	0,19	1,31	3,48	4,45	5,39	7,70	2,45	3,98	0,00	0,00
AHP rise (ms)	2,54	9,28	5,00	23,67	6,28	11,74	7,03	18,21	24,64	35,77	2,43	1,75
Freq ISI 1 (Hz) 2xRheo	107,7	59,6	186,9	63,7	42,3	26,0	18,7	18,9	12,8	11,5	33,5	14,7
Adaptation ISI1 last 2xRheo	15,8	16,6	9,5	7,5	58,1	24,2	225,4	169,1	171,6	166,3	36,1	22,7
Adaptation ISI2 last 2xRheo	19,0	18,6	15,9	23,7	58,8	19,6	156,4	64,7	96,7	24,1	51,3	22,1
Freq MAX (Hz)	164,40	52,45	88,27	47,45	128,43	60,52	63,32	23,66	34,35	13,70	30,79	11,61
Freq ISI 1 (Hz) Max	279,9	78,2	238,0	75,7	163,0	69,4	55,4	50,7	13,9	13,4	61,9	20,8
Freq ISI 2 (Hz) Max	298,4	82,5	232,3	81,9	184,5	74,8	43,9	34,6	36,5	14,3	53,4	14,6
Adaptation ISI1 Last Max	36,2	18,0	30,6	14,5	71,0	25,1	375,8	381,8	404,6	235,0	50,2	23,5
Adaptation ISI2 Last	33,7	16,3	31,7	14,8	60,3	14,0	271,5	248,9	110,5	38,6	49,4	10,3
Post step potential (mV)	-20,04	5,22	-3,45	4,42	-1,78	6,32	0,14	5,53	-1,98	5,07	-0,81	4,33

Supplementary Table 2. Related to Fig.1

Dendritic morphology, dendritic Sholl analysis and somatic morphometry parameters of biocytin filled LHA-LHb neurons by type.

	FA-Bk		Burst		RS-N		LS-N		LS-W		RS-W	
Mean/std	Mean	Std	Mean	Std	Mean	Std	Mean	Std	Mean	Std	Mean	Std
Dendritic morphology												
Total length [mm]	3,395	0,523	2,575	0,452	1,802	0,624	2,709	0,292	1,820	0,427	1,863	0,564
Total branches [n]	19,10	4,40	12,72	3,69	11,31	4,60	15,00	4,24	6,87	2,16	7,60	1,81
Primary branches [n]	2,07	0,27	4,31	0,94	3,81	0,83	4,09	0,30	3,87	0,83	4,40	0,89
Dendritic sholl analysis												
Ending radius	495,00	123,04	413,00	92,02	303,84	124,73	365,55	59,81	435,00	41,05	374,00	104,30
Sum intersections [n]	233,60	36,94	172,20	53,64	121,00	56,73	158,66	38,50	125,12	25,05	126,80	39,97
Mean intersection [n]	4,84	0,70	4,10	1,08	4,06	1,29	4,33	1,30	2,91	0,77	3,37	0,29
Median intersection [n]	5,00	0,81	4,00	1,05	3,84	1,72	4,66	1,87	2,56	1,23	3,30	0,83
Max intersections [n]	10,50	2,67	8,40	2,27	8,30	2,05	8,44	1,23	6,50	1,41	7,41	0,54
Max intersection radius	189,00	114,35	155,00	119,65	113,84	113,91	101,11	74,06	71,25	39,07	46,00	5,47
Ramification index	8,66	3,39	6,20	3,36	6,00	1,92	5,22	2,81	5,47	2,12	4,96	2,41
Enclosing radius	495,00	123,04	423,00	92,02	303,84	124,73	376,66	80,93	435,00	41,05	374,00	104,30
Critical value	8,23	1,70	6,85	1,77	6,66	1,20	7,07	1,60	5,51	1,14	5,95	0,84
Critical radius	204,32	98,41	136,28	98,03	97,37	84,37	126,66	71,96	75,04	27,99	49,47	10,19
Mean value	4,93	0,73	4,16	1,11	4,17	1,32	4,42	1,36	2,96	0,80	3,43	0,30
Somatic morphometry												
Area [μm2]	191,98	38,96	206,25	41,46	185,52	42,33	147,69	28,30	303,96	87,87	204,39	57,37
X [μm]	22,22	3,88	19,30	2,45	18,04	2,96	20,60	2,59	26,21	3,87	23,99	1,89
Y1 major [μm]	8,38	1,14	12,38	2,92	10,42	1,93	8,76	2,30	14,77	3,20	9,13	3,76
Y [μm]	10,71	1,31	12,86	2,87	12,67	2,01	10,59	2,13	14,69	2,32	10,22	1,35
Y2 minor [μm]	7,92	1,00	8,54	1,85	9,70	1,61	7,97	2,08	9,40	1,76	7,76	2,61
T score [Y1/Y2]	1,05	1,05	1,44	0,10	1,07	0,04	1,09	0,04	1,59	0,32	1,15	0,09
E score [X/Y]	2,08	0,32	1,55	0,33	1,44	0,24	1,98	0,28	1,82	0,39	2,18	0,41

Supplementary Table 3. Related to Fig.1
P-values of pairwise Tukey's multiple comparisons within One-Way ANOVA test performed on dendritic morphology and somatic morphometry displayed parameters.

Dendrites total length	FA-Bk	Burst	0,0071	Soma area [um2]	FA-Bk	Burst	0,9732
	FA-Bk	RS-N	0,0455		FA-Bk	RS-N	0,2117
	FA-Bk	LS-N	0,0000		FA-Bk	LS-N	0,9992
	FA-Bk	LS-W	0,0000		FA-Bk	LS-W	0,0000
	FA-Bk	RS-W	0,0000		FA-Bk	RS-W	0,9963
	Burst	RS-N	0,9914		Burst	RS-N	0,0631
	Burst	LS-N	0,0084		Burst	LS-N	0,8913
	Burst	LS-W	0,0277		Burst	LS-W	0,0004
	Burst	RS-W	0,1133		Burst	RS-W	1,0000
	RS-N	LS-N	0,0018		RS-N	LS-N	0,4082
	RS-N	LS-W	0,0073		RS-N	LS-W	0,0000
	RS-N	RS-W	0,0406		RS-N	RS-W	0,2838
	LS-N	LS-W	1,0000		LS-N	LS-W	0,0000
	LS-N	RS-W	0,9999		LS-N	RS-W	0,9767
	LS-W	RS-W	1,0000		LS-W	RS-W	0,0075
Primary dendrites [n]	FA-Bk	Burst	0,0000	Soma T score	FA-Bk	Burst	0,0000
	FA-Bk	RS-N	0,0000		FA-Bk	RS-N	0,9643
	FA-Bk	LS-N	0,0000		FA-Bk	LS-N	0,9996
	FA-Bk	LS-W	0,0000		FA-Bk	LS-W	0,0000
	FA-Bk	RS-W	0,0000		FA-Bk	RS-W	0,7314
	Burst	RS-N	0,9711		Burst	RS-N	0,0000
	Burst	LS-N	0,3933		Burst	LS-N	0,0000
	Burst	LS-W	0,7388		Burst	LS-W	0,1548
	Burst	RS-W	0,9999		Burst	RS-W	0,0013
	RS-N	LS-N	0,9255		RS-N	LS-N	0,9958
	RS-N	LS-W	0,9880		RS-N	LS-W	0,0000
	RS-N	RS-W	0,9695		RS-N	RS-W	0,9770
	LS-N	LS-W	1,0000		LS-N	LS-W	0,0000
	LS-N	RS-W	0,6236		LS-N	RS-W	0,8588
	LS-W	RS-W	0,8065		LS-W	RS-W	0,0000
Total dendrites [n]	FA-Bk	Burst	0,0053	Soma E score	FA-Bk	Burst	0,0008
	FA-Bk	RS-N	0,1874		FA-Bk	RS-N	0,9697
	FA-Bk	LS-N	0,0001		FA-Bk	LS-N	0,0000
	FA-Bk	LS-W	0,0000		FA-Bk	LS-W	0,3989
	FA-Bk	RS-W	0,0000		FA-Bk	RS-W	0,9870
	Burst	RS-N	0,7609		Burst	RS-N	0,0241
	Burst	LS-N	0,9366		Burst	LS-N	0,9448
	Burst	LS-W	0,0047		Burst	LS-W	0,3973
	Burst	RS-W	0,1046		Burst	RS-W	0,0056
	RS-N	LS-N	0,1894		RS-N	LS-N	0,0011
	RS-N	LS-W	0,0001		RS-N	LS-W	0,8789
	RS-N	RS-W	0,0070		RS-N	RS-W	0,8449
	LS-N	LS-W	0,0239		LS-N	LS-W	0,0683
	LS-N	RS-W	0,3183		LS-N	RS-W	0,0005
	LS-W	RS-W	0,9906		LS-W	RS-W	0,3389

Supplementary Table 4. Related to Fig.3
Summary of quantified behavior upon optogenetic manipulation of LHA-LHb pathway in Esr1-cre, Npy-cre, Pv-cre, Gal-cre, Vglut2-cre and C57BL6/J mice.

			Mouse line					
**Test**	**Quantified**	**Figure**	**Esr1-ChR2**	**Npy-ChR2**	**Pv-ChR2**	**Gal-ChR2**	**Vglut2-ChR2**	**control**
rtPA	Aversion	3b	↑	=	=	=	↑	=
State Induction	Aversive behaviors	3g-h	↑	=	n.p.	n.p.	n.p.	=
State Induction	Rearing behavior	3i,	=	↑	n.p.	n.p.	n.p.	=
Immediate and sustained	cPA	3l-m, s6q	↑	=	n.p.	n.p.	n.p.	=
Open field	Distance	S6r,s	↓	↓	=	=	n.p.	=
Open field	Free rearing	3j, S6t-w	n.p.	↑	=	=	n.p.	=
Open field	Wall raring	S6t-w	n.p.	=	=	=	n.p.	=
Open field	Grooming	S6t-w	n.p.	=	=	=	n.p.	=
Open field	Center	S6r	=	=	=	=	n.p.	=
Sucrose consumption	Sucrose consumption	S6ä	n.p.	=	=	=	n.p.	=

Supplementary Table 5. Related to Fig.4
Mean, SD and p-values of Mann-Whitney (two-tailed) test with Bonferroni correction performed on the mean block1-zscored pupil area (number of trials indicated in each column) for each genotype (Vglut2-cre, Npy-cre and Esr1-cre) vs control.

	Block 1 (n = 50 trials)		Block 2 (n = 100 trials)			Block 3 (n = 50 trials)			
Mann-Whitney (two-tailed)	Mean	S.d	Mean	S.d.	P value (bonf. Corr)	Mean	S.d.	P value (bonf. Corr)	P value (bonf. Corr
C57Bl/6J (N = 5)	-0.018	0.380	-0.130	0.516	0.352	-0.090	0.400	0.698	1.781
Vglut2 (N = 5)	0.023	0.353	0.5222	0.386	4.641e-11	0.463	0.288	3.443e-08	2.0498
Npy (N = 5)	0.029	0.417	0.223	0.495	0.105	-0.526	0.435	5.721e-08	2.894e-14
Esr (N = 10)	0.016	0.352	0.611	0.347	9.282e-14	0.751	0.369	4.355e-12	0.021

Supplementary Table 6. Related to Fig.4

Mean, SD and p-values of Mann-Whitney (one-tailed) test with Bonferroni correction performed on the mean tuning scores (absolute value, number of mice indicated in each column) for each genotype (Vglut2-cre, Npy-cre and Esr1-cre) vs control (C57Bl/6J).

	C57Bl/6J		Vglut2			Npy			Esr1		
N (mice)	5		5			5			10		
Mann-Whitney (one-tailed)	Mean	S.d	Mean	S.d.	P value (bonf. Corr)	Mean	S.d.	P value (bonf. Corr)	Mean	S.d.	P value (bonf. Corr)
Pure tone	4.147	0.973	4.079	1.904	1.738	5.138	0.927	0.286	3.559	1.959	2.205
Optogenetics	1.176	0.258	3.337	0.409	0.012	1.126	0.599	1.905	1.896	0.612	0.045
Blue noise	2.253	0.461	1.903	0.863	2.536	2.254	0.304	1.643	1.296	0.782	2.955
Air puff	2.713	0.377	2.246	0.331	2.917	2.222	0.442	2.833	1.713	0.708	2.992

Supplementary Table 7. Related to Extended Data Fig.8

Mean, SD and p-values of Mann-Whitney (one-tailed) test with Bonferroni correction performed on the positive tuning scores (number of units indicated in each column) for each unit type, for each genotype (C57Bl/6J , Vglut2-cre, Npy-cre and Esr1-cre).

Positive tunings												
	C56Bl6/J											
	All units			Wide-spiking waveform units				Narrow-spiking waveform units				
N (mice)	5			5				5				
Mann-Whitney (one-tailed)	Mean	n	S.d	Mean	n	S.d.	P value (bonf. Corr)	Mean	n	S.d.	P value (bonf. Corr)	P value (bonf. Corr)
Pure tone	5.814	218	3.063	5.767	176	3.031	2.678	6.067	35	3.236	1.851	1.716
Optogenetics	3.165	29	1.157	3.138	22	1.091	2.886	3.203	6	1.571	2.032	2.024
Blue noise	3.201	190	1.413	3.201	150	1.387	2.768	3.298	34	1.522	1.897	1.789
Air puff	3.598	247	1.621	3.626	196	1.619	2.662	3.547	44	1.704	2.891	2.705
	VGlut2-cre											
	All units			Wide-spiking waveform units				Narrow-spiking waveform units				
N (mice)	5			5				5				
Mann-Whitney (one-tailed)	Mean	n	S.d	Mean	n	S.d.	P value (bonf. Corr)	Mean	n	S.d.	P value (bonf. Corr)	P value (bonf. Corr)
Pure tone	6.536	285	3.386	6.452	244	3.328	2.483	6.847	33	3.555	2.004	1.790
Optogenetics	4.764	293	2.398	4.717	253	2.319	2.787	4.733	33	2.742	2.024	2.106
Blue noise	3.505	196	1.667	3.451	171	1.647	2.255	3.569	21	1.710	2.445	2.106
Air puff	3.247	292	1.438	3.223	256	1.424	2.203	3.398	31	1.620	1.035	0.8400
	NPY-cre											
	All units			Wide-spiking waveform units				Narrow-spiking waveform units				
N (mice)	5			5				5				
Mann-Whitney (one-tailed)	Mean	n	S.d	Mean	n	S.d.	P value (bonf. Corr)	Mean	n	S.d.	P value (bonf. Corr)	P value (bonf. Corr)
Pure tone	6.399	234	3.278	6.545	200	3.364	2.028	5.259	32	2.318	0.261	0.171
Optogenetics	3.195	31	1.178	3.194	30	1.199	3.000	3.217	1	0	NaN	NaN
Blue noise	3.047	180	1.358	3.185	151	1.339	1.432	2.341	27	1.300	0.178	0.073
Air puff	3070	176	1.269	3.166	148	1.237	1.919	2.594	27	1.359	0.702	0.458
	Esr-cre											
	All units			Wide-spiking waveform units				Narrow-spiking waveform units				
N (mice)	5			5				5				
Mann-Whitney (one-tailed)	Mean	n	S.d	Mean	n	S.d.	P value (bonf. Corr)	Mean	n	S.d.	P value (bonf. Corr)	P value (bonf. Corr)
Pure tone	6.309	123	3.823	6.14	101	3.682	2.442	7.301	21	4.415	1.100	0.917
Optogenetics	3.828	92	1.820	3.745	77	1.873	1.921	4.344	13	1.505	0.433	0.281
Blue noise	2.838	78	1.853	2.735	64	1.879	2.187	3.309	14	1.713	1.075	0.814
Air puff	2.959	134	1.608	2.948	106	1.612	2.743	3.349	27	1.633	2.827	2.673

Supplementary Table 8. Related to Extended Data Fig.8

Mean, SD and p-values of Mann-Whitney (one-tailed) test with Bonferroni correction performed on the negative tuning scores (number of units indicated in each column) for each unit type, for each genotype (C57Bl/6J , Vglut2-cre, Npy-cre and Esr1-cre).

Negative tunings												
	C56Bl6/J											
	All units			Wide-spiking waveform units				Narrow-spiking waveform units				
N (mice)	5			5				5				
Mann-Whitney (one-tailed)	Mean	n	S.d	Mean	n	S.d.	P value (bonf. Corr)	Mean	n	S.d.	P value (bonf. Corr)	P value (bonf. Corr)
Pure tone	-4.989	32	1.663	-4.961	24	1.880	2.240	-5.020	7	0.8370	1.749	1.392
Optogenetics	-3.889	24	1.207	-3.887	19	1.239	3	-4.112	4	1.280	2.303	2.329
Blue noise	-3.990	4	0.4590	-4.160	3	0.3780	2.571	-3.480	1	0	2.400	1.500
Air puff	-4.874	9	0.9550	-4.649	6	0.9270	2.052	-5.324	3	1.022	1.636	1.143
	VGlut2-cre											
	All units			Wide-spiking waveform units				Narrow-spiking waveform units				
N (mice)	5			5				5				
Mann-Whitney (one-tailed)	Mean	n	S.d	Mean	n	S.d.	P value (bonf. Corr)	Mean	n	S.d.	P value (bonf. Corr)	P value (bonf. Corr)
Pure tone	-6.451	44	3.395	-6.271	37	3.022	2.796	-7.723	6	5.567	2.330	2.217
Optogenetics	-7.564	84	4.446	-7.940	72	4.565	1.676	-5.478	11	2.938	0.3410	0.1890
Blue noise	-4.682	10	1.086	-4.784	9	1.100	2.590	-3.770	1	0	2.182	1.800
Air puff	-6.788	24	3.862	-6.342	22	3.525	2.147	-11.465	231	5.629	0.3610	0.2720
	NPY-cre											
	All units			Wide-spiking waveform units				Narrow-spiking waveform units				
N (mice)	5			5				5				
Mann-Whitney (one-tailed)	Mean	n	S.d	Mean	n	S.d.	P value (bonf. Corr)	Mean	n	S.d.	P value (bonf. Corr)	P value (bonf. Corr)
Pure tone	-6.551	31	4.363	-6.689	28	4.427	2.771	-5.336	3	2.690	2.121	2.035
Optogenetics	-6.059	31	2.973	-6.177	26	3.059	2.439	-3.745	3	0.5090	0.272	0.274
Blue noise	-3.632	3	0.2830	-3.586	2	0.3830	3	-3.725	1	0	3	3
Air puff	-6.153	27	2.634	-6.382	22	2.680	2.312	-4.236	4	1.499	0.4460	0.3290
	Esr-cre											
	All units			Wide-spiking waveform units				Narrow-spiking waveform units				
N (mice)	10			10				10				
Mann-Whitney (one-tailed)	Mean	n	S.d	Mean	n	S.d.	P value (bonf. Corr)	Mean	n	S.d.	P value (bonf. Corr)	P value (bonf. Corr)
Pure tone	-6.5910	3	0.848	-6.4330	2	1.1350	3	-6.9080	1	0	3	3
Optogenetics	-5.4010	21	2.9710	-5.6280	19	3.0270	2.2660	-3.2470	2	1.1630	0.628	0.505
Blue noise	-3.0620	1	0	-3.0620	1	0	3	NaN	0	NaN	NaN	NaN
Air puff	-4.6060	9	1.1530	-4.5060	8	1.1910	2.6340	NaN	0	NaN	NaN	NaN

Supplementary Table 9. Related to Extended Data Fig.9

Mean, SD and p-values of Wilcoxon signed rank (two-tailed) test with Bonferroni correction performed on the mean baseline firing rate (number of units indicated in each row) across blocks for each activity cluster identified in Fig. 3g.

	Block1		Block 2			Block 3				Block 4				
Wilcoxon (two-tailed)	Mean	S.d	Mean	S.d.	P value (bonf. Corr)	Mean	S.d.	P value (bonf. Corr)	P value (bonf. Corr)	Mean	S.d.	P value (bonf. Corr)	P value (bonf. Corr)	P value (bonf. Corr)
'Opto' #1 (n = 65 units)	3,752	5,183	4,990	5,841	1,079	5,474	6,047	0,125	0,184	5,270	5,858	2,046	2,671	4,982
'Opto' #2 (n = 93 units)	4,312	5,311	4,598	5,245	3,207	4,938	5,851	2,059	1,273	5,106	5,793	4,788	3,606	4,756
'Air puff' #1 (n = 110 units)	2,829	3,670	3,856	4,290	0,048	4,550	4,929	0,002	4,008E-05	5,039	5,258	1,688	0,227	2,085
'Air puff' #2 (n = 65 units)	2,731	3,441	2,810	3,672	3,467	3,323	3,978	0,996	0,050	4,426	5,100	1,887	0,129	1,079
'State' #4 (n = 150 units)	1,976	2,793	2,362	2,788	0,109	2,886	3,409	0,001	1,132E-12	4,673	5,166	0,705	6,759E-08	1,186E-04
'State' #3 (n = 253 units)	1,964	3,106	2,413	3,294	0,014	3,974	4,817	1,652E-15	6,013E-08	3,272	4,582	1,869E-07	0,021	0,048
'State' #2 (n = 128 units)	3,306	4,658	4,621	5,078	3,222E-04	4,002	5,056	0,120	1,767	3,645	4,970	0,391	0,008	1,493
'State' #1 (n = 195 units)	6,317	9,328	5,446	8,473	1,302	5,139	8,186	0,461	0,047	4,919	8,291	3,731	0,846	2,095
'State' #5 (n = 87 units)	2,273	3,351	2,824	3,844	0,229	3,847	4,639	1,912E-04	2,209E-04	4,137	5,661	0,057	0,048	5,211
'Sound' #1 (n = 72 units)	4,836	5,189	5,450	5,561	2,080	5,987	5,875	0,447	2,773	5,314	5,433	2,322	5,210	1,821
'Sound' #2 (n = 72 units)	3,474	4,423	4,033	4,414	0,823	4,760	4,901	0,059	0,034	5,084	5,065	1,320	0,798	3,910
'Sound' #3 (n = 49 units)	5,449	7,031	6,042	7,164	3,163	7,100	8,165	1,076	0,883	7,695	8,903	2,864	2,025	5,087
'Sound' #4 (n = 33 units)	11,336	7,604	10,753	6,620	5,025	12,342	6,823	2,341	1,171	13,263	7,135	1,866	0,842	3,178
'Sound' #5 (n = 189 units)	6,460	7,075	6,533	7,583	3,926	7,047	8,571	4,687	2,589	7,779	8,983	5,204	1,515	1,908
'Sound' #6 (n = 227 units)	3,075	3,765	3,904	4,477	0,059	4,860	5,232	1,088E-05	2,267E-08	5,722	6,862	0,115	0,003	1,456

Supplementary Table 10. Related to Extended Data Fig.10

Intrinsic parameters of characterized Burst-type and RS-N type Esr1+ LHA-LHb in male and female mice at baseline.

	Burst		Burst		Burst	RS-N		RS-N		RS-N
sex	male		female		Male vs female	male		female		Male vs female
n	37		18		p value	57		34		p value
Mean/std	Mean	Std	Mean	Std		Mean	Std	Mean	Std	
Rm (MΩ)	764.7	323.7	675.7	325.6	0.3439221	751.4	345.1	647.7	317.9	0.1570148
Spike latency (ms)	113.2	74.9	96.6	58.0	0.4126213	216.4	174.5	161.2	97.5	0.09420723
AP thr (mV)	-39.8	4.2	-38.6	3.7	0.3500085	-39.6	3.9	-37.8	4.3	0.06014295
Max upstroke (dt/dV)	406.9	113.7	322.6	101.9	0.01011661	426.9	97.5	402.9	90.5	0.2473053
AP half-width (ms)	0.51	0.18	0.56	0.13	0.2549005	0.46	0.14	0.42	0.13	0.1243075
Max downstroke (dt/dV)	-190.6	65.6	-148.0	58.0	0.02314022	-213.7	81.0	-227.5	82.3	0.4360488
Conv AHP ampl	16.5	5.5	10.1	5.5	0.0001898129	5.04	8.30	8.60	10.2	0.0741382
ADP rise (mV)	0.07	0.44	1.83	6.82	0.1192952	3.51	3.42	3.14	4.79	0.6712469
AHP rise (ms)	2.20	9.26	11.51	37.50	0.1573443	6.63	13.31	9.84	26.81	0.4477606
Adaptation ISI1 last 2xRheo	10.70	6.8	9.0	7.4	0.4134339	57.67	31.81	59.81	30.8	0.7550327
Adaptation ISI2 last 2xRheo	21.5	25.4	23.4	45.0	0.8449555	57.1	24.2	57.3	25.5	0.9749085
Freq MAX (Hz)	67.40	36.01	71.05	32.03	0.7165057	82.2	35.5	131.9	71.5	2.772816e-05
Freq ISI 1 (Hz) Max	205.1	77.6	255.6	82.4	0.03067829	126.6	57.7	166.6	89.1	0.0108358
Freq ISI 2 (Hz) Max	197.5	85.5	224.8	100.3	0.2986648	129.9	58.3	188.5	91.5	0.0003356574
Adaptation ISI1 Last Max	27.6	14.7	25.0	11.2	0.5082283	60.5	30.01	71.1	26.0	0.08826478
Adaptation ISI2 Last Max	30.1	15.6	36.2	38.0	0.3975153	56.4	21.3	60.4	16.5	0.3487923
Post step potential (mV)	-1.78	2.73	-1.32	3.21	0.5827655	-0.60	5.37	-1.05	5.04	0.6890553
