# Supplementary material for: Esr1+ hypothalamic-habenula neurons shape aversive states
Source: Nat Neurosci. 2023 Jun 22;26(7):1245–55. doi: 10.1038/s41593-023-01367-8 (PMC10322719; doi:10.1038/s41593-023-01367-8)
Supplement: Supplementary file 2 — Reporting Summary [file 41593_2023_1367_MOESM2_ESM.pdf]

nature portfolio

Corresponding author(s): Marie Carlén, Konstantinos Meletis

Last updated by author(s): May 18, 2023

Reporting Summary

Nature Portfolio wishes to improve the reproducibility of the work that we publish. This form provides structure for consistency and transparency in reporting. For further information on Nature Portfolio policies, see our Editorial Policies and the Editorial Policy Checklist.

Statistics

For all statistical analyses, confirm that the following items are present in the figure legend, table legend, main text, or Methods section.

n/a	Confirmed	
☐	☒	The exact sample size (*n*) for each experimental group/condition, given as a discrete number and unit of measurement
☐	☒	A statement on whether measurements were taken from distinct samples or whether the same sample was measured repeatedly
☐	☒	The statistical test(s) used AND whether they are one- or two-sided<br>*Only common tests should be described solely by name; describe more complex techniques in the Methods section.*
☒	☐	A description of all covariates tested
☐	☒	A description of any assumptions or corrections, such as tests of normality and adjustment for multiple comparisons
☐	☒	A full description of the statistical parameters including central tendency (e.g. means) or other basic estimates (e.g. regression coefficient) AND variation (e.g. standard deviation) or associated estimates of uncertainty (e.g. confidence intervals)
☐	☒	For null hypothesis testing, the test statistic (e.g. *F*, *t*, *r*) with confidence intervals, effect sizes, degrees of freedom and *P* value noted<br>*Give P values as exact values whenever suitable.*
☒	☐	For Bayesian analysis, information on the choice of priors and Markov chain Monte Carlo settings
☐	☒	For hierarchical and complex designs, identification of the appropriate level for tests and full reporting of outcomes
☐	☒	Estimates of effect sizes (e.g. Cohen's *d*, Pearson's *r*), indicating how they were calculated

Our web collection on statistics for biologists contains articles on many of the points above.

Software and code

Policy information about availability of computer code

Data collection	ZEN black 2.1 SP3 (v 14.0.0.0)<br>pClamp (v 10.6, Clampex , Clampfit)<br>Bonsai (v 2.6.3 https://bonsai-rx.org/)<br>Arduino IDE (v 1.8.19 https://www.arduino.cc/en/software)<br>PsychoPy (v 2022.1.1 https://www.psychopy.org/)<br>Matlab (2018b and above, Mathworks Inc)<br>SpikeGLX (v20190327, https://billkarsh.github.io/SpikeGLX/)
Data analysis	Fiji (simple neurite tracing https://imagej.net/plugins/simple-neurite-tracer/)<br>Zen light black (v 14.0.0.0)<br>SHARP-Track (https://github.com/cortex-lab/allenCCF)<br>SALT test (http://kepecslab.cshl.edu/salt.m)<br>R (4.0.0 or higher; R Foundation; https://www.R-project.org)<br>Python (v 3.7.6. or higher)<br>Julia (v 1.6 and higher)<br>scikit-learn 0.23.2<br>DeepLabCut (https://www.nature.com/articles/s41593-018-0209-y)<br>Seurat (v 4.3.0)<br>GENCODE (vM22)<br>Salmon (v 0.14.1, libType IU --validateMappings)<br>fastp (v 0.20.0)

txImport (v 1.12.3)
scran (v 1.12.1)
scater (v 1.12)
Kilosort (v 2.0 https://github.com/MouseLand/Kilosort/releases/tag/v2.0)
Phy2 (https://github.com/cortex-lab/phy)
DeepLabCut (https://github.com/DeepLabCut/DeepLabCut)

The code generated during this study to analyze the Neuropixels dataset is available at: https://github.com/PierreLeMerre/Esr1_NPX_code

For manuscripts utilizing custom algorithms or software that are central to the research but not yet described in published literature, software must be made available to editors and reviewers. We strongly encourage code deposition in a community repository (e.g. GitHub). See the Nature Portfolio guidelines for submitting code & software for further information.

Data

Policy information about availability of data

All manuscripts must include a data availability statement. This statement should provide the following information, where applicable:

- Accession codes, unique identifiers, or web links for publicly available datasets
- A description of any restrictions on data availability
- For clinical datasets or third party data, please ensure that the statement adheres to our policy

We provided source data for Main and Extended Data Figures. The patch-seq dataset generated in this study is deposited to BioStudies under the accession number S-BSST1069. The Neuropixels dataset generated in this study is deposited into DANDI Archive under the accession number DANDI:000473/0.230417.1502 and is available at the following URL https://dandiarchive.org/dandiset/000473/0.230417.1502

Research involving human participants, their data, or biological material

Policy information about studies with human participants or human data. See also policy information about sex, gender (identity/presentation), and sexual orientation and race, ethnicity and racism.

Reporting on sex and gender	*Use the terms sex (biological attribute) and gender (shaped by social and cultural circumstances) carefully in order to avoid confusing both terms. Indicate if findings apply to only one sex or gender; describe whether sex and gender were considered in study design; whether sex and/or gender was determined based on self-reporting or assigned and methods used. Provide in the source data disaggregated sex and gender data, where this information has been collected, and if consent has been obtained for sharing of individual-level data; provide overall numbers in this Reporting Summary. Please state if this information has not been collected. Report sex- and gender-based analyses where performed, justify reasons for lack of sex- and gender-based analysis.*
Reporting on race, ethnicity, or other socially relevant groupings	*Please specify the socially constructed or socially relevant categorization variable(s) used in your manuscript and explain why they were used. Please note that such variables should not be used as proxies for other socially constructed/relevant variables (for example, race or ethnicity should not be used as a proxy for socioeconomic status). Provide clear definitions of the relevant terms used, how they were provided (by the participants/respondents, the researchers, or third parties), and the method(s) used to classify people into the different categories (e.g. self-report, census or administrative data, social media data, etc.) Please provide details about how you controlled for confounding variables in your analyses.*
Population characteristics	*Describe the covariate-relevant population characteristics of the human research participants (e.g. age, genotypic information, past and current diagnosis and treatment categories). If you filled out the behavioural & social sciences study design questions and have nothing to add here, write "See above."*
Recruitment	*Describe how participants were recruited. Outline any potential self-selection bias or other biases that may be present and how these are likely to impact results.*
Ethics oversight	*Identify the organization(s) that approved the study protocol.*

Note that full information on the approval of the study protocol must also be provided in the manuscript.

Field-specific reporting

Please select the one below that is the best fit for your research. If you are not sure, read the appropriate sections before making your selection.

☒ Life sciences ☐ Behavioural & social sciences ☐ Ecological, evolutionary & environmental sciences

For a reference copy of the document with all sections, see nature.com/documents/nr-reporting-summary-flat.pdf

Life sciences study design

All studies must disclose on these points even when the disclosure is negative.

Sample size	No statistical methods were used to predetermine sample sizes, but our sample sizes are similar to those reported in previous publications (Lazaridis, Mol. Psychiatry, 2019; Lecca, Elife, 2017; Ährlund-Richter, Nat. Neurosci., 2019. Fuzik, Nature Biotechnology, 2016; Allen, Science,

2019.)

Data exclusions

For anatomical/optogenetic/Neuropixel experiments we used histological assessment of viral expression, and viral/fiberoptic targeting to remove data from mice. Only experiments with bilateral successful viral/fiberoptic targeting were kept.
For PatchSeq analysis, genes with fewer than 50 counts from the sum of all samples were excluded.
In scRNAseq, Cells with fewer than 1500 unique molecular identifiers (UMIs), more than 50000 UMIs, fewer than 500 genes detected.
For the spike data. only units with firing rate > 0.1 Hz and ISI violation of less than 1% were selected.

Replication

Due to the number of manipulations and required sample sizes per group, multiple independent cohorts were required to fill our data set. The exact number of animals (N) or neurons/units (n) for each experiment is reported in the corresponding figure legend. All representative images shown in the manuscript where replicated in at least 3 independent observation (mice).

Randomization

All mice were randomly assigned to different groups, and data collection was randomized whenever possible.

Blinding

Data collection and analysis were not performed blind to the conditions of the experiments, unless specified. Equal parameter and process were applied for all groups. For the Neuropixels experiments, data collection and analysis were performed blind to the genotype of the mouse in experiment. Further, most behavioral experiments were controlled by automated computer systems/script (e.g. ARDUINO) and the relative data were collected and analyzed in an unbiased way.

Reporting for specific materials, systems and methods

We require information from authors about some types of materials, experimental systems and methods used in many studies. Here, indicate whether each material, system or method listed is relevant to your study. If you are not sure if a list item applies to your research, read the appropriate section before selecting a response.

Materials & experimental systems

n/a	Involved in the study	
☐	☒	Antibodies
☒	☐	Eukaryotic cell lines
☒	☐	Palaeontology and archaeology
☐	☒	Animals and other organisms
☒	☐	Clinical data
☒	☐	Dual use research of concern
☒	☐	Plants

Methods

n/a	Involved in the study	
☒	☐	ChIP-seq
☒	☐	Flow cytometry
☒	☐	MRI-based neuroimaging

Antibodies

Antibodies used

rabbit anti-Esr1a (1:1000; Santa cruz biotechnology, Cat#sc-542),
rabbit anti-Galanin (1:1000; Gift from E. Theodorsson, AB_2314521);
rabbit anti-Npy (1:1000; Peninsula Laboratories International, Cat#T-4070);
guinea pig anti-Orexin A (1:1000, Synaptic system, Cat#389 004);
chicken anti-V5 (1:500; Abcam; ab9113)
rabbit anti-cFos (1:500, Santa Cruz Biotechnology; sc-52)
Cy5 anti-Streptavidin (1:1000; Jackson ImmunoResearch, Cat#016-170-084).
Alexa Fluor 647-conjugated streptavidin (1:1000; Jackson ImmunoResearch, Cat# 016-600-084).
Cy5-conjugated secondary antibodies (1:500, Jackson ImmunoResearch, Cat#711-175-152 or Cat# 706-175-148)

Validation

The Esr1a primary antibody is validated in this manuscript (Supplementary figure 4). The primary and secondary antibody used in this study were validated by the manufacturers.

Animals and other research organisms

Policy information about studies involving animals; ARRIVE guidelines recommended for reporting animal research, and Sex and Gender in Research

Laboratory animals

This study used several mouse lines (mus musculus) group housed, up to five per cage, in a temperature (23°C) and humidity (55%) controlled environment in standard cages on a 12:12 hours light/dark cycle with ad libitum access to food and water, unless placed on a food restriction schedule. Adult 2-5 months old mice were used. All strains used were backcrossed with the C57BL/6J strain.
Esr1-cre (B6N.129S6(Cg)-Esr1tm1.1(cre)And/J; Jackson Laboratory Stock No: 017911)
Npy-cre (B6.Cg-Npytm1(cre)Zman/J; Jackson Laboratory Stock No: 027851)
Pv-cre (B6;129P2-Pvalbtm1(cre)Arbr/J; Jackson Laboratory Stock No: 008069)
Gal-cre (Tg(Gal-cre)KI87Gsat)
Vglut2-cre (STOCK Slc17a6tm2(cre)Lowl/J; Jax Stock No: 016963)
Wild-type mice (C57BL/6J; Charles River).

Wild animals

The study did not involve wild animals.

Reporting on sex	Male mice were used for electrophysiological characterization of LHA-LHb neurons, Patch-seq, optogenetic manipulation and Neuropixels in vivo recordings (Figures 1-4). Once the cell types were electrophysiologically and molecularly established, as well as in vivo optogenetic manipulation and electrophysiological recordings where performed, we extended our analysis to mice of both sexes. Male and female mice, when possible aged matched and littermates, were used for baseline behavioral testing, and behavioral and electrophysiological characterization upon stress induction (Figure 5 and Extended data Figure 10).
Field-collected samples	The study did not involve samples collected from the field.
Ethics oversight	All procedures and experiments on animals were performed according to the guidelines of the Stockholm Municipal Committee for animal experiments and the Karolinska Institutet in Sweden (approval number N166/15, 155440-2020 and 7362-2019).

Note that full information on the approval of the study protocol must also be provided in the manuscript.
